# The Combination of Chiral Assembly and Chiral Template Effects Boosts Circularly Polarized Luminescence of Perovskite Nanocrystals

**DOI:** 10.1002/anie.202507812

**Published:** 2025-11-22

**Authors:** Mateusz Pawlak, Julia Abramowicz, Nadesh Fiuza Maneiro, Gail A. Vinnacombe‐Willson, Sunghwan Jo, Elie Benchimol, Piotr Roszkowski, Zitao Chen, Da Wang, Guido H. Clever, Luis M. Liz‐Marzán, Agustín Mihi, Lakshminarayana Polavarapu, Wiktor Lewandowski

**Affiliations:** ^1^ Faculty of Chemistry University of Warsaw ul. L. Pasteura 1 Warsaw 02–093 Poland; ^2^ CINBIO Universidade de Vigo, Department of Physical Chemistry Vigo 36310 Spain; ^3^ CIC biomaGUNE Basque Research and Technology Alliance (BRTA) Donostia‐San Sebastián 20014 Spain; ^4^ Biomedical Research Networking Center Bioengineering Biomaterials and Nanomedicine (CIBER‐BBN) Donostia‐San Sebastián 20014 Spain; ^5^ Institute of Materials Science of Barcelona (ICMAB‐CSIC) Campus de la UAB Bellaterra 08193 Spain; ^6^ Department of Chemistry and Chemical Biology TU Dortmund University Otto‐Hahn‐Straße 6 44227 Dortmund Germany; ^7^ Guangdong Provincial Key Laboratory of Optical Information Materials and Technology, South China Academy of Advanced Optoelectronics, Institute of Electronic Paper Displays South China Normal University Guangzhou 510006 China; ^8^ Ikerbasque Basque Foundation for Science Bilbao 48009 Spain

**Keywords:** Chirality synchronization, Nanocomposites, Perovskites, Templated assembly

## Abstract

Perovskite nanocrystals (PNCs) exhibiting circularly polarized luminescence (CPL) represent a promising class of materials for display and light communication technologies, owing to their emission covering the entire visible range with near‐unity photoluminescence efficiency. However, these materials suffer from low selectivity in the handedness of the emitted light, with most studies focusing on green emission. We address these issues by exploiting and broadening the scope of interactions between achiral PNCs and chiral organic templates. For this purpose, we select three types of PNCs with red, green, and blue emissions and introduce them into a chiral liquid‐crystalline matrix in the form of composite thin films. Electron microscopy confirmed the assembly of PNCs within nanoscale gaps formed by supramolecular, liquid crystalline structures. The obtained composites displayed a CPL dissymmetry factor *g_lum_* up to ≈ 0.24. The highly dissymmetric CPL properties were found to result from an interplay between two effects: *chiral assembly* of PNCs within a chiral environment (intrinsic) and the *selective filtering* by the chiral matrix. This system enables control over the dominant factors by adjusting the CPL spectral region and type of particle assembly, providing thin film materials with highly dissymmetric and spectrally tunable CPL responses.

## Graphical abstract

We show that the hierarchical order of perovskite nanocrystal assemblies formed within a nanostructured, chal liquid‐crystalline template exhibits extremely high circularly polarized luminescence (CPL). This is due to the helical arrangement of PNCs in the template voids and capitalizes on the inherent electrooptic properties of the template. Ultimately, this enables the fabrication of CPL‐active thin films with practically relevant chiroptical functionality.

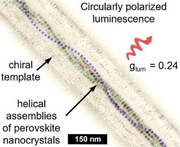

## Introduction

Imparting chirality onto light emitters can help advance optoelectronic, display, and optical communication technologies.^[^ ^1^, ^2^ ^]^ Among others, perovskite nanocrystals (PNCs) have recently emerged as potential sources of chiral light. They feature high photoluminescence quantum yields,^[^ ^3^ ^]^ tunable colors across visible range,^[^ ^4^, ^5^ ^]^ and long exciton lifetimes,^[^ ^6^ ^]^ making them efficient light emitters. However, achieving high CPL dissymmetry remains non‐trivial, despite the PNC electronic structure being sensitive to the local environment.^[^ ^7^, ^8^, ^9^ ^]^ The most common methods to impart chirality to PNCs^[^ ^5^, ^10^ ^]^ rely on organic materials. Chiral low‐molecular‐weight organic compounds can, for example, serve as particle ligands or be incorporated into the crystal structure of PNCs, causing chiral distortions,^[^ ^11^ ^]^ but this strategy is limited by low dissymmetry levels and extensive experimental effort. An alternative approach relies on combining readily available achiral PNCs with chiral, nanostructured ensembles of organic molecules.^[^ ^12^, ^13^, ^14^, ^15^, ^16^, ^17^, ^18^ ^]^ In the simplest example, the organic part exhibits a photonic bandgap and thus selective absorption/reflection/scattering, leading to *selective filtering* of the achiral emission from PNCs.^[^ ^19^ ^]^ The organic ensemble can also act as a matrix into which PNCs are doped, potentially inducing circularly polarized luminescence, e.g., from *chiral assemblies* of achiral PNCs.^[^ ^20^, ^21^, ^22^ ^]^ The organic matrix‐based approach is particularly appealing for a wide range of applications due to the simplicity, ease of upscaling, possibility of flexible film formation,^[^ ^23^ ^]^ and tunable emission color.^[^ ^24^ ^]^ However, challenges persist in achieving efficient induction of circular polarization, with still missing examples of nanoscale assemblies with dissymmetry factors larger than 0.1.

**Figure 1:**
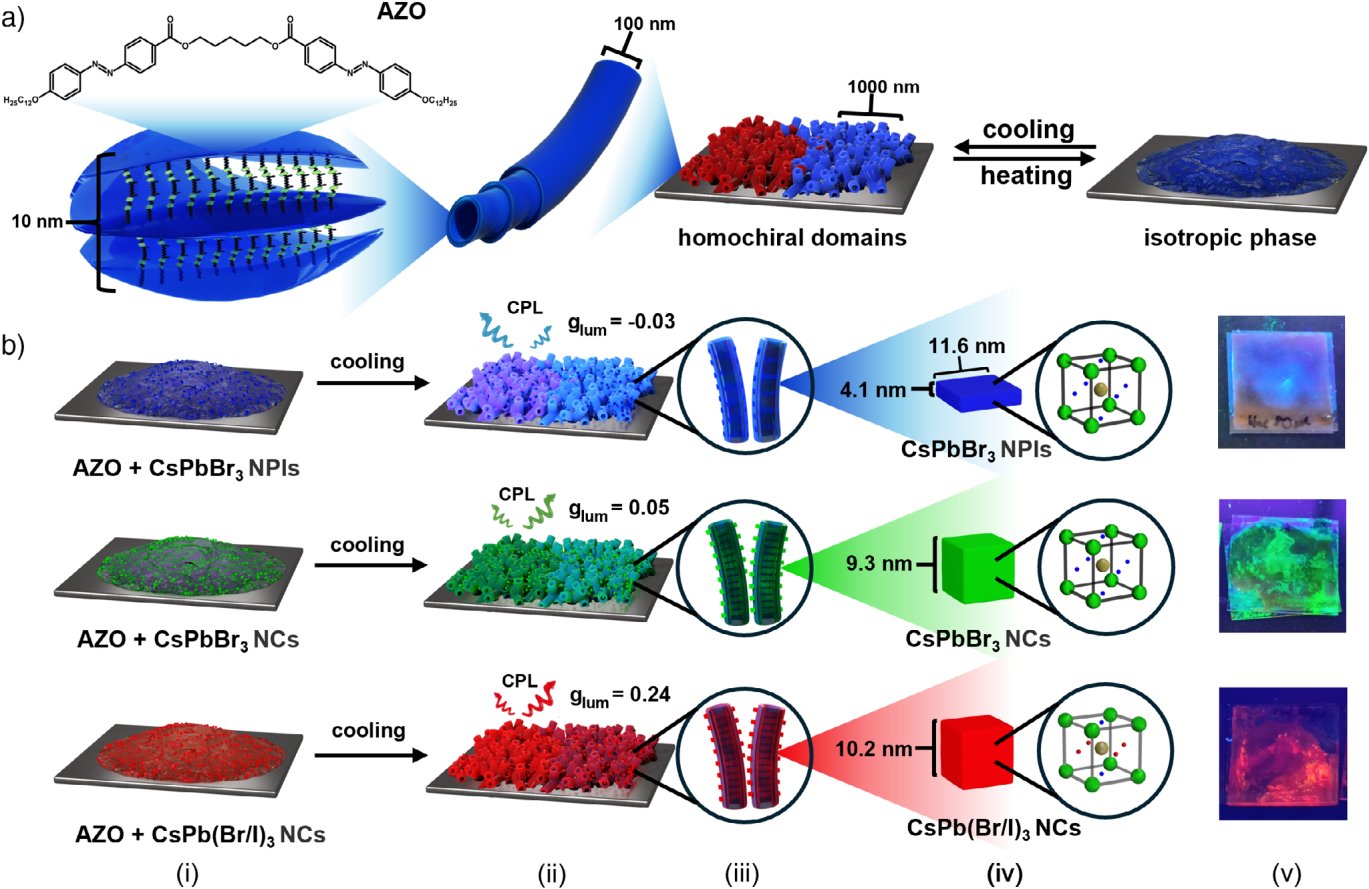
Scope of the work. a) Scheme of the AZO template for the assembly of perovskite nanocrystals (PNCs), highlighting the multiscale structure of the film: AZO dimers (chemical formula shown) assemble into layers, which twist into nanotubes (NTs). The growth of NTs is avalanche‐like, with preserved handedness when growing from a single crystallization point. Since AZO has no chiral center, thin films made of NTs comprise domains of right‐ and left‐handed NTs (depicted with red and blue colors, more detailed analysis and model of crystallization process is presented in Supporting Information Note 1 and Figures S2–S10). b) Fabrication of chiral assemblies of PNCs in the AZO organic matrix, leading to induced CPL. (i) co‐drop casting of PNCs and organic template followed by melting; (ii) cooling yields homochiral domains (indicated by the slightly different coloring of the right and left parts of the thin film). *g_lum_* values for one handedness of the domain are provided; note that the sign changes depending on the spectral region, revealing that various phenomena dominate the CPL induction mechanism for different spectral regions. Zooming into the thin films is schematically shown to: (iii) highlight the hierarchical structure of the films, indicate PNC assembly in and on NTs, and (iv) indicate the types of PNCs used. On the right‐most panels, (v) optical images of the chiral nanocomposite thin film are provided for each sample (under UV illumination, λ = 365 nm).

Here, report a successful strategy leading to CPL dissymmetry factors reaching ≈ 0.24, mediated by the synergistic interplay of two phenomena: 1) *chiral assembly* of PNCs in a chiral environment, where dipole‐dipole interactions, chiral energy transfer, and local symmetry breaking lead to intrinsic circular polarization of emitted light; and 2) *selective filtering* by the template, which acts as a circularly dichroic filter during light propagation. We achieve this through the combination of PNCs within a self‐assembled chiral matrix, namely a dimeric mesogenic azobenzene derivative (AZO, Figure 1a,b). The AZO forms 3D chiral nanotubes (NTs), which become a sponge‐like, porous structure with strong circular dichroism, enabling it to act as a selective filter. At the same time, the porous structure provides numerous voids available for PNCs to assemble (both inside and between nanotubes), leading to chiral assemblies of PNCs with sizes exceeding the wavelength of visible light. Using different PNCs as dopants, we show that CPL can be obtained in various spectral ranges, ultimately covering the entire visible spectrum. We use red‐, green‐, and blue‐emitting PNCs: CsPbBrI_2_ nanocubes, CsPbBr_3_ nanocubes, and CsPbBr_3_ nanoplatelets, respectively. The doped PNCs define the color of the emission, while the template controls chiroptical modulation. Durability of the films, allows us to avoid the problems typically related to cholesteric‐based systems, in which dissymmetry factors higher than 0.1 can be achieved^[^ ^25^, ^26^ ^]^ but are fluid‐like an sensitive to high temperature. Detailed chiroptical characterization shows that different phenomena, either *chiral assembly* or *selective filtering*, dominate the induced CPL at different spectral regions. Through experiments and a simple model, we quantify CPL as *g*_lum_ ≈ *g*_int_ + *g*_filter_, where *g*_int_ corresponds to intrinsically polarized emission (*chiral assembly* in a chiral environment), while *g*_filter_ corresponds to *selective filtering* by the chiral matrix. Overall, we demonstrate that the combined impact of chiral nano‐confinement and locally enhanced chiroptical properties of the matrix can yield highly dissymetric circular polarization of PNC luminescence, tunable via particle type and emission wavelength.

## Results and Discussion

Our approach involves a chiral matrix formed by a liquid crystal with multiscale hierarchical chiral structure. On the molecular level, this compound (α,ω‐bis(4‐n‐dodecyloxyazobenzene‐4′‐carbonyloxy)pentane, AZO) comprises two rigid azobenzene moieties connected by a flexible alkyl linker with an odd number (five) of carbon atoms (Figure 1a). As confirmed by density functional theory calculations, the two lowest energy conformers of this molecule are enantiomers.^[^ ^27^ ^]^ These chiral conformers readily self‐assemble into 5 nm thick layers, within which the molecules are polar and tilted, thus introducing chirality on the level of a single layer. Because these layers are strained, under selected crystallization conditions they conform twisted nanotubes with a diameter of ∼150 nm and a length of ∼500–2000 nm. At the highest organization level, nanotubes may further assemble into chiral aggregates, forming a porous, sponge‐like structure (Figure 1a). This combination of chirality on various length scales (chiral nanotubes and nanotube assembly) yields unique chiroptical properties to the matrix, e.g., evidenced by strong circular dichroism in the visible (*g*‐factor in extinction ∼10^−1^ for all wavelengths in the range 400–700 nm, as confirmed by Mueller Matrix Polarimetry, Figure S1)^[^ ^28^ ^]^ This nanotube matrix was selected as a template to guide the assembly of our PNCs (Figure 1b), as discussed below.

**Figure 2:**
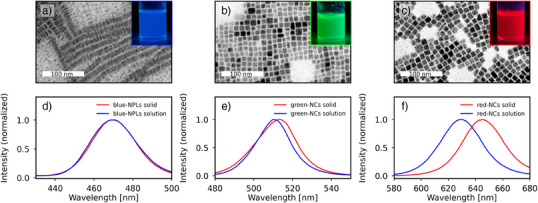
Characterization of PNCs used for the fabrication of composite films. From left to right: blue‐NPLs, green‐NCs, red‐NCs. a)–c) TEM images with insets showing photographs of each PNC dispersion under UV‐illumination; d)–f) Steady‐state photoluminescence spectra for the different PNCs excited at a wavelength of 345 nm.

To obtain composite films exhibiting CPL across the visible spectrum, we doped the chiral host with three different PNCs: 1) CsPbBrI_2_ nanocubes (referred to as red‐NCs), 2) CsPbBr_3_ (green‐NCs), and 3) CsPbBr_3_ nanoplatelets (blue‐NPLs). Blue‐NPLs and green‐NCs were prepared in octadecene using a previously published protocol,^[^ ^29^ ^]^ via a hot injection method using Cs_2_CO_3_, PbBr_2_ precursors, along with oleic acid and oleylamine ligands. Red‐NCs were obtained from green‐NCs via anion exchange upon addition of a PbI_2_ solution in toluene.^[^ ^30^ ^]^ The detailed protocols are provided in the Materials and Methods section, Supporting Information. We examined the morphology of as‐obtained PNCs using X‐ray diffraction (Figures S11 and S12 and Supporting Information Note 2) and scanning and transmission electron microscopy (TEM, SEM). As expected, NCs displayed a cubic morphology, with average side length of 9.3 ± 1.4 nm for green‐NCs and 10.2 ± 0.7 nm for red‐NCs (Figure 2b,c). On the other hand, blue‐NPLs formed flat flakes with a strong tendency to self‐assemble into stacks oriented perpendicular to the substrate surface (Figures 2a, and S13). The thickness of the NPLs was 4.1 ± 0.5 nm, with an edge length of 11.6 ± 1.2 nm. We also examined the emission properties of all three types of perovskite NCs by steady‐state photoluminescence spectroscopy.

PNCs were characterized both in solution and as upon spin casting and drying on a glass substrate. When in solution and excited with 335 nm UV light, all three PNC samples showed bright emission, with maxima at 650, 510, and 470 nm for red‐NCs, green‐NCs, and blue‐NPLs, respectively (Figure 2d–f). Since we aimed at working with chiral thin films in the solid state, we studied the influence of drop‐casting and drying on the PNC emission properties. For blue‐NPLs, we did not observe any effect on the emission maximum, whereas the emission bands of green‐NCs redshifted by ∼3 nm and those of red‐NCs redshifted by ∼20 nm.

Chiral nanocomposites with the different halide PNCs were prepared by mixing PNC dispersions in toluene with the liquid crystalline matrix. The amount of AZO in each sample was 1.68 mg, whereas the volume of (freshly synthesized) PNC dispersions was varied in the range of 10–400 µL. We used UV–vis spectroscopy to assess and maintain constant concentrations of PNC dispersions throughout all experiments (Supporting Information Note 3). The mixtures were drop‐casted onto a glass substrate and heated up to 115 °C to remove the solvent and melt the organic matrix. Subsequently, the samples were covered with a second glass slide, pressed, and cooled down to room temperature, at a rate of 2 K min^−1^. Upon cooling, the liquid crystals assembled forming a chiral sponge‐like structure that would trap PNCs in nanosized spaces, both inside and between nanotubes. We hypothesized that such a trapping of PNCs might lead to two phenomena. One is PNCs chiral arrangement, translating into chiral dipolar interactions between nanocrystals. Second is the interaction of PNCs with an asymmetrically distributed electric field in the vicinity of chiral NTs (near‐field interactions).

The prepared films showed bright emission, with colors corresponding to the photoluminescence of pure PNCs (Figure S14). Simultaneously, AZO formed extended homochiral domains, which could be distinguished using polarized optical microscopy (POM) based on their optical rotation. Specifically, homochiral AZO domains induce the rotation of linearly polarized light in opposite directions: clockwise‐rotating domains were marked as P and counterclockwise‐rotating ones as M (Figures S15 and S16). We thus conclude that the annealing procedure does not degrade the PNCs, nor do the PNCs hinder the assembly of the organic matrix into chiral nanotubes. Note that when performing structural and optical investigations, we covered entire samples to verify uniform optical response within a single sample and the reproducibility of results among different samples. Note that this sampling scheme also applies to samples containing 10–50 µL of PNC dispersion, for which areas with more and less intense photoluminescence are easily noticeable (Figure S14).

To reveal the organization of the PNCs around the twisted tubes, we investigated the internal structure of the films using both SEM and TEM. The thorough SEM and TEM inspection of these challenging samples is fully described in the Supporting Information Note 4.

We present first a general overview of the images. In all the samples, the presence of nanotubes was evident, confirming that PNCs do not prevent the assembly of organic material. This conclusion is in agreement with POM measurements. We additionally note that PNCs were well‐distributed within all of the probed materials. Apart from the areas located close to the glass slides visualized in SEM images (Supporting Information Note 4), we did not observe any phase‐separated PNC aggregates (hundreds of nm).  PNCs were consistently found to be in direct contact with the LC nanotubes. Overall, these results show that PNCs exhibit chemical compatibility with the organic matrix, likely because of the presence of alkyl ligands on the PNC surface, with affinity toward alkyl terminal chains of AZO molecules forming the walls of chiral nanotubes. PNCs were evenly distributed in the molten state in the film.

**Figure 3:**
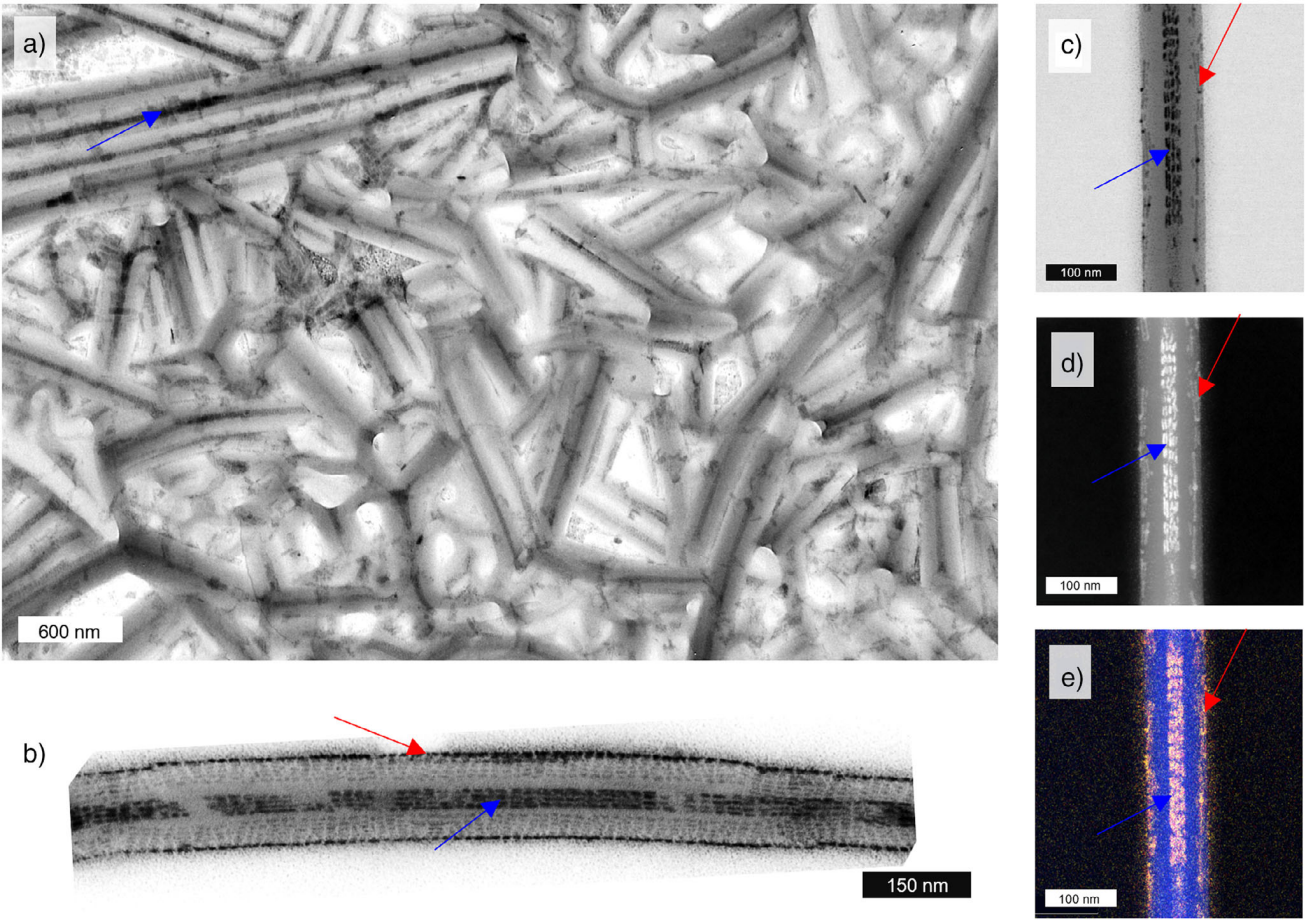
Electron microscopy examination of morphologies formed by blue‐NPLs: a) TEM image of a large area of the sample, b) TEM image of an AZO nanotube filled with blue‐NPLs stacked inside and surrounding the AZO nanotube, c) TEM image, d) HAADF‐STEM image, and e) TEM‐EDS map showing the elemental distribution (blue: C, red: Pb, yellow: Br) in blue NP assemblies with AZO nanotubes. Blue arrows point to areas where PNCs are inside AZO NTs whereas red arrows point to PNCs on the outer surface of AZO NTs.

**Figure 4:**
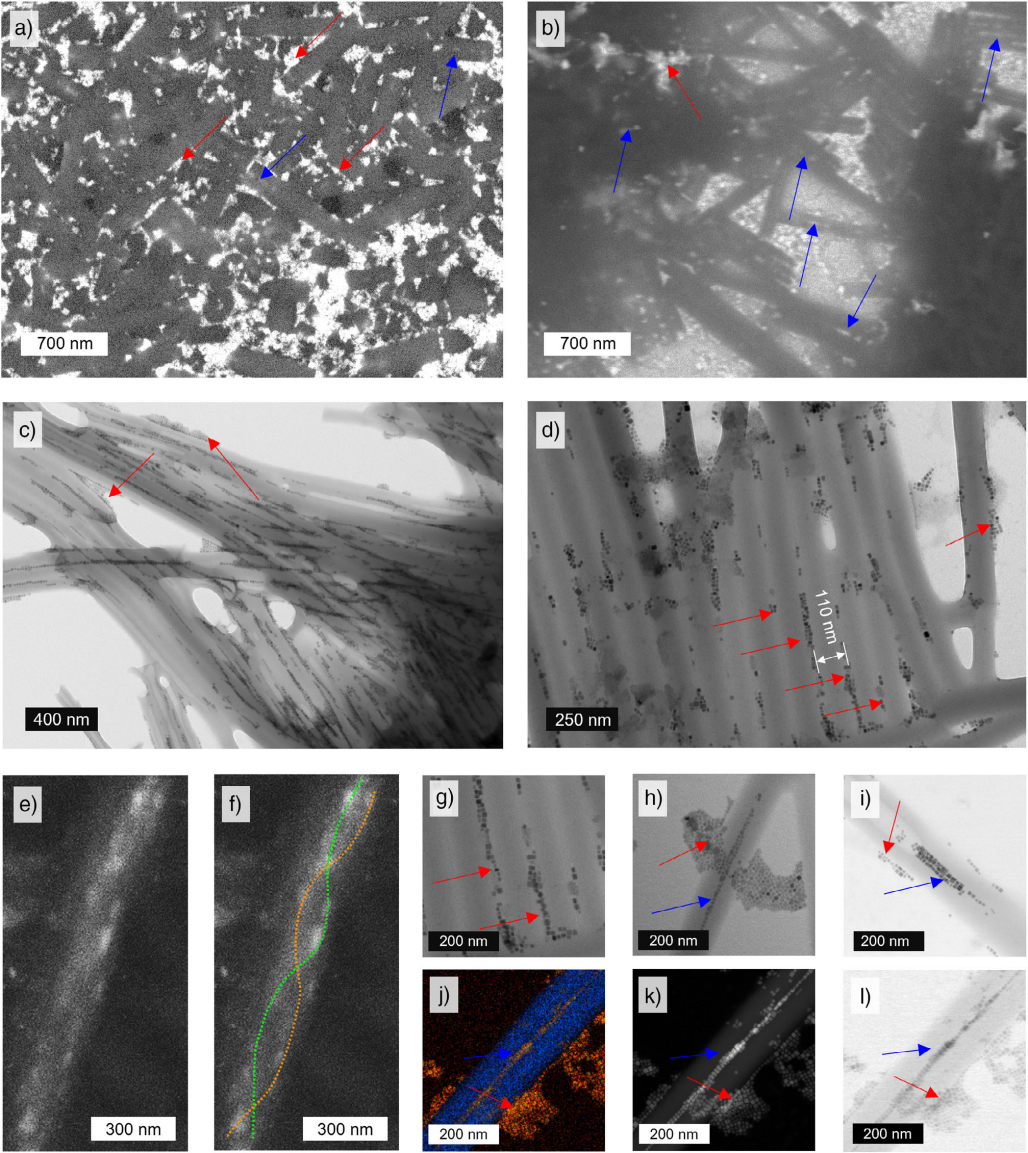
Electron microscopy examination of morphologies formed by red‐NCs in thin films of an AZO matrix, including SEM a), b), e), f) and TEM images c), d), g)–l). a), b) SEM images of PNCs distribution on the surface of a 10 µm thick sample; c), d) TEM images of PNCs assemblies in AZO matrix, observed over large areas; e), f) SEM images of helically assembled PNCs inside AZO nanotubes, dashed lines are guides for the eye; additional TEM images showing the helical arrangements in Figure S4; g) helical assemblies inside AZO nanotubes; h) an AZO nanotube containing a helical assembly inside and surrounded by disordered NCs; i) NCs stacked inside AZO nanotubes which partially broke along the long axis; j) TEM‐EDS map showing elemental distribution (blue: C, red: Pb, green: Cs, yellow: Br); k) HAADF‐STEM image; l) TEM image of an AZO nanotube filled with assembled NCs and surrounded by disordered NCs. Blue arrows point to areas where PNCs are inside AZO NTs, whereas red arrows point to PNCs on the outer surface of AZO NTs.

Upon further analysis, we found that PNCs were mainly located at the surface of individual nanotubes (Figures 3a,b and 4c,f), and inside the hollow interior of nanotubes (Figures 3a and 4c,e,f). Focusing on PNCs inside nanotubes, we identified two modes of organization: 1) seemingly linear assemblies of NPLs or cubic NCs (Figures 3b and 4j,k) and, less dominant, 2) helical (chiral) assemblies of NCs (not observed for NPLs, Figures 4e–l, and S23). To unequivocally confirm that the observed structures arise from PNC assembly, we conducted TEM coupled with energy‐dispersive spectroscopy (EDS‐TEM) and high‐angle annular dark‐field scanning transmission electron microscopy (HAADF‐STEM), allowing us to distinguish inorganic PNCs from organic nanotubes (Figures 3e and 4j,k). It should be noted that annealing could potentially lead to the transformation of nanocrystals into nanowires,^[^ ^31^, ^32^ ^]^ but we did not observe this effect. While most PNCs were unaffected by the film fabrication process, a small fraction of aggregated NPLs or cubic nanocrystals were found (Figures S17–S25). As discussed below, this effect may partially contribute to varying the non‐polarized emissive properties of PNCs in composite films compared to those in solution. Overall, electron microscopy characterization confirmed that PNCs assemble into chiral structures inside and on the surfaces of chiral nanotubes. Both assembly modes have the potential to induce chiral optical properties on PNCs.

**Figure 5:**
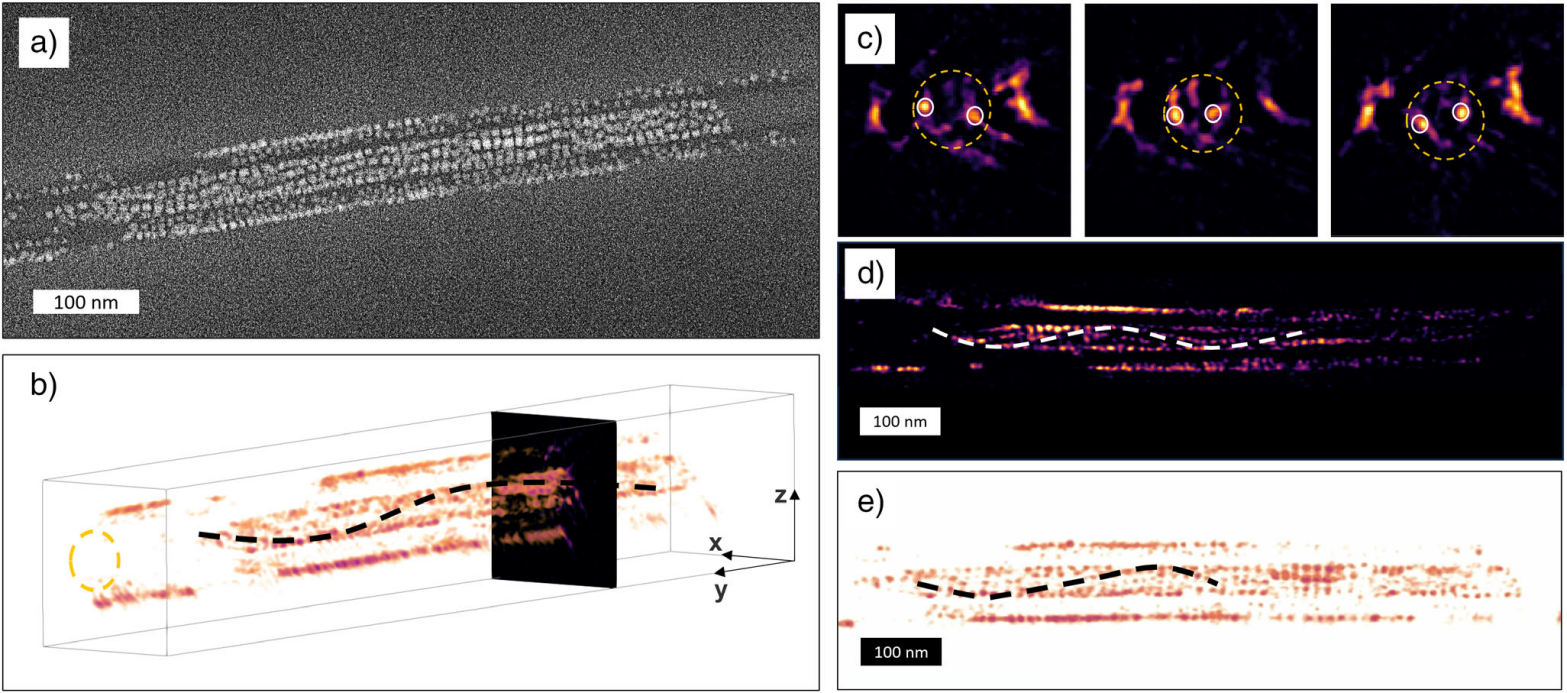
Electron tomography of helical assemblies of red‐NCs. a) High‐Angle Annular Dark‐Field ‐ Scanning Transmission Electron Microscopy (HAADF‐STEM) image of the region of interest in the sample, showing a single AZO nanotube with two sets of perovskite nanocrystals: (i) those deposited in the inner void and (ii) on the outer part of the AZO nanotube. b) Visualized tomogram of the area in a); a black dashed line highlights a single helical path of nanocrystals. c) Three tomographic slices taken along the y direction around the reference plane highlighted in b); the orange dashed line circumferences represent the diameter of the inner AZO nanotube void, onto which nanocrystals are deposited. The small white circumferences represent the location of the nanocrystals in consecutive slices. Rotation of the paired white circumferences across three slices confirms the helical arrangement of nanocrystals within the nanotube. d) Representative slice through the tomographic reconstruction taken along the x direction; the white dashed line indicates the helical arrangement of the nanocrystals in the inner void of the AZO nanotube. e) Tomogram view along the x‐direction, with a black dashed line indicating the helical arrangement of the nanocrystals in the inner void of the AZO nanotube.

To gain further insight into the origin of helicity in the samples, we performed electron tomography on a representative self‐assembled red‐NC sample, allowing us to more precisely investigate the 3D distribution of the particles on and inside a single AZO nanotube (Figures 5, and S26–S30, Movies S7 and S8). We measured the distance between the nearest nanoparticles in the helix from the tomogram. The surface‐to‐surface gaps for the three nearest‐neighbor pairs are 1.2, 1.5, and 2.1 nm, which fall within a range where the typical dipole–dipole interaction, which decreases with the third power of the distance, remains effective. This has been previously demonstrated for both plasmonic and perovskite nanocrystals. These separations also lie within the FRET distance window for CsPbX_3_ systems, for which the typical Förster radius is ∼10 nm.

With the morphological characterization in mind, we performed chiroptical measurements of the thin film composite nanomaterials. To elucidate artifact‐free CD spectra, we performed Mueller matrix measurements for an AZO film doped with 80 µL of blue‐NPLs at the B23 beamline of the Diamond Light Source synchrotron facility (Figures S31–S33). Using the differential matrix method,^[^ ^33^ ^]^ we calculated elementary polarization properties of the samples, that is CD and circular birefringence (CB), linear dichroism (LD, LD’) and linear birefringence (LB, LB’), where (’) relates to properties in samples rotated by 45° with respect to reference frame (see Supporting Information Note 5). The CD and CB spectra of the examined films were dominated by bands from AZO NTs, further confirming that the presence of PNCs does not distort the NTs structure. CD and CB had positive values for P domains and negative values for M domains. The high values of linear dichroism and birefringence may be explained by the presence of anisotropic aggregates of PNCs embedded in the AZO matrix. However, LD, LD’ and LB, LB’ did not exceed 1 deg, an order of magnitude lower than the CD signal. The CD band showed a dip at the absorption peak of blue‐NPLs, which can be attributed to increased unpolarized absorption by NPLs, thereby reducing the degree of circular polarization in transmitted light. Specifically, at the absorbance peak ∼460 nm, which corresponds to an exciton confined perpendicular to the surface of the NPL, the absorbance reaches a value of 2. This high absorbance results in significant attenuation of the transmitted light, thereby introducing substantial depolarization of the Mueller matrix, making it non‐physical in this range and leading to the observed apparent decrease in the CD signal.

**Figure 6:**
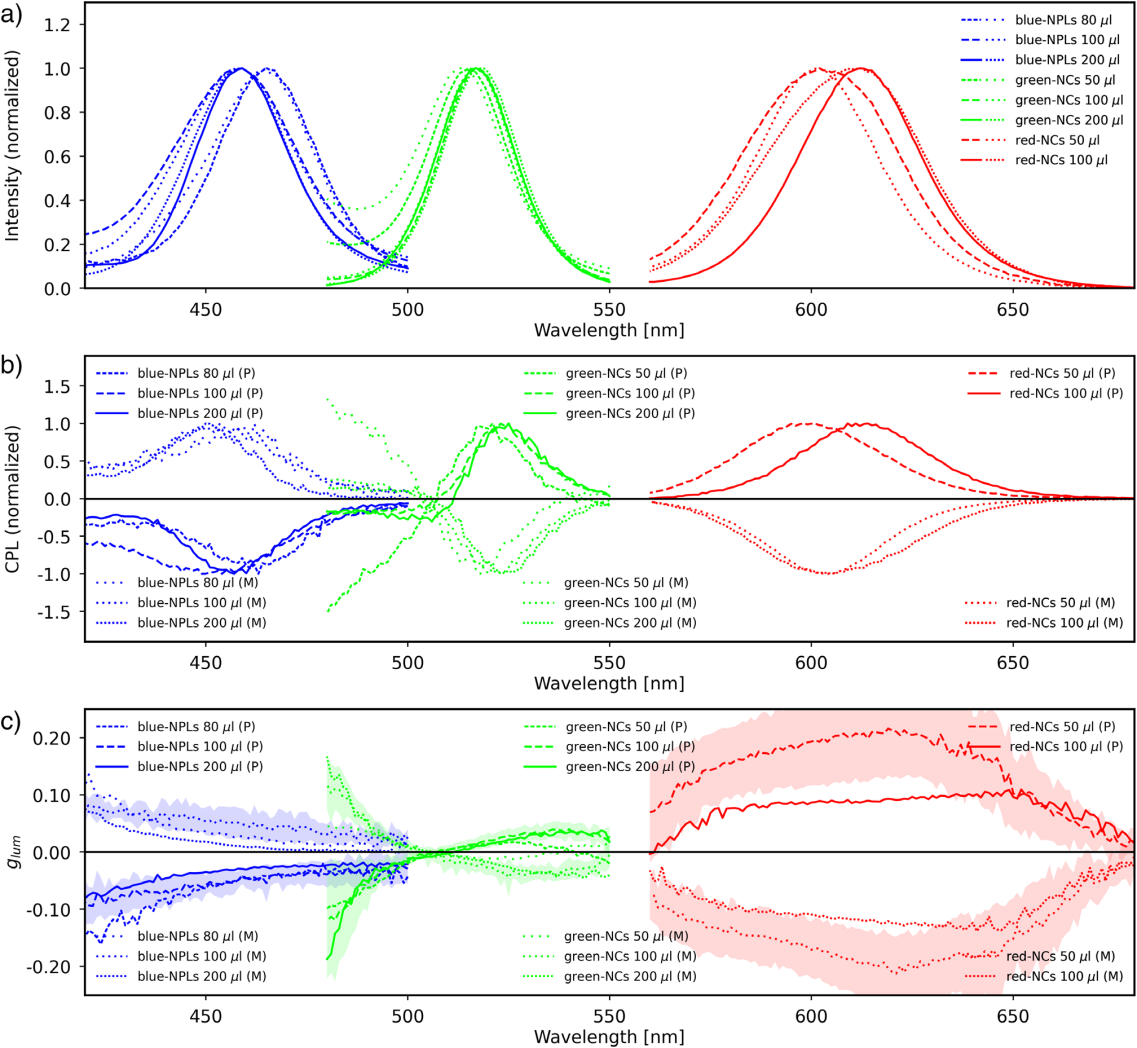
CPL spectra of AZO films doped with various amounts of PNCs. a) Emission spectra, b) normalized CPL spectra, and c) dissymmetry factors (*g_lum_*). Note that the results for blue‐, green‐, and red‐NCs are combined into a single graph, even though they were measured from independent samples, aiming to highlight the visible spectra range coverage and to facilitate comparison of the CPL sign in a given type of domain handedness across the spectral range. Solid and dashed lines represent data measured from P domains, dotted lines represent data from M domains. The shaded area in the *g_lum_* graph represents the standard deviation of the values measured over eight measurement spots; similar graphs for other PNCs concentrations and for photoluminescence and CPL are provided in Figure S37.

Non‐polarized PL emission spectra of PNCs embedded in the chiral matrix (Figures 6a and S34, Supplementary information Note 6) were similar to those of pristine, dried PNCs, albeit with slightly shifted emission maxima. The recorded emission bands were centered in the ranges of 455–465 nm and 512–519 nm, for blue‐NPLs and green‐NCs, respectively (Figure 6a). Thus, we note that for blue‐NPLs and green‐NCs, the mean emission maxima were almost the same as those in pure dried nanocrystals, but with variations from spot to spot. This distribution of emission maxima can be attributed to the presence of inequivalent nanosized spaces that can accommodate PNCs in different surroundings, as well as stochastically different PNC arrangements. This may lead to observing a blueshift (lower effective permittivity due to the presence of hollow spaces inside and around nanotubes) or redshift (a higher degree of aggregation of PNCs) when probing different sample spots. For red‐NCs, the emission was recorded within 603–613 nm. Therefore a larger difference between emission bands was observed in composite and dried nanocrystals, than for the other types of PNCs. This can be explained by the I^−^ anion exchange for Br^−^ traces that could be present in the red‐NCs solution used for thin film preparation.^[^ ^34^, ^35^ ^]^ We should note that the emission spectra remained reproducible within the ranges specified above.

Next, we performed CPL measurements from macroscopic areas of the homochiral domains. All individual measurements and those averaged over five orientations (see Materials and methods in Supporting Information), revealed significant circular polarization of the emitted light (Figures 6, and S35). The CPL bands of blue‐NPLs and red‐NCs showed maxima close to the corresponding emission band maxima, +/‐2 nm. For green‐NCs, the difference was +/‐20 nm, due to the bisignate character of spectra, with sign reversal at the rising slope of the emission band. It is worth noting that, due to the excitation beamline size, which is ∼0.5 × 5 mm^2^, the results were averaged over PNCs in various local chiral environments. Furthermore, for each sample where the homochiral domain size was larger than the size of the spectrometer beam, the measurements were repeated in eight independent spots within the domain, which verified the reproducibility of the results for samples that show the highest *g_lum_* values with uncertainty limited by spot‐to‐spot fluctuations of PNC concentrations. To more comprehensively compare the samples, we calculated the photoluminescence dissymmetry factors, defined as *g_lum _= *2*(I*_R_ *‐I*_L_)/*(I*_L_ *+ I*_R_), where *I*_L_ and *I*_R_ are left‐ and right‐handed circularly polarized light intensities averaged over all spots measured in each domain. For blue‐NPLs, *g_lum_* spectra did not show a maximum and monotonically decreased in the measurement range, starting from 0.18 at 420 nm (Figure 6c). For green‐NCs and red‐NCs, the dissymmetry factors reached 0.05 at 540 nm and 0.24 at 620 nm, respectively. It is worth highlighting that these are state‐of‐the‐art dissymmetry levels, higher than those typically reported for methods of chirality induction in PNCs based on chiral ligands,^[^ ^12^, ^13^, ^14^, ^16^, ^17^ ^]^ chiral assembly,^[^ ^15^ ^]^ chiral shape,^[^ ^36^ ^]^ and chiral cations.^[^ ^15^, ^37^, ^38^, ^39^, ^40^ ^]^

To fully appreciate the results and understand the origin of the high dissymmetry, we analyze the handedness of the CPL emission (Figure 6). We focus first on the CPL from blue‐NPLs, which exhibit right‐handed CPL for P domains (with positive CD), whereas the reverse is observed for M domains (Figure 6b,c). These results indicate that, in the NPLs emission range, the matrix acts as a filter, selectively absorbing one particular circular polarization handedness from the PL emitted by the PNCs. Consequently, light with the opposite handedness is preferentially transmitted. Indeed, the LC matrix exhibits strong circular dichroism in this spectral region. The shape of the *g_lum_* spectra supports this interpretation‐the absolute CPL dissymmetry values are largest at shorter wavelengths and decay as the wavelength increases. This aligns with the CD of the matrix, which exhibits higher absorption dissymmetry at shorter wavelengths.

For green‐NCs, the CPL signal has a different form, resembling a Cotton effect. However, the wavelengths at which the spectra change sign do not match the emission maximum, suggesting that the shape of the *g_lum_* spectrum is not truly bisignate. Notably, at shorter wavelengths (below ∼505 nm), the handedness of emission is the same as in the blue‐NPLs, suggesting that the filtering effect of the optically active matrix dominates. Above ∼505 nm, we observe a flip in the CPL sign, which means that mechanisms other than filtering take over. The “flipped” CPL handedness holds for red‐NCs‐their *g_lum_* has a reversed sign compared to a domain doped with blue‐NPLs. Notably, the higher dissymmetry value of the red emission is attributed to the weak spectral overlap with the AZO CD band, which suppresses filtering, allowing the intrinsic contribution to dominate.

The magnitude of *g*_*filter*_ closely follows the CD absorption profile of the matrix. Namely, for the M handedness domain, *g*_*filter*_ values are highest at shorter wavelengths and decrease toward longer wavelengths: 0.11, 0.09, and 0.01 at 435, 495, and 590 nm respectively. In contrast, for the same handedness of the domain, *g*_int_ values are negative in all cases: −0.08, −0.11, and −0.24, at the corresponding wavelengths. Given that *g*_*lum*_ = *g*_*filter*_ + *g*_*int*_, red‐NCs show the strongest intrinsic dissymmetry (0.25–0.01). These trends highlight a clear spectral dependence of the dominant CPL generation mechanism: selective filtering dominates in the blue, whereas intrinsic CPL prevails in the red.

**Figure 7:**
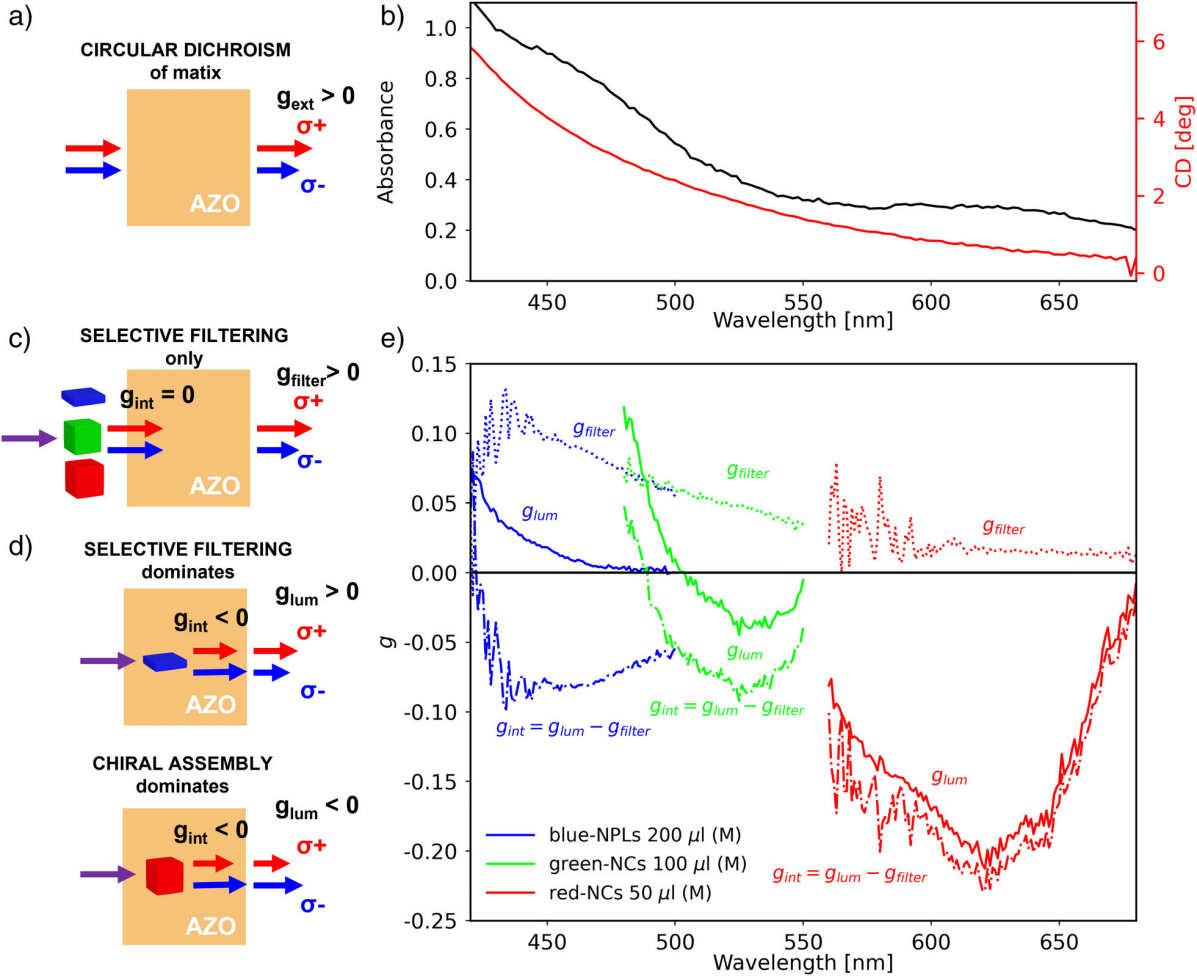
Disentangling intrinsic and reabsorption contributions in CPL of AZO films doped with PNCs. a) scheme showing the geometry for the measurement of circular dichroism of the AZO matrix, b) CD and absorption spectra of the M domain of pure AZO matrix, c) scheme showing the measurement geometry for pure selective filtering component from stacked PNCs and AZO films; d) scheme showing the CPL measurement of an ordinary sample showing both intrinsic CPL and selective filtering effect, where selective filtering dominates (blue‐NPLs, upper scheme) and intrinsic CPL dominates (red‐NCs, lower scheme); e) measured dissymmetry factors of ordinary samples (*g_lum_*) and reabsorption components (*g_filter_*), and reabsorption‐free *g_int_* spectra calculated using formula (1) for films showing the highest dissymmetry for each type of PNCs and measured on the M domain of AZO nanotubes. Data for P domains of AZO are presented in Figure S38.

Based on the above‐discussed results, we conclude that several effects influence the observed CPL, which are related to chiral assembly of PNCs and chiral reabsorption of emitted light by the template (Figure 7d). Below ∼505 nm, the *filtering effect* is dominant (Figure 7d, upper scheme), whereas at longer wavelengths the CPL is dominated by the effect of chiral assembly of PNCs and coupling between chiral partially ordered nanostructures composed of chiral nanotubes with the emission of perovskites (Figure 6d, lower scheme). To further support our conclusions, we prepared and measured samples of two 10 µm thick separate PNCs and AZO matrix films, stacked one on another (Supporting Information Note 7, Figure 7c, *g_filter_* at Figure 7e). PNCs are not in direct contact with the chiral matrix in this configuration. Thus, only non‐local effects caused by the selective reabsorption of light emitted from the layer of PNCs by the matrix is possible (Figure S36). As expected, the sign of CPL measured in this configuration was the same for the same handedness of domains for all samples, and both the bisignate spectrum for green‐NCs and opposite sign for red‐NCs were not observed. Accordingly, the highest *g_lum_* values were observed for blue‐NPLs, and decreased at higher wavelengths. This result is consistent with the CD of the AZO film, which decreases with increasing wavelength (Figures 7a,b and S1).

The reabsorption effect caused by the AZO matrix is expected to increase with the thickness of the sample. To investigate this, we performed CPL measurements on blue‐NPL and red‐NC samples with the doping level that gave the highest g*_lum_*, varying the film thickness (Supporting Information Note 8, Figures S39–S41). Consistent with our model, red‐NCs did not show statistically significant differences, whereas for blue‐NPLs, *g*_*lum*_ value increased seven‐fold between 4.5 and 11 µm thick samples (Table S1).

Further, we aimed to quantify the reabsorption (which leads to the chiral filter effect in CPL spectra), distinguishing it from other mechanisms that collectively contribute with an opposite sign to the filter effect. In the general case, the reabsorption contribution discussed above mixes with other mechanisms in a complex manner. However, as we demonstrate in Supporting Information Note 7, under reasonable assumptions and with not‐too‐large dissymmetries, the *g_lum_* can be expressed as the simple sum of the intrinsic dissymmetry factor (*g_int_*, i.e., the dissymmetry that the material would exhibit if the matrix were not absorbing in the visible range) and the dissymmetry factor resulting solely from reabsorption (*g_filter_*, i.e., the dissymmetry that the material would exhibit if light at the moment of emission from PNCs were achiral and became circularly polarized in the path through a chiral reabsorbing matrix). The latter can be directly measured when probing the CPL of stacked films of pure PNCs and a homochiral domain of a pure AZO film of the same thickness as the ordinary samples. The general formula that connects both dissymmetry components is given by:

$$g_{int}=4\frac{g_{lum}-g_{filter}}{4-g_{lum}g_{filter}}\approx g_{lum}-g_{filter}$$

where the last equality is valid only for small dissymmetries (|*g_lum_g_filter_* | ≪ 4).

Using this equation, we calculated the intrinsic dissymmetry factors, free of the reabsorption component, *g_int_* (Figure 7e), based on data from stacked films (Figure 7c,e) and the total dissymmetry of the ordinary PNC‐doped AZO film (Figure 6c). The obtained *g_int_* spectra indeed exhibited the typical shape of CPL‐active materials. Specifically, they showed a flat profile across almost the entire emission range, with maxima corresponding to the emission band maxima. Furthermore, the sign of the *g_int_* spectra remained consistent, regardless of the type of PNCs doped. We note that for blue‐NPLs and green‐NCs, at the short‐wavelength tail of emission, *g_int_* showed sign reversal, but in these terminal parts of the spectral range, the uncertainty in dissymmetry measurements is high due to a poor emission intensity. Only for red‐NCs *g_int_* values exceed 0.1 suggesting that the chemical composition of the perovskite is a more significant determinant of the *g*_*lum*_ values than the shape of the PNCs (Supporting Information Note 9).

Regarding the effect of PNC concentration, we observed that the dissymmetry of emitted light changed with the varying amounts of PNCs. Samples exhibited maxima for 50, 100, and 100 µL, for red‐NCs, green‐NCs, and blue‐NPLs, respectively. The weakest dependence of *g_lum_* on PNC concentration was observed for blue‐NPLs and for green‐NCs at wavelengths below the threshold, which is also consistent with the proposed mechanism of CPL generation. Indeed, if CPL is based on light filtering by the matrix, this mechanism does not depend on the local surroundings or the distribution of PNCs in the matrix. For red‐NCs, *g_lum_* decreased with increasing concentration, indicating that more PNCs precipitate as achiral aggregates within the matrix, which leads to an increase of the emission intensity but not of the chiral component. For green‐NCs, the dependence of *g_lum_* on concentration was similar to that of red‐NCs. However, due to their bright emission, CPL could be detected even from samples with sub‐optimal concentrations.

Finally, we rationalize the recorded *g_lum_* values in our nanomaterials. The process of CPL generation can be explained as a result of two fundamentally different effects: 1) *chiral assembly*‐dipole–dipole electromagnetic interactions between achiral PNCs assembled into chiral nanostructures, and 2) *selective filtering*‐chiral absorption by the matrix (chiral organic AZO nanostructures) resulting in preferential transmission of light with one handedness. The relative contributions of each effect vary with the selected spectral range, so that each PNC type yields a different CPL. For blue‐NPLs, whose emission band overlaps with the absorption band of AZO, the selective absorption process dominates, resulting in emitted light with opposite‐handedness compared to that preferentially absorbed by the matrix. At longer wavelengths, this effect decreases because the absorption by the matrix decreases, whereas contributions of the local chiral assembly of PNCs increase. The CPL sign from the latter mechanism is opposite to the one generated through the reabsorption effect (*selective filtering*). The emission range of green‐NCs lies in between the aforementioned regimes and therefore shows bisignate characteristics, with chiral reabsorption dominating below ∼505 nm and chiral assembly contributions above that threshold.

**Figure 8:**
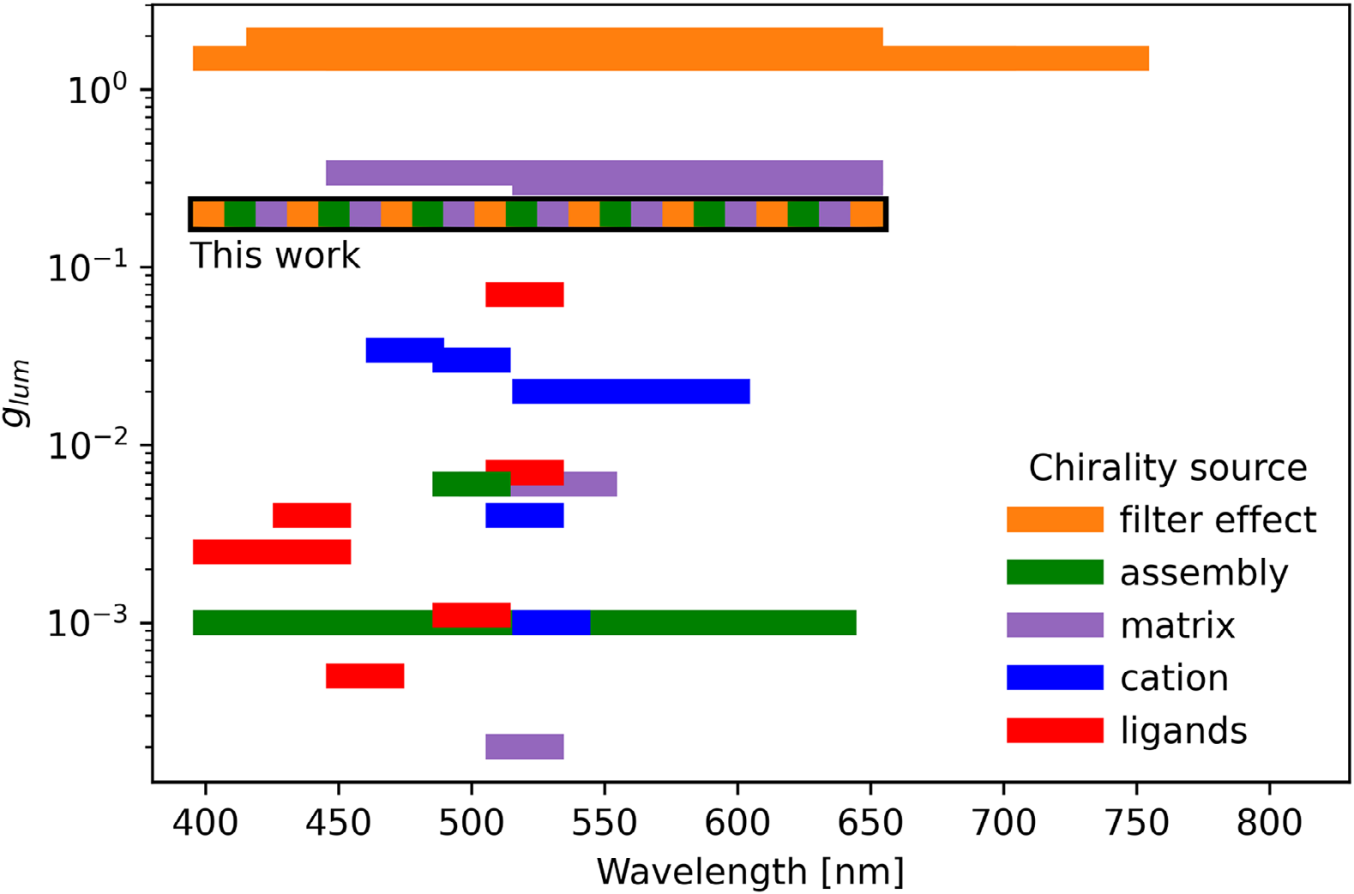
Comparison of CPL intensity, bandwidth, and generation mechanisms presented in this study, against other recently reported approaches^[^ ^12^, ^13^, ^14^, ^15^, ^16^, ^17^, ^20^, ^21^, ^25^, ^37^, ^38^, ^39^, ^40^, ^43^, ^44^, ^45^, ^46^, ^47^, ^48^, ^49^ ^]^ (Supporting Information Note 10, and Table S2).

It is worth noting, that a few other mechanisms may also be considered in the examined films, e.g. interactions through chiral electromagnetic fields with the chiral environment around PNCs induced by chiral nanotubes. As shown theoretically and experimentally, near‐field electromagnetic interaction of nanocrystals with organic components typically does not lead to such high dissymmetry of emitted light as presented here.^[^ ^41^, ^42^ ^]^ We therefore conclude that, although this effect may be present in the examined samples, it does not determine such a strong CPL response. Further, the PNCs confined within the nanotube network could be potentially influenced by non‐local effects resulting from the chiral photonic structure formed by the short‐order assembled nanotubes. However, our structural examination did not provide evidence for the order required for photonic bandgap properties to arise.^[^ ^25^, ^43^, ^44^ ^]^ (Figure 8). As each of these effects depends on different factors, the presented strategy may enable the fabrication of CPL‐active materials with tailored properties, even by tuning a single parameter of the structure, as exemplified when comparing chiroptical properties in different spectral ranges.

## Conclusion

In conclusion, we demonstrate CPL with an unusually high dissymmetry from PNCs embedded in a multiscale chiral matrix. By managing the interplay between intrinsic chiral assembly in a chiral environment and the filtering effect of the matrix, we achieved *g_lum_* values reaching ≈ 0.24. By changing the shape and anion composition of PNCs, we could tune the color of emitted light to cover almost the entire visible spectrum. The fabrication process of these materials does not require any chemical modifications on the surface of PNCs, which are fabricated using well‐established protocols.

Our results thus represent a step forward toward the fabrication of efficient CPL‐active materials. The solubility in organic solvents and the low melting point of the matrix facilitate processability, allowing integration with existing fabrication methods for emissive thin films, which are essential components for circularly polarized displays, solar cells, and optoelectronic detectors. Moreover, by decomposing the *g*_*lum*_ into two contributions (*g*_*int*_ and *g*_*filter*_), we clarify that the intrinsic, chiral assembly mechanism dominates the red and (part of) the green emission. In contrast, the *selective filtering* effect dominates in the blue emission. Based on the unique emissive properties of perovskites, high emission efficiency of PNCs, and high dissymmetry of CPL, we propose that the presented approach can be expanded to a broader range of emitters, including other types of PNCs, quantum dots, and/or achiral fluorescent dyes, and become a universal pathway for CPL generation in the solid state.

## Supporting Information

The authors have cited additional references within the Supporting Information [50–64].

## Conflict of Interests

The authors declare no conflict of interest.

## Supporting information



Supporting Information

Supporting Information

## Data Availability

The data that support the findings of this study are available from the corresponding author upon reasonable request.
