# Supplementary material for: The Combination of Chiral Assembly and Chiral Template Effects Boosts Circularly Polarized Luminescence of Perovskite Nanocrystals
Source: Angew Chem Int Ed Engl. 2025 Nov 22;65(1):e07812. doi: 10.1002/anie.202507812 (PMC12759238; doi:10.1002/anie.202507812)
Supplement: Supplementary file 1 — Supporting Information [file ANIE-65-e07812-s002.pdf]

Supporting Information for

The Combination of Chiral Assembly and Chiral Template Effects Boosts Circularly Polarized Luminescence of Perovskite Nanocrystals

Mateusz Pawlak, Julia Abramowicz, Nadesh Fiuza Maneiro, Gail A. Vinnacombe-Willson, Sunghwan Jo, Elie Benchimol, Piotr Roszkowski, Zitao Chen, Da Wang, Guido H. Clever, Luis M. Liz-Marzán, Agustín Mihi, Lakshminarayana Polavarapu and Wiktor Lewandowski*

Materials and methods.

Materials: Cesium carbonate (Cs_2CO_3, 99.9%), lead (II) bromide ($PbBr_2$, >98%), lead (II) iodide (PbI_2, 99%), 1-octadecene ($C_{18}H_{36}$, 90%), oleic acid ($C_{18}H_{34}O_2$, 90%), and oleylamine ($C_{18}H_{37}N$, 70%) were purchased from Merck. Toluene solutions were purchased from Merck.

AZO was synthesized using a previously published protocol. [50] starting from 1-bromododecane, benzocaine, phenol, and 1,5-pentanediol. All reagents were of 97% purity or higher and purchased from Merck.

All chemicals were used as received.

Preparation of precursors solutions:

Preparation of cesium oleate precursor solution (CsOL). The cesium precursor solution was prepared by dissolving 407.0 mg of cesium(I) carbonate (1.25 mmol) in 20 mL of octadecene, along with 1.25 mL of oleic acid, under vigorous stirring at 130 °C. The reaction between the carbonate and the acid produces a white precipitate, which dissolves only at temperatures exceeding 120 °C. Therefore, the precursor must be heated prior to use.

Preparation of the PbI_2 Solution: 1 mmol of PbI_2, 1.5 mL of oleylamine, 1.5 mL of oleic acid, and 100 mL of toluene were loaded in a 250 mL Pyrex bottle. The mixture was heated to 60 °C under vigorous stirring until the PbI_2 completely dissolved. The resulting PbI_2 solution in toluene was used as the iodide source for synthesizing mixed-halide perovskite nanocrystals (NCs).

$CsPbBr_3$ Perovskite nanocrystals synthesis:

$CsPbBr_3$ nanocubes:

Nanoparticles were synthesized using the hot-injection method developed by L. Protesescu et al. Initially, the lead precursor solution was prepared by dissolving 58 mg of lead(II) bromide (0.20 mmol) in 5 mL of octadecene, along with 0.5 mL each of oleylamine and oleic acid as ligands, under vigorous stirring at 125°C. Once fully dissolved, the precursor solution was heated to 175°C, and 0.4 mL of pre-heated CsOL precursor (prepared as described earlier) was rapidly injected under vigorous stirring. After 3–5 seconds, the reaction was quenched by placing the solution in an ice-water bath.

The synthesized $CsPbBr_3$ nanocubes were purified using a two-step procedure. First, the solution was centrifuged at 10,000 rpm for 10 minutes and redispersed in 3 mL of toluene. Then, it was centrifuged at 4,000 rpm for 10 minutes to precipitate secondary bulk material, while the supernatant containing the nanocrystals was retained.

$CsPbBr_3$ nanoplatelets:

To synthesize nanoplatelets, a protocol similar to the one previously described was followed, with the solution temperature reduced to 50°C during the hot injection of CsOL. The purification process was the same as that used for $CsPbBr_3$ nanocubes, with one key modification: 14 mL of acetone was added to the as-prepared colloidal dispersion to precipitate the $CsPbBr_3$ nanoplatelets. The resulting dispersion was then purified via centrifugation at 10,000 rpm for 10 minutes, after which the pellet was re-dispersed in 5 mL of toluene. Finally, the colloidal dispersion underwent a second centrifugation at 3,000 rpm for 10 minutes to remove larger particles.

NCs by Halide Exchange Using PbI_2 Solution.

Synthesis of $CsPbBr_1I_2$ Perovskite NCs: $CsPbBr_1I_2$ nanocrystals (NCs) were synthesized through a halide exchange reaction. In a typical procedure, 500 µL of the previously synthesized $CsPbBr_3$ NC colloidal dispersion was transferred into a 25 mL glass vial. The PbI_2 solution was then

gradually added under vigorous stirring until the photoluminescence (PL) emission shifted to the desired wavelength. The progress of the reaction was monitored via PL spectroscopy, with the observed red-shift in PL emission confirming the successful halide exchange process.

***Preparation of AZO/PNCs thin films*:**

16.8 mg of AZO was dissolved in 1 ml of toluene. Then, 200 µl of the resulting solution was transferred to a small glass vial. The appropriate volume of PNCs solution (Supplementary Note 1) was added and mixed. The entire mixture was drop-casted onto a glass coverslip and left at room temperature until the toluene evaporated. Subsequently, the glass slide with the solid residue was heated on a heating stage until melting at 115 °C. The molten film was then covered with another glass coverslip and pressed lightly to ensure uniform distribution of the material between the slides. Finally, the film was cooled down to room temperature at a rate of 2 K/min, yielding CPL-active samples containing large homochiral domains of AZO nanotubes doped with PNCs.

***CPL measurements*:**

CPL measurements were performed using a JASCO CPL-300 spectrometer with a xenon lamp as the excitation light source. The excitation wavelength was set to 350 nm and remained the same for each PNC type. The emission and excitation bandwidths were adjusted within the range of 13-15 nm to achieve optimal intensity on the detector (DC in the range of 0.02-0.07 V).

The sample was placed in the holder for solid samples in an orientation where the optical axis passed through the homochiral domain. If the measured domain was smaller than the beam spot size (~0.5 × 5 mm^2), the area outside the domain was masked using black, non-emissive tape.

To confirm that the measured signal was not an artifact caused by sample anisotropy, measurements were repeated five times with the sample rotated by 0°, 90°, 180°, and 270° around the optical axis and flipped. For uniformity assessment, samples were measured at eight different spots, averaged, and the standard deviation was calculated. Depending on signal intensity, each measurement was averaged over 2-10 scans**.** For all measurements, the Digital Integration Time was set to 0.5 s, and the data pitch was 1 nm.

Thickness-dependent CPL measurements for red-NCs were performed using a custom-made PEM-based CPL spectrometer setup (published at [51]) in 180° geometry. Data were averaged over 8 spots (~0.5 × 5 mm^2 each), isolated using black non-emissive tape, with one scan per spot at a 2 nm data pitch.

XRD measurements:

X-ray diffraction (XRD) measurements were performed on thin films containing drop-cast perovskite nanocrystals (without the AZO matrix). Heat-dependent measurements of $CsPb(Br/I)_3$ perovskite NCs measured on the a) fresh film, b) after one annealing step at 115 degrees and c) after two annealing cycles were performed using a Bruker AXS D8 diffractometer (Figure S11) ; the diffractograms of blue-NPLs and green-NCs were acquired at ambient temperature directly after drop-casting, using a PANalytical -XPERT-PRO diffractometer (Figure S12).

High-Angle Annular Dark-Field - Scanning Transmission Electron Microscopy (HAADF-STEM):

The high-angle annular dark-field scanning transmission electron microscope (HAADF-STEM) images were acquired using a Thermo Fisher Talos F200X microscope with an acceleration voltage of 200 kV. The tomographic tilt series was (49 projection images), with a tilt range from -70° to 70°, were measured using ADF-STEM. Tomography data preprocessing was performed in Matlab, using the center of mass and standard line methods to align the tilt series. Tomographic reconstructions were obtained using the Simultaneous Reconstruction Technique (SIRT).

Supplementary Notes.

Supplementary Note 1.

We conducted experiments and theoretical modeling to determine the size distribution of AZO homochiral domains and their dependence on thin-film heat annealing conditions.

<u>Experimental results (Figures S2-S8)</u>

The domain sizes were measured from samples prepared using the standard 18 mm × 18 mm glass slides, which were prepared in the same manner as the AZO/PNCs films used in the article. The size was assessed using polarizing optical microscopy, with automated analysis based on a Python-based image segmentation script. The script calculated the difference between images taken at 85° and 95° angles between the analyzer and polarizer and assigned (P) domains to areas where the difference was above a given threshold, and (M) domains to areas where the values were below the threshold. It then calculated the distribution of the domain areas. To avoid errors introduced by empty regions (filled by air) of the samples, such areas were manually masked before image analysis.

At a cooling rate of 2 °C/min that was used in the original remelting procedure, a typical sample contained one to three homochiral domains with a lateral size of approximately 1 cm. However, the average domain area was 0.42 ± 0.58 cm², with the average size strongly influenced by the number of smaller domains formed at the edges of the samples. These measurements were performed on 20 samples to achieve a relatively low distribution of sizes.

When the cooling rate was increased, the domain size decreased. Namely, at 10 °C/min and 20 °C/min, the domain areas were 0.055 ± 0.152 cm² and 0.032 ± 0.104 cm², respectively. For these two cooling rates, the domains were significantly smaller than in the 2 °C/min case; thus, already four samples were enough to obtain a stable distribution of domain sizes.

Beyond the area and standard deviation, we also include domain size distribution charts, which, on the one hand, visualize the presence of large domains, while on the other hand, are helpful for the theoretical analysis of domain growth.

Overall, these results confirm that slower cooling favors fewer, larger domains, whereas rapid cooling yields smaller, numerous ones.

<u>Modelling of the domain growth (Figure S9-S10)</u>

To clarify how cooling rate governs domain formation and understand the distribution of domain sizes, we developed a simple model of domain formation, based on the following assumptions:

1) the simulation begins with a square liquid sample,
2) new crystallization points appear at random moments and locations, adopting random handedness,
3) domains originating from each crystallization point grow radially while preserving the handedness of their point of origin,
4) once the chirality of any point in the sample is determined, it cannot be altered.

The process was simulated using a Python script that implemented the following pseudocode, where the inputs are the probability of forming a new crystallization point per timestep (p) and the domain growth rate, defined as a fraction of the sample side length per timestep (r). Simulation

was repeated 50 times to obtain the expected theoretical distribution of domain sizes. The only external parameter required for the simulation is the ratio of the probability of new crystallization point formation per timestep to the domain growth rate (p/r). By varying this ratio, we can mimic different cooling conditions. At a high ratio, new domains appear more frequently compared to existing ones that are growing, simulating high cooling rates. Conversely, at a low ratio, new domains appear less often, leading to the formation of smaller domains and mimicking conditions at slow cooling rates.

By repeating the calculations for varying p/r ratios, we found conditions that reproduce the domain size distribution with means and standard deviations matching the distributions obtained experimentally. Namely, the ratios that matched the experimental values for cooling rates of 2, 10, and 20 °C/min were 100, 15,000, and 50,000, respectively.

The used algorithm:

1) Generate a 100x100 mesh representing the sample with an attributed liquid state (0)
2) Repeat the following procedure (timestep) as long as no liquid pixels remain:
   a) For each existing crystallization point, increment its radius R by 100r
   b) For each pixel within distance R of any crystallization point and still in liquid state (0), attribute the chirality of the respective crystallization point
   c) Draw a random number between 0 and 1
   d) if it is smaller than p, create a new crystallization point:
      -draw the random pixel coordinates on the mesh (x,y)
      -draw the random number between 0 and 1, if it is above 0.5, attribute (+1) chirality to pixel (x,y), else attribute (-1) chirality
   d) return to step 2a)
3) After complete crystallization, calculate the distribution of domain sizes.

Supplementary Note 2.

We obtained the XRD of the $CsPb(Br/I)_3$ NC films before and after two annealing cycles (Figure S11). The XRD pattern remains unchanged after annealing, suggesting that the structure remains unchanged. As shown in the XRD patterns in the graph below, the diffraction peaks remain constant, with the only noticeable change being a slight increase in the intensity of the peak at 25.3° after the annealing cycles. However, this peak is not associated with degradation, particularly in the absence of other characteristic signals of degradation, such as the PbI_2 peak at 12.6°, the $CsPb_2Br_5$ peaks near 11.7° and 13.1°, or the Cs_4PbBr_6 peaks near 12.9° and 21.5°. As no additional degradation-related peaks appear and the 25.3° peak remains stable after annealing, the structure remains unchanged.

Additionally, the NCs remain strongly luminescent under UV light, indicating that the annealing process does not significantly impact the luminescence efficiency.

.

Supplementary Note 3.

The amount of PNCs doped into the AZO matrix was controlled based on UV-vis absorption spectroscopy. The procedure for obtaining the PNC solution was as follows:

1) For green-NCs and red-NCs, the solution was prepared to achieve an absorbance of 1.25 at 400 nm. First, a base solution of NCs was obtained by precipitating the NCs by centrifugation and then dispersing them in approximately 400 µl of toluene. The absorbance of the base solution was determined by measuring 30 µl of the solution diluted 10 times in a 1 cm thick quartz cuvette ($\boldsymbol{A_{measured}}$). The volume of toluene added to 200 µl of the 'base solution', in order to obtain the final solution for doping, was calculated using the following formula:

$$\boldsymbol{V_{toluene}} = \left(\frac{\mathbf{1.25/10}}{\boldsymbol{A_{measured}}} - \mathbf{1}\right) \cdot \mathbf{200}\ \boldsymbol{\mu l}$$

2) For blue-NPs, the procedure was analogous, but the target solution absorbance was 1.76 at the maximum absorption peak (at ~440 nm; position of the peak depends on the thickness of the NPls). The volume of toluene added to the base solution was calculated using the formula:

$$\boldsymbol{V_{toluene}} = \left(\frac{\mathbf{1.76/10}}{\boldsymbol{A_{measured}}} - \mathbf{1}\right) \cdot \mathbf{200}\ \boldsymbol{\mu l}$$

As prepared solutions of PNCs were added to 1.68 mg of AZO before drop-casting.

Supplementary Note 4.

To represent the nature of the assemblies in thick samples, we employed different sample preparation methods for SEM and TEM. Their detailed description, including advantages and limitations, is provided below. They include (#1) SEM imaging of cracked thin films and (#2) scraping a small amount of the thin film onto a TEM grid, both of which rely on a thin film similar to the one used for optical measurements. Also, we used TEM imaging of thinner samples prepared by (#3) scraping the films, sonicating the material, and drop-casting to the TEM grid, (#4) evaporation of PNCs and AZO solution on the TEM grid, (#5) using the previous procedure but complemented with remelting the material. In the main text, we present results achieved using method #1 (Figure 4a, b, e, and f), #2 (Figures 3a and 4c, d, and g-i), and #3 (Figures 3b-h and 4j-l). Additional images, including those from other measurement types, are shown in Supplementary Figures S17-S25.

1) SEM of thick films (Figure S23)

The samples were prepared in the same manner as for optical measurements, with the exception that one of the substrates was silicon. In detail, the sample was prepared by drop-casting a mixed toluene solution containing 1.68 mg of AZO and either 80 µL of the NPLs solution or 200 µL of the NCs solution, as described in Supplementary Note 1, onto a silicon substrate. After solvent evaporation (at ambient conditions), the sample was heated to 115 °C, covered with a glass slide, and cooled at a 2 K/min rate. Once the sample reached room temperature, the glass slide was

removed, and the sample was placed in the SEM chamber. Only a small portion of the material was removed with the glass slide.

Advantages: This method enabled us to observe the distribution of PNCs near the surface that had been in contact with the glass slide during sample remelting, as well as the internal structure within cracks or along the edges of the film. Since we are dealing with a thin film, this method should represent the distribution of particles within the organic material (see the limitations below).

Limitations: A significant limitation was the resolution of the SEM imaging compared to TEM techniques. Even when using a state-of-the-art SEM machine, observing individual PNCs is at the limit of the technique. PNCs inside the organic nanotubes are less visible; thus, we expect an underrepresentation of their population. Cracks may preferentially form in defects; thus, we expect an overrepresentation of larger aggregates of PNCs or defective nanotubes.

2) TEM measurements of samples after ultrasound treatment (Figure S24)

The samples were first prepared for the optical measurements. Then, the material was scraped into a small glass vial. The scraps (~0.5 mg) were sonicated in 1 mL of hexane for 1 minute. Since hexane does not dissolve AZO molecules, it preserves the integrity of the nanotubes. 5 µl of the resulting dispersion was drop-cast onto a copper TEM grid with a carbon film.

Advantages: This method allowed us to use TEM imaging of individual nanotubes. Therefore, it was optimal to observe PNCs trapped inside AZO nanotubes and assemblies of PNCs on the surface of the nanotubes.

Limitations: We can expect that sonication may affect the supramolecular structure of the film. Particularly, hexane may dissolve PNCs located outside (in between) nanotubes, which, during

solvent evaporation, may attach to the surface of PNCs; thus, we can expect that the arrangement of the outer PNCs may partially differ from that achieved during slow cooling of a thin film.

3) TEM measurements of samples which were scraped (Figure S25)

This method is similar to that above. The difference is that small scraps of the thin film were mechanically transferred onto the surface of the TEM grid and no other treatment was used.

Advantages: This approach enabled the use of TEM, and prevented structural changes caused by sonication expected in the previous method.

Limitations: Mechanical scraping partially destroyed the material, forming amorphous structures, which are not expected for a regular thin film. These amorphous regions were observed near nanotubes that kept integrity. Only relatively thin scraps were suitable for imaging.

4) TEM measurements of samples drop-casted onto the TEM grid and formed upon solvent evaporation (Figure S21)

In this method, mixtures of toluene solutions of AZO and PNCs were drop-cast onto a TEM grid, followed by solvent evaporation at room temperature. No further treatment was used.

Advantages: This approach provided samples with optimal thickness for TEM imaging, well dispersed over the area of the TEM grid.

Limitations: These structures formed under slightly different conditions than those in the thin film. Nanotubes formed during solvent evaporation at room temperature, not when crystallizing from the melted state. In these samples, we noted that almost all PNCs were in contact with nanotubes, possibly due to kinetic reasons, not fully representing the thermodynamic equilibrium, and overrepresenting PNCs interacting with nanotubes.

5) TEM measurements of samples drop-casted onto the TEM grid and formed upon heat annealing (Figure S17-S20 and S22)

This method is similar to that above, with the difference that the material was remelted after drop-casting. Namely, it was heated to 115 °C and cooled to room temperature at a 2 K/min rate, that is in the conditions in which thin films for optical measurements were prepared.

Advantages: This approach provided samples with optimal thickness for TEM imaging, prepared in a way that resembles that used for the thin films (heat annealing, often individual domains growing from a single crystallization point).

Limitations: During the remelting, we expected that the wetting the TEM mesh by organic material may result in regions of very thin layers of organic materials, which do not form nanotubes. The amount of material is significantly lower than in films for optical measurements; the available volume is larger than that in a thin molten film covered with a glass slide. Thus, we expected an overrepresentation of phase-separating PNCs, increasing the volume occupied by PNCs pushed out by the crystallizing nanotubes. Also, since a **larger** amount of PNCs may not be in direct contact with the organic material, we expect higher odds of PNC agglomeration in respect to the situation in which the material is in the form of a thin film.

Supplementary Note 5.

We used the differential Muller matrix method to separate the linear and circular optical contributions.

We calculated the natural logarithm of the raw Mueller matrix (M) measured at a synchrotron beamline. In a perfectly nondepolarizing medium, the resulting 4×4 differential Mueller matrix (m) is G-antisymmetric and contains six independent off-diagonal elements corresponding to CD, CB, LD, LD′, LB, and LB (m_{03} = m_{30} = CD, m_{12} = -m_{21} = CB, m_{01} = m_{10} = LD, m_{02} = m_{20} = LD’,m_{31} = -m_{13} = LB’, m_{23} = -m_{32} = LB). In strongly depolarizing media, cross-couplings arise between LD/LB/CD and the differential depolarizing terms. In this case, the differential Mueller matrix is decomposed into the sum of G-antisymmetric and G-symmetric components. The elementary optical properties extracted from the former are ensemble averages, whereas the latter encodes their variances.

For our samples, the matrix is almost perfectly G-antisymmetric, as can be judged based on the results shown in Figure S32. from which we can see that m_{03} = m_{30}, m_{12} = -m_{21}, m_{01} = m_{10}, m_{02} = m_{20}, m_{31} = -m_{13}, m_{23} = -m_{32}. This confirms the reliable decomposition of the optical phenomena. Only at shorter wavelengths (below 450 nm), due to the sample’s high absorption, some elements do not behave properly (i.e., $m_{21} \neq m_{12}$), but even there, the CD remains reliable as $m_{03} \approx m_{30}$.

Thus, we conclude that the CD spectra presented in the manuscript are free from LD contamination and that depolarization is negligible.

Supplementary Note 6.

To measure quantum yield for luminescence, we first focused on choosing a suitable method. Our samples consist of thin films placed between two glass slides, which differs from the regular dispersion-oriented measurements, and may influence the obtained results. A reasonable approach for measurements of such samples was introduced, e.g., by J. C. de Mello et al. (Adv. Mater., 1997, 9, 230). The proposed method requires measurements in three configurations: (a) without the presence of the sample in the sphere, (b) with the sample placed in the sphere but not in the direct path of the exciting beam, and (c) with the exciting beam directly hitting the sample. These spectra enable the calculation of areas over the exciting beam profile (L) and emission profile (P). We performed measurements on thin films of AZO doped with each perovskite nanocrystal type at the level that showed the highest luminescence values, following the procedure described by de Mello et al.

We should note that the method has an absolute error of approximately 2%, while the accuracy of the detector to which we had access is limited to the range above 650 nm, thereby reducing the reliability of the measurements:

- for AZO doped with green-NCs, the value was ≈2% (Figure S34); this is the most reliable result;
- for red-NCs, the emission band lies near the noise level of the detector; thus, the value of ≈7.5% is not fully reliable;
- for blue-NPLs, the emission was below the detection limit, due to the strong absorption of AZO in the range that is used for excitation.

Supplementary Note 7.
To estimate the relative contribution from reabsorption effects and other mechanisms described in the main text we compare: (i) the dissymetries of emission for samples in which PNCs were doped to AZO (Figure 4) with (ii) dissymetries measured for stacked films of pure PNCs and pure AZO (Supplementary Figure S36). We consider the model of the AZO and PNCs interaction where three mechanisms lead to intrinsic circularly polarized light directly from the PNCs, while reabsorption changes the circular polarization of light surpassing through the sample.

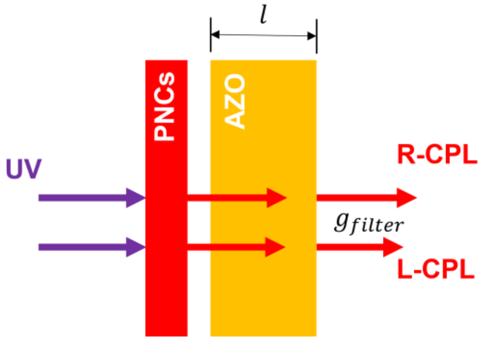

In a stack of samples, light emitted from the pure PNCs layer is initially unpolarized. The intensities of left- and right-handed circular polarizations from this layer are the same. However due to the differential absorbance of AZO film, the transmitted intensities (I^L_{reabs},I^R_{reabs}) become unequal and can be expressed as:

$$I^R_{reabs} = I_0 10^{-A^R_f} \quad (3)$$

$$I^L_{reabs} = I_0 10^{-A^L_f} \quad (4)$$

Where A^L_f and A^R_f are absorbances of left- and right-handed circularly polarized light in AZO film. Thus, the dissymmetry factor measured in this experiment g_{filter} can be calculated as:

$$g_{stack} = 2\frac{I^L_{reabs} - I^R_{reabs}}{I^L_{reabs} + I^R_{reabs}} = 2\frac{1 - 10^{A^L_f - A^R_f}}{1 + 10^{A^L_f - A^R_f}} \quad (5)$$

From this equation we can calculate coefficient that we will use in further analysis:

$$10^{A^L_f - A^R_f} = \frac{2 - g_{filter}}{2 + g_{filter}} \quad (6)$$

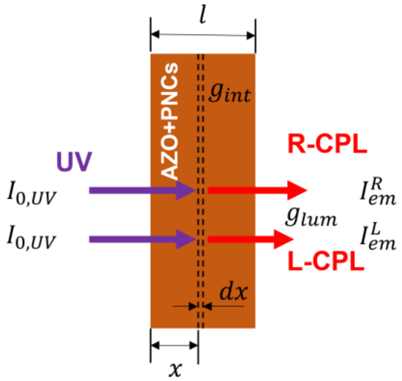

Another set of data we will use is the dissymmetry factor measured from single films containing AZO and PNCs (g_{lum}), that is, the regular films we focus on in this work. In this case, the reabsorption process overlaps with the three other CPL-induction mechanisms introduced in the main text. The resultant effect of these three mechanisms will be modeled using the effective coefficients φ^L nad φ^R, which relate the intensities of left- and right-handed circularly polarized light to the absorbed intensity of the excitation light. Furthermore, the reabsorption process is more complex to describe because light is emitted from the entire volume of the film, meaning the path length of the light through the sample depends on the location where it is emitted.

Let us consider infinitely thin element of film, thickness of which is dx and is at distance x from the surface of sample which faces towards UV light source. The intensity of UV light that comes on the depth x is given from Beer-Lambert law as:

$$I_{UV} = I_{0,UV} 10^{-A\frac{x}{l}} \tag{7}$$

Where $I_{0,UV}$ is the intensity of excitation light and l is the total thickness of the film.
Amount of light absorbed by element of thickness dx is thus given by:

$$dI_{abs} = I_{0,UV} 10^{-A\frac{x}{l}} dx \tag{8}$$

Intensity of light emitted from the same element is proportional to absorbed light intensity and differs for both handednesses of light:

$$dI_{em}^R = \varphi^R I_{0,UV} 10^{-A\frac{x}{l}} dx \tag{9}$$

$$dI_{em}^L = \varphi^L I_{0,UV} 10^{-A\frac{x}{l}} dx \tag{10}$$

Before reaching the detector, the emitted light must pass through the remaining thickness $l - x$ of the sample, during which it is reabsorbed by the matrix. Therefore, the intensities that reach the detector are given by:

$$dI_{em}^R = \varphi^R I_{0,UV} 10^{-A\frac{x}{l}} 10^{-A_f^R\frac{l-x}{l}} dx = \varphi^R I_{0,UV} 10^{-(A-A_f)\frac{x}{l}-A_f} dx \tag{11}$$

We present the equation above, as well as the subsequent equations for intensity, only for right-handed circularly polarized light. The formulas for left-handed circularly polarized light are identical, with L replacing R in the superscripts.

Integrating over the whole thickness of the film we can obtain the total intensity that reaches the detector as:

$$I_{em}^R = \varphi^R I_{0,UV} 10^{-A_f^R} \int_0^l 10^{-\left(A-A_f^R\right)\frac{x}{l}} dx = \frac{\varphi^R I_{0,UV} l}{\ln(10)} \frac{\left(10^{-A_f^R} - 10^{-A}\right)}{(A - A_f^R)} \tag{12}$$

The excitation light is strongly absorbed by the matrix and PNCs due to their high absorption in the UV range, which is an order of magnitude greater than in the visible range. Therefore, we can assume that $A \gg A_f^R$, allowing us to simplify the above equation by skipping smallest elements in last fraction:

$$I_{em}^{R} \approx \frac{\varphi^R I_{0,UV} l}{\ln(10)} \frac{10^{-A_f^R}}{A} \tag{13}$$

Finally, the dissymmetry factor measured in this case can be calculated as:

$$g_{lum} = 2\frac{I_{em}^L - I_{em}^R}{I_{em}^L + I_{em}^R} = 2\frac{\varphi^L 10^{-A_f^L} - \varphi^R 10^{-A_f^R}}{\varphi^L 10^{-A_f^L} + \varphi^R 10^{-A_f^R}} \tag{14}$$

On the other hand, if the sample did not exhibit reabsorption of the emitted light, the intensities could be calculated in the same way, but with $A_f^R = 0$, as follows:

$$I_{theor}^{R} = \frac{\varphi^R I_{0,UV} l}{\ln(10)} \frac{(1 - 10^{-A})}{A} \tag{15}$$

The theoretical intrinsic dissymmetry factor of CPL does not come from the reabsorption process but only three other processes that take place:

$$g_{int} = 2\frac{I_{theor}^L - I_{theor}^R}{I_{theor}^L + I_{theor}^R} = 2\frac{\varphi^L - \varphi^R}{\varphi^L + \varphi^R} \tag{16}$$

By combining equations (14) and (16) we obtain:

$$g_{int} = 2\frac{(g_{lum} + 2) - 10^{A_f^R - A_f^L}(2 - g_{lum})}{(g_{lum} + 2) + 10^{A_f^R - A_f^L}(2 - g_{lum})} \tag{17}$$

And finally, by introducing the coefficient from equation (6):

$$g_{int} = 2\frac{(2 - g_{filter})(g_{lum} + 2) - (2 + g_{filter})(2 - g_{lum})}{(2 - g_{filter})(g_{lum} + 2) + (2 + g_{filter})(2 - g_{lum})} = 4\frac{g_{lum} - g_{filter}}{4 - g_{lum} g_{filter}} \tag{18}$$

The formula above connects the dissymmetry factors measured for the stacked sample, which exhibits only the filter effect, the thin film of AZO doped with PNCs, and the dissymmetry expected for the doped sample without the reabsorption effect. The only assumption made is that the absorption of excitation light is significantly higher than the absorption of emitted light.

It is worth noting that if g_{lum} and g_{filter} are small, their product can be neglected in the denominator, resulting in a simple additive relationship between the reabsorption and combined other three mechanisms contribution:

$$g_{lum} = g_{filter} + g_{int} \tag{19}$$

Supplementary Note 8.

To gain further insight into how the measured CPL varies with film thickness, we performed thickness-dependent CPL measurements.

Samples were fabricated with spacers of varying thickness: no spacer (≈4.5 μm, thinner than originally), silica spheres (≈8 μm, the original study), and aluminum foil (≈11 μm, thicker than originally). Thickness was confirmed by profilometry after removing the top glass slide, as the film tends to preferentially adhere to one part of the slide when opening the samples (see Figure S39). We prepared blue-NPL and red-NC based films of these thicknesses, which are expected to be the most and least sensitive to the varying filter effect, respectively. In all cases, we used the

NC doping level that gave the highest g_{lum} for the thickness used in the original study. For each sample, a series of well-separated points was measured (Figures S40 and S41).

Summarizing the results:

(i) AZO doped with blue-NPLs – g_{lum} values increased sevenfold as sample thickness increased from ≈4.5 μm to ≈11 μm (Table S1), consistent with the growing filtering effect;
(ii) AZO doped with red-NCs – g_{lum} values were statistically invariant for all sample thicknesses; namely, the g_{lum} values difference between the thinnest and thickest sample was smaller than the standard deviation of the value for the thinnest sample; this is consistent with the dominance of the intrinsic effects of CPL (Table S1).

Table S1. CPL dissymmetry factors measured for blue-NPLs and red-NCs samples with varying thicknesses.

Thickness [μm]	blue-NPL, g_{lum} at 460 nm	red-NCs, g_{lum} at 620 nm
4.5	0.013 ± 0.005	0.17 ± 0.07
8	0.047 ± 0.018	0.2 ± 0.06
11	0.094 ± 0.042	0.23 ± 0.04

An additional observation is that the intensity of emission decreases rapidly for thicker films, due to the longer optical path that the emitted light must travel through the AZO matrix. We also detect an apparent redshift of the emission band, which may be attributed to the AZO absorption band being stronger on the shorter-wavelength side of the emission.

Overall, the sample thickness dependence of CPL varies across different spectral ranges. These results demonstrate that for blue-emitting films, CPL is strongly thickness-dependent, in line with optical filtering contributions. However, for red-emitting films, CPL is thickness-independent, consistent with an intrinsic near-field origin of CPL.

Supplementary Note 9.

To determine the morphology and composition-related impact on CPL properties, we analyze the g_{int} values in detail.

First, we compare blue-NPLs and green-NCs, specifically focusing on platelet vs. cube morphology, both composed of $CsPbBr_3$. Electron microscopy revealed that nanocubes tend to form helical arrangements, while nanoplatelets are more likely to assemble into stacks consisting of 4 to 5 NCs. Thus, we could expect variation in the near-field interactions (g_{int}). Despite these differences, we observe that in both cases, g_{int} spectra are similar in both shape and magnitude, with maximum values around $g_{int} \approx 0.08$. This suggests that the particle shape (and thus the type of assemblies formed) is not the sole factor determining dissymmetry.

Second, we can compare green-NCs and red-NCs, which have the same shape but are made of $CsPbBr_3$ vs. $CsPb(BrI)_3$, respectively. In the case of red-NCs, the g_{int} spectra exhibit values approximately three times higher, with maximum values ≈0.24. Since electron microscopy did not

reveal meaningful differences between assemblies of these systems, this could suggest that the chemical composition of the perovskite is a more significant determinant of the g_{int} values than the shape of the nanoparticles. From this comparison, we tentatively conclude that the presence of heavier halides amplifies the CPL. There are three mechanisms consistent with these observations:

1) The $CsPb(Br/I)_3$ nanocrystals exhibit longer emission wavelengths and thus lower transition energies compared to $CsPbBr_3$. For similar oscillator strengths, this results in larger transition dipole moments, which strengthen the near-field dipole-dipole coupling between nanocrystals in the helical assemblies. Since the rotatory strength scales with the square of the transition dipole moment, this enhancement can directly increase the g_{int} value.
2) The higher polarizability of I-rich perovskites near their band edge can amplify local chiral fields in the assemblies, further boosting the observed circular polarization. Additionally, we note that $CsPb(Br/I)_3$ nanocrystals tend to assemble with slightly higher structural order under our preparation conditions, which also contributes to stronger chiroptical effects.
3) Perovskites naturally show strong spin–orbit coupling, and this can become even more pronounced in iodide-rich compositions because iodine is a heavier atom than bromine. In a chiral environment, such as the internal void of AZO nanotubes, spin-orbit coupling may result in slight energy differences between right- and left-circularly polarized emissions. This effect, sometimes referred to as a Rashba-like effect, can shift the balance between the two helicities, thereby increasing the circular polarization. While there is still some scientific debate about the strength of this effect in bulk perovskites, we cannot exclude it in our system.

Taken together, these factors (weaker filtering and stronger intrinsic effects) provide a consistent explanation for the more than one order-of-magnitude enhancement in g_{lum} for the red-NCs composite structures compared to their green-NCs-doped counterparts.

.

Supplementary Note 10.

Comparison of the current work with recently reported methods for CPL induction in PNCs.

We distinguish two fundamentally different approaches to imparting chirality to PNCs. The most widely studied methods include grafting chiral ligands onto the surface of achiral perovskite nanocrystals, incorporating chiral organic cations into the internal structure of perovskites, and synthesizing chiral-shaped nanocrystals. Although these approaches have advanced, further optimization of structure and composition is required, as the dissymmetry values achieved in this way are typically below 0.1.

In all of the above approaches, the light emitted from PNCs is intrinsically circularly polarized, as it originates from nanocrystals with a chiral internal structure or from nanocrystals placed in a chiral environment composed of other nanocrystals and/or a chiral matrix.

The second, alternative method for obtaining CPL-active materials with dissymmetry factors an order of magnitude higher involves embedding achiral PNCs into a matrix that selectively transmits light of only one handedness of circular polarization. In this approach, the light directly emitted from the nanocrystals is not circularly polarized. Instead, it becomes circularly polarized as it travels through the matrix, which acts as a selective filter. This method typically employs a

cholesteric liquid-crystalline matrix, which can exhibit such strong selective filtering that only one circular polarization is transmitted, leading to dissymmetry factor values approaching 2.

In this work, we present a material that exhibits contributions from both of the aforementioned mechanisms. The intrinsic dissymmetry factor, resulting from the chiral assembly of PNCs and/or interactions with the organic matrix, reaches 0.2. This is one of the highest values reported for this class of CPL mechanisms (Table S2, Figure 8). Additionally, we observe a filtering effect analogous to the cholesteric-based approach, with a maximum dissymmetry factor reaching 0.1, but with the opposite sign to the intrinsic contribution. This approach enables the tuning of the CPL properties of the film by selecting different types of PNCs. By varying the PNC type, we obtain materials with emissions at different wavelengths, thereby achieving a CPL response that combines varying contributions from both selective filtering and intrinsic chiral emission from PNCs in chiral assemblies and environment.

Table S2. Comparison of recently reported approaches to CPL of PNCs. Note that the highest dissymmetry values are reported for approaches in which the emission is not intrinsically circularly polarized (marked with *) . Instead, PNCs emit achiral light, which becomes circularly polarized as it travels through a matrix exhibiting selective filtering.

Mechanism of CPL	Emission wavelength	Maximum dissymmetry factor	Reference
Chiral assembly of PNCs and selective filtering of AZO matrix contribution	400-670 nm	-0.1*, 0.26	this work
chiral ligands	500-550 nm	0.07	[52]
chiral assembly of PNCs	400-640 nm	0.001	[53]
chiral surrounding (micelles)	500-550 nm	0.002	[54]
selective filtering from cholesteric	400-750 nm	1.5*	[55]
chiral cation, chirality transfer to another perovskite crystal	500-550 nm	0.006	[56]
chiral cation	520-600 nm	0.02	[57]
asssembly of chiral helical polymer/perovskite hybrid nanofibers	480-520 nm	0.03	[58]
coupling between the emission frequency of the perovskite and the cavity resonance frequency of the cholesteric photonic crystal structure acting as chiral matrix	450-650 nm	0.34	[59]
Coupling between the emission frequency of the perovskite with chiral metasurface acting as chiral matrix	520-650 nm	0.3	[60]
selective filtering in cholesteric	450-700 nm	1.5*	[61]
chiral assembly of achiral PNCs	480-520 nm	0.006	[62]
selective filtering in cholesteric	420-650 nm	1.9*	[63]

Supplementary Figures

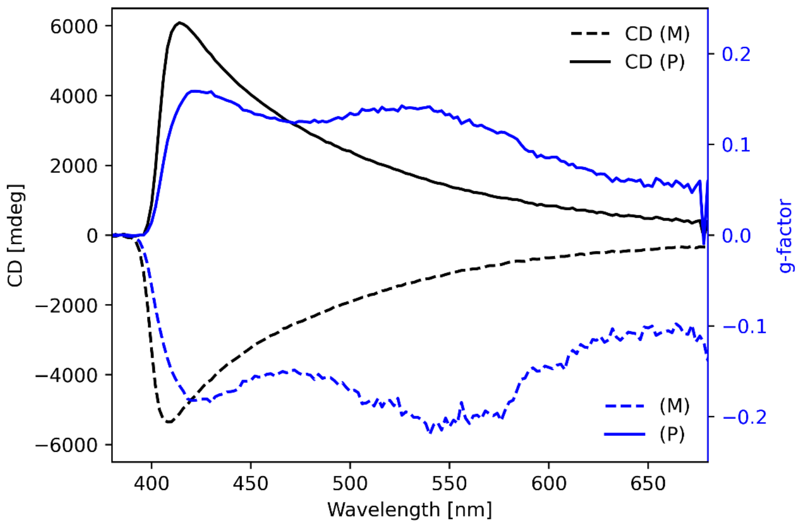

Figure S1. CD and g-factors of enantiomorphic homochiral domains (P and M). These data were obtained from Mueller matrix measurements of a ~10 μm-thick thin film of AZO nanotubes enclosed between two fused silica slides. The CD was calculated from the Mueller matrix using the differential matrix method [64].

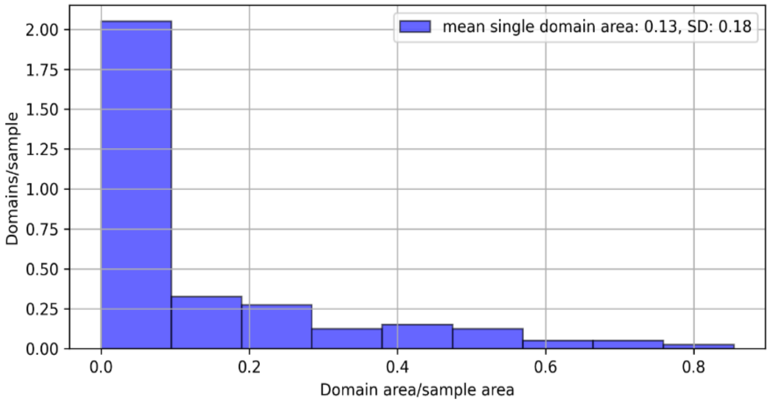

Figure S2. Size distribution of homochiral domains in AZO films cooled down at 2 °C/min rate expressed as the fraction of each domain's area relative to the total sample area (1.8 × 1.8 cm²). The histogram shows the number of domains per sample for each area fraction bin. The mean single-domain area is 0.13 ± 0.18 of the total sample area, corresponding to an absolute size of 0.42 ± 0.58 cm² (calculated as: 1.8 cm x 1.8 cm x (0.13 ± 0.18) = 0.42 ± 0.58 cm²).

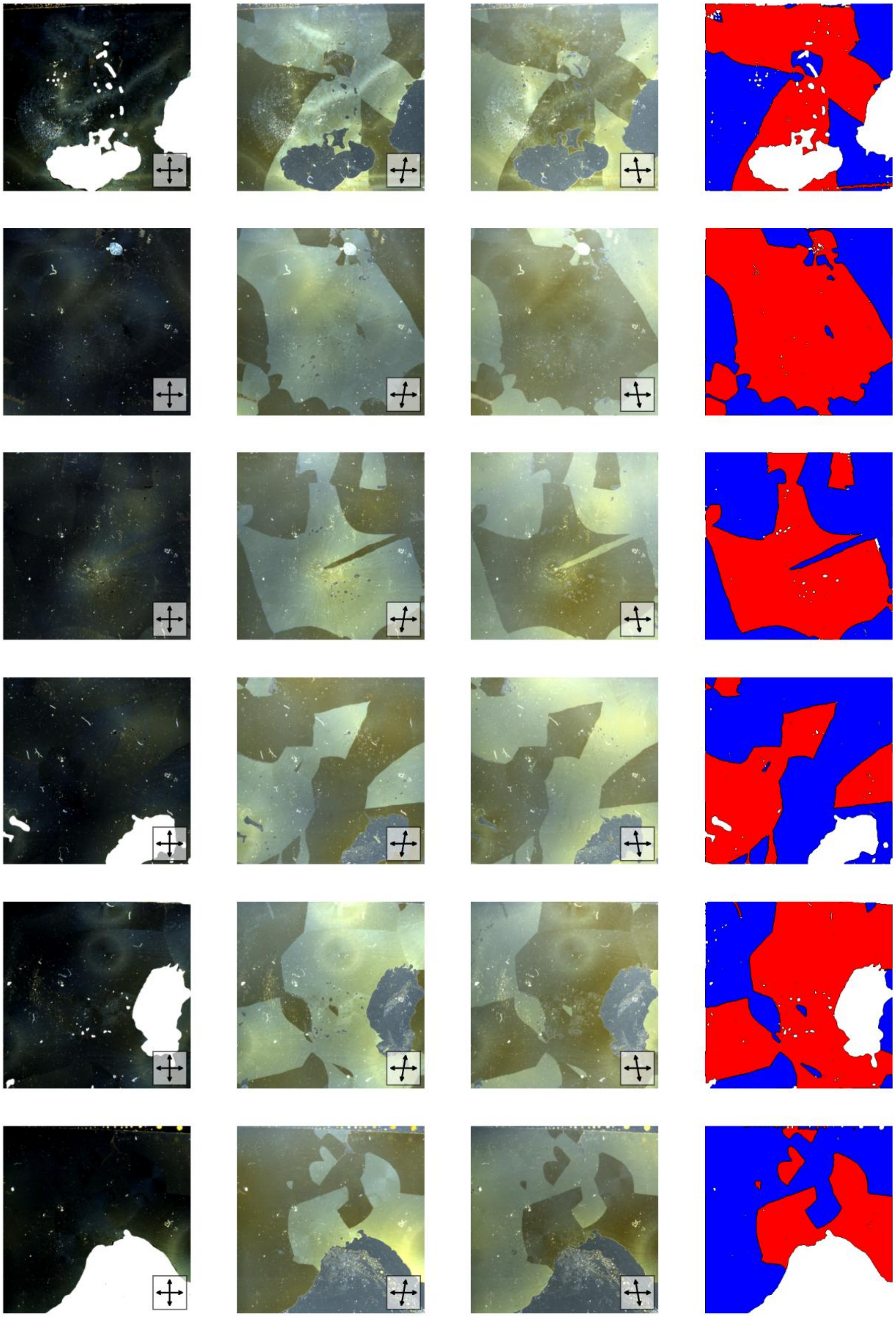

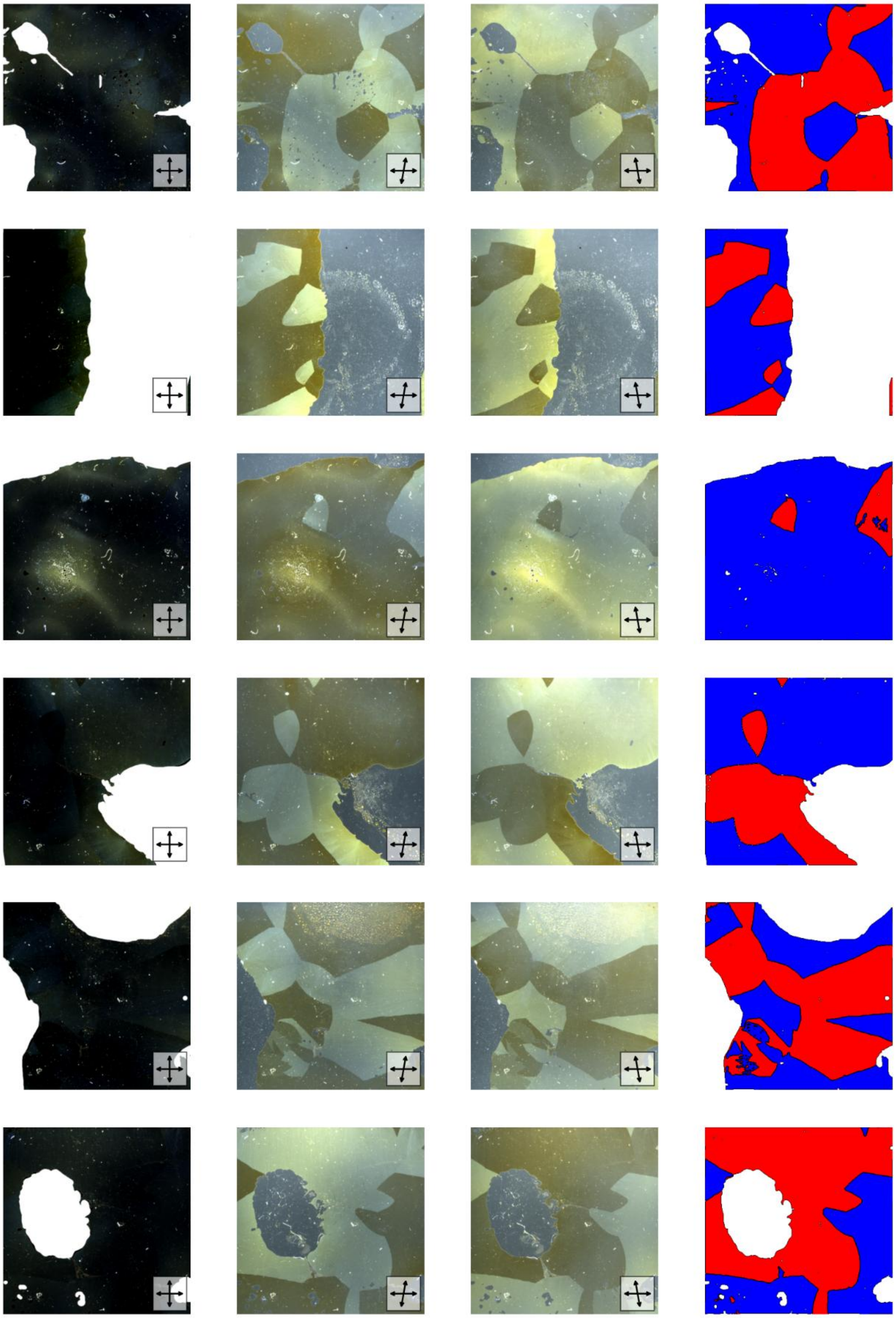

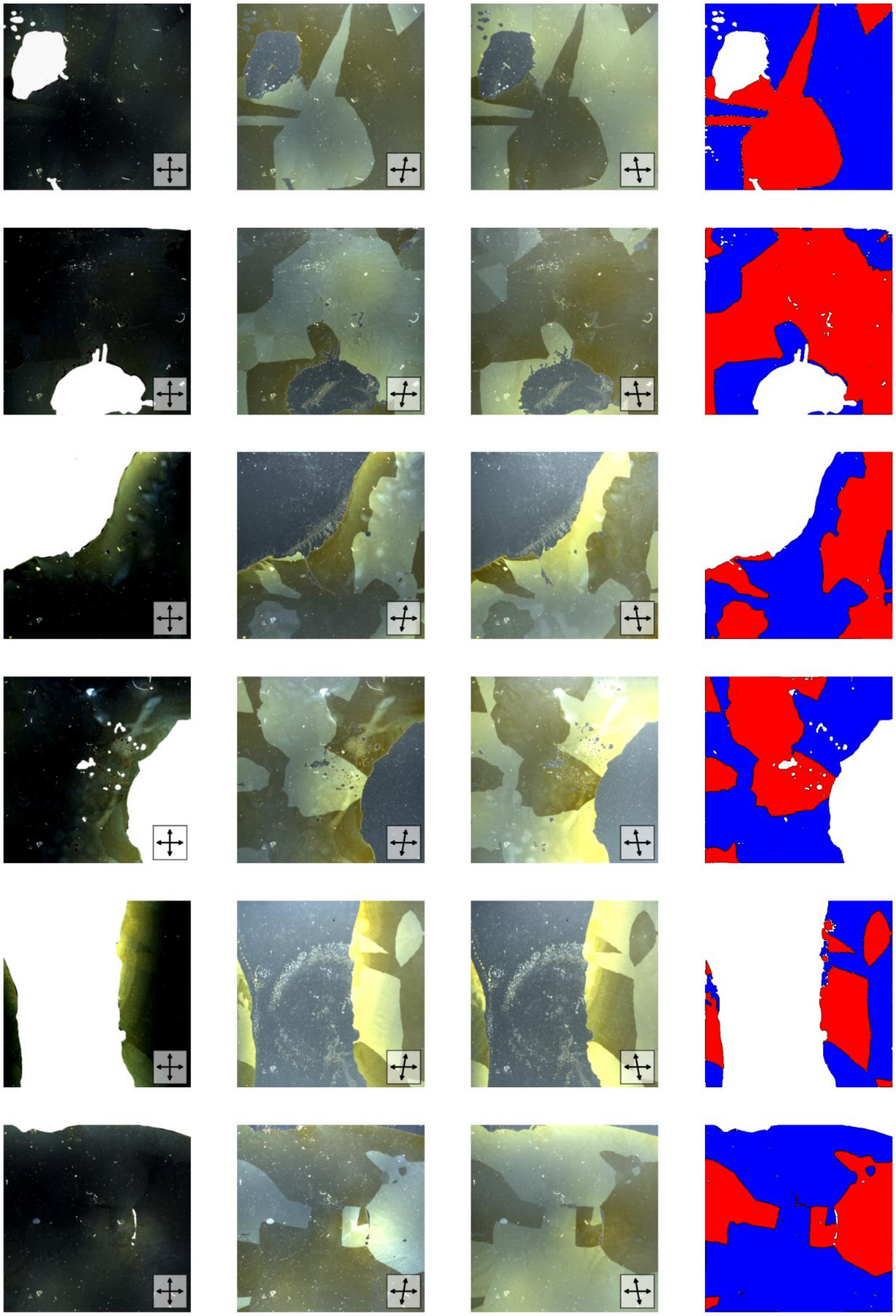

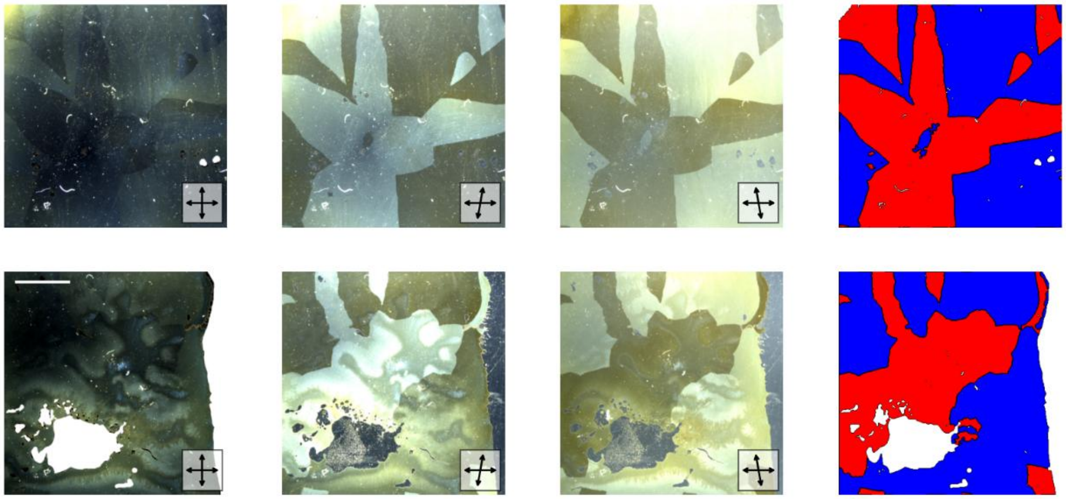

Figure S3. Polarized optical microscopy (POM) images of twenty AZO films, cooled at 2 °C/min rate. From left to right: (i) between crossed polarizers, (ii) with analyzer decrossed by +5°, (iii) with analyzer decrossed by -5°, and (iv) false-colored map identifying domains of opposite chirality (red and blue). These POM images enable the visualization and segmentation of homochiral domains based on their birefringence response under slight decrossing of the analyzer. Domain handedness identification was performed automatically as described in the Supplementary Note 1. The scale bar (placed in the image in the bottom row on the left) represents 5 mm.

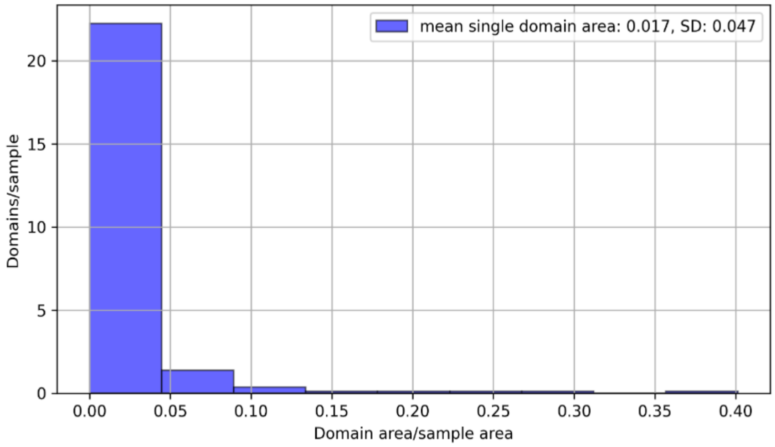

Figure S4. Size distribution of homochiral domains in AZO films cooled down at 10 °C/min rate expressed as the fraction of each domain's area relative to the total sample area (1.8 × 1.8 cm²). The histogram shows the number of domains per sample for each area fraction bin. The mean single-domain area is 0.017 ± 0.047 of the total sample area, corresponding to an absolute size of 0.055 ± 0.152 cm² (calculated as: 1.8 cm x 1.8 cm x (0.017 ± 0.047) = 0.055 ± 0.152 cm²).

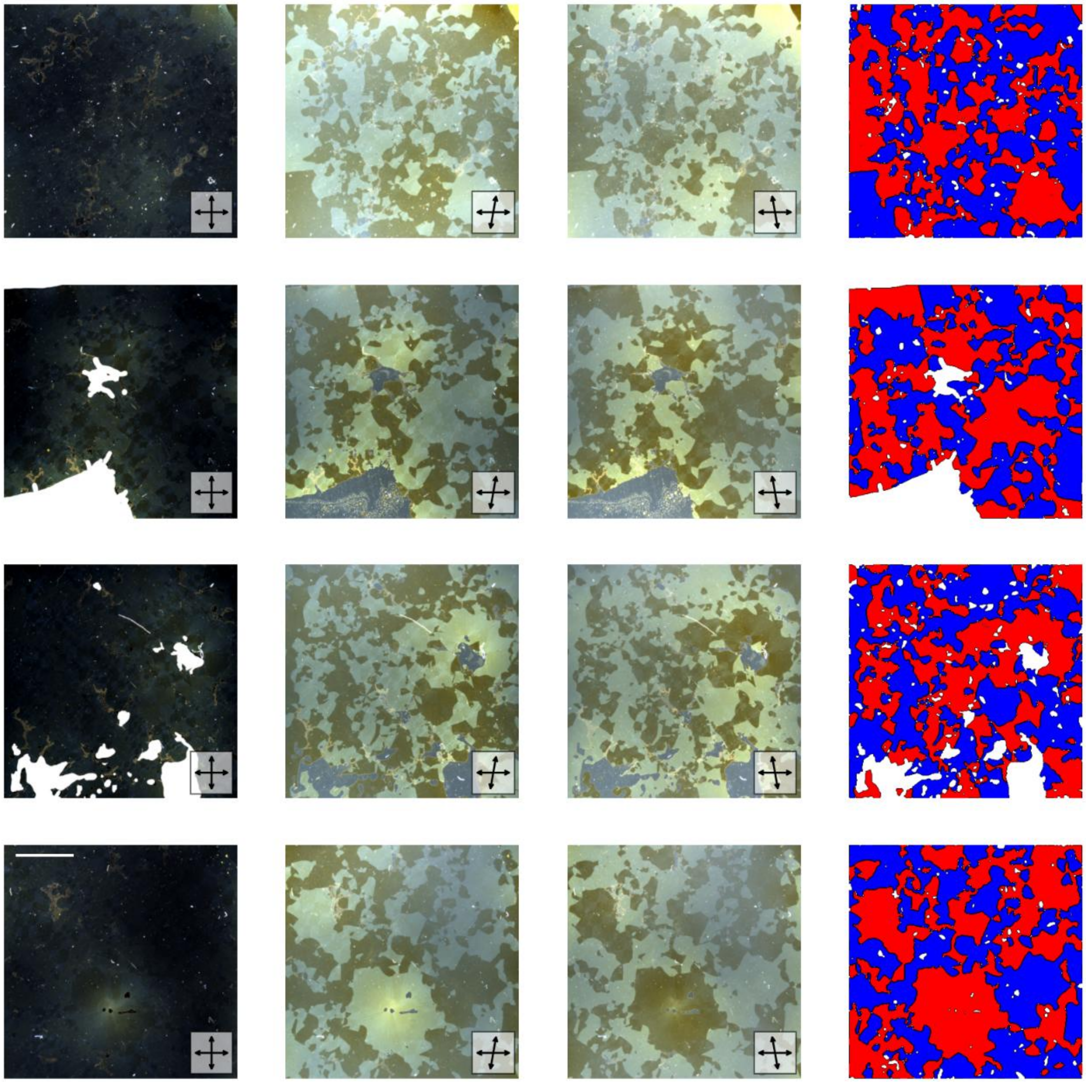

Figure S5. Polarized optical microscopy (POM) images of twenty AZO films, cooled at 10 °C/min rate. From left to right: (i) between crossed polarizers, (ii) with analyzer decrossed by +5°, (iii) with analyzer decrossed by -5°, and (iv) false-colored map identifying domains of opposite chirality (red and blue). These POM images enable the visualization and segmentation of homochiral domains based on their birefringence response under slight decrossing of the analyzer. Domain handedness identification was performed automatically as described in the Supplementary Note 1. The scale bar (placed in the image in the bottom row on the left) represents 5 mm.

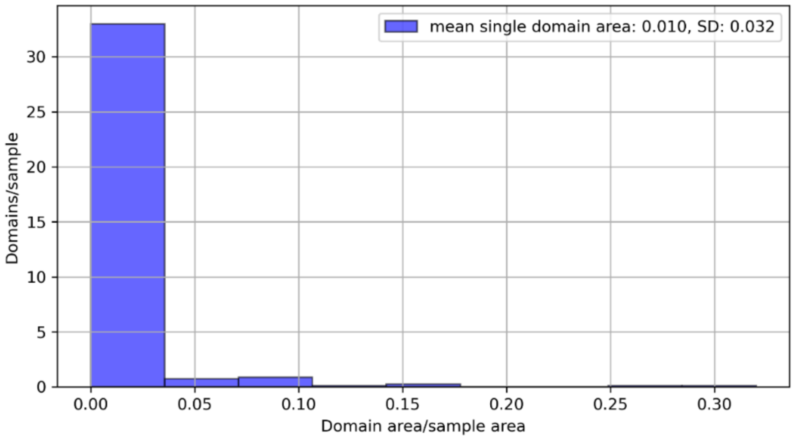

Figure S6. Size distribution of homochiral domains in AZO films cooled down at 10 °C/min rate, expressed as the fraction of each domain's area relative to the total sample area (1.8 × 1.8 cm²). The histogram shows the number of domains per sample for each area fraction bin. The mean single-domain area is 0.010 ± 0.032 of the total sample area, corresponding to an absolute size of 0.032 ± 0.104 cm² (calculated as: 1.8 cm x 1.8 cm x (0.010 ± 0.032) = 0.032 ± 0.104 cm²).

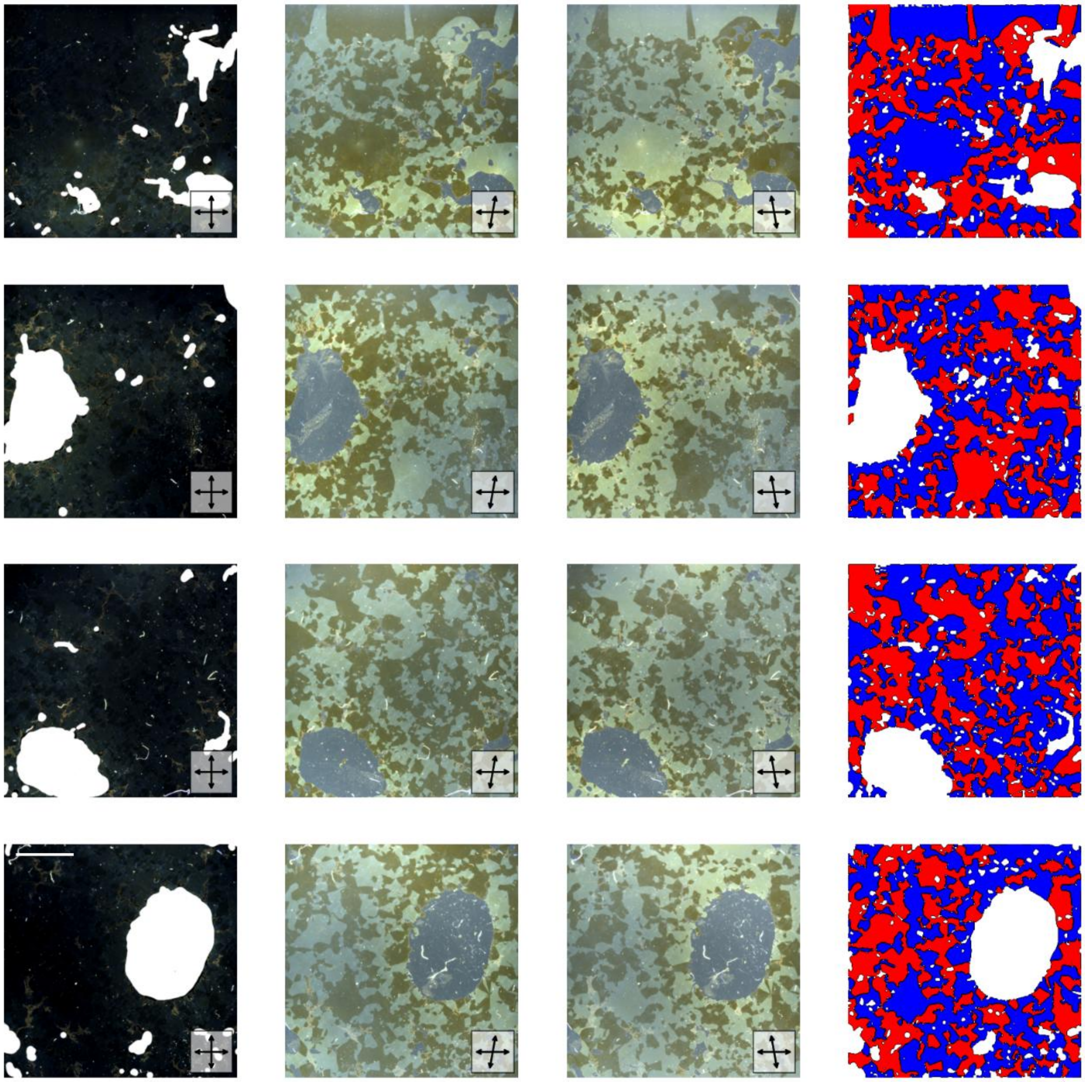

Figure S7. Polarized optical microscopy (POM) images of twenty AZO films, cooled at 20 °C/min rate. From left to right: (i) between crossed polarizers, (ii) with analyzer decrossed by +5°, (iii) with analyzer decrossed by -5 °, and (iv) false-colored map identifying domains of opposite chirality (red and blue). These POM images enable the visualization and segmentation of homochiral domains based on their birefringence response under slight decrossing of the analyzer. Domain handedness identification was performed automatically as described in the Supplementary Note 1. The scale bar (placed in the image in the bottom row on the left) represents 5 mm.

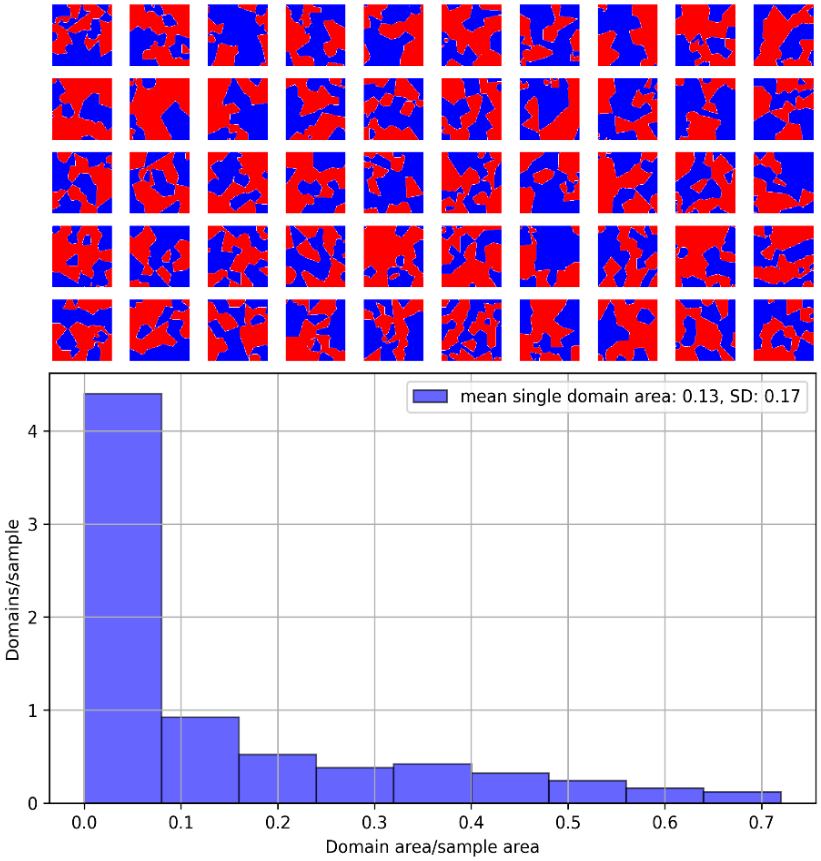

Figure S8. Simulation results for p/r = 100, yielding a domain size distribution with the same mean value as that observed experimentally for AZO films cooled at a rate of 2 °C/min.

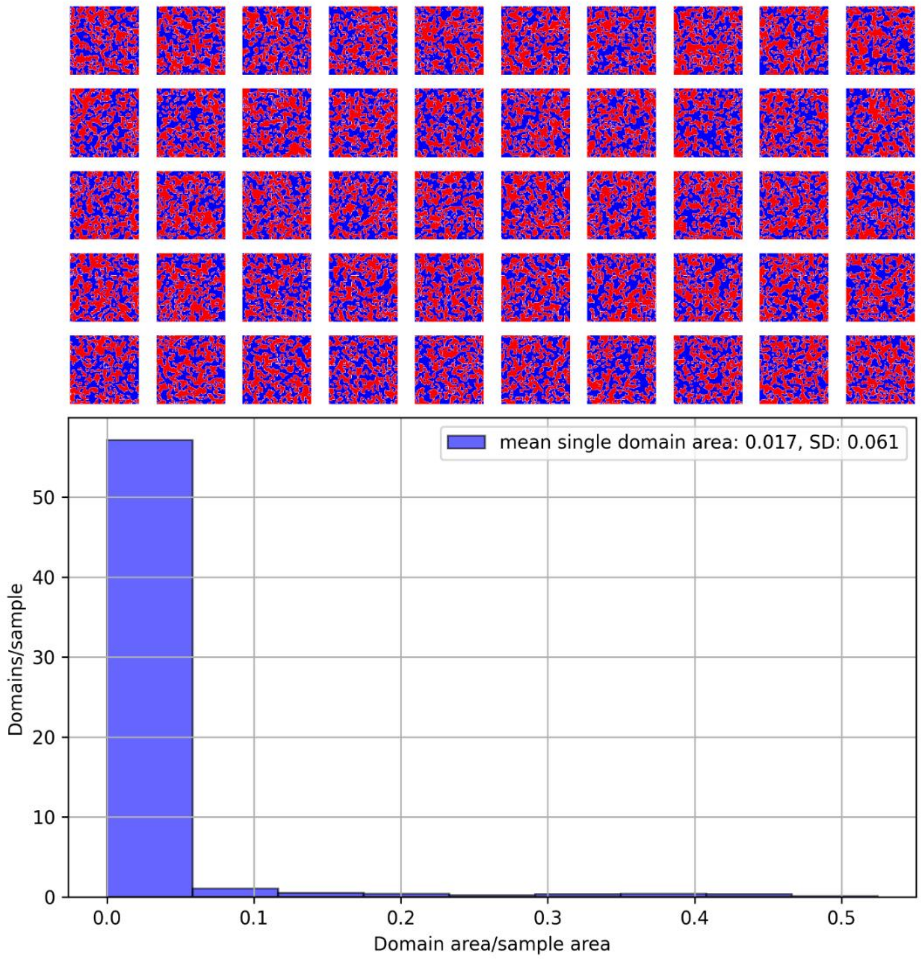

Figure S9. Simulation results for p/r = 100, yielding a domain size distribution with the same mean value as that observed experimentally for AZO films cooled at a rate of 10 °C/min.

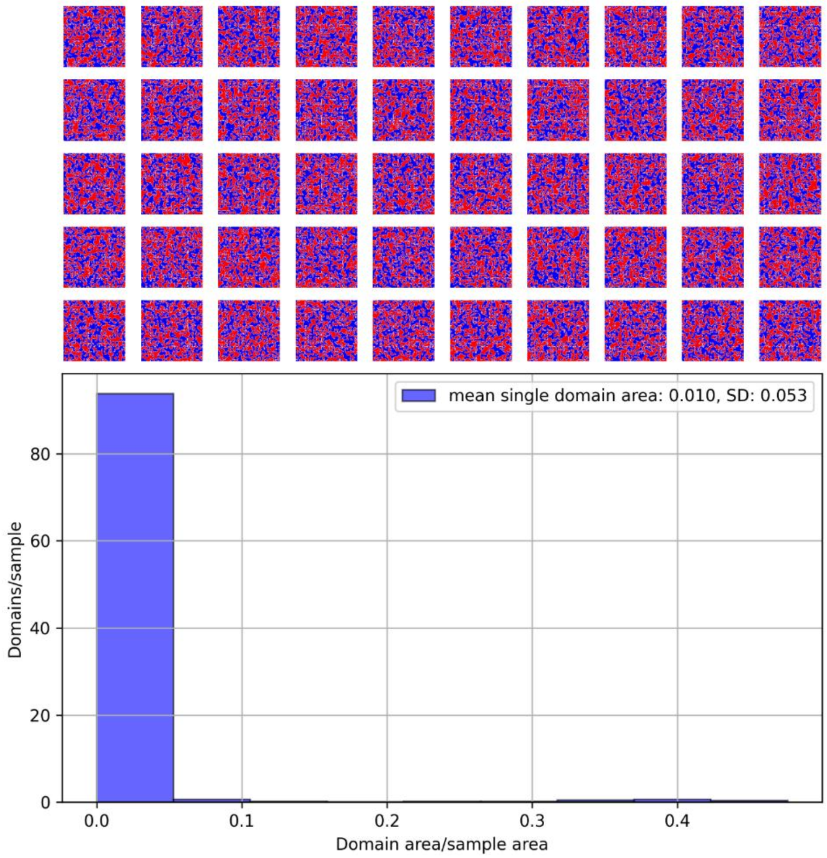

Figure S10. Simulation results for p/r = 100, yielding a domain size distribution with the same mean value as that observed experimentally for AZO films cooled at a rate of 20 °C/min.

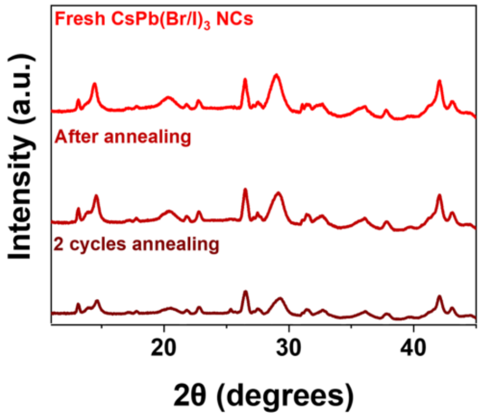

Figure S11. XRD patterns of $CsPb(Br/I)_3$ perovskite nanocrystals measured on: (a) a freshly prepared sample, (b) a sample after one heat-annealing step at 115 °C, (c) a sample after two heat-annealing cycles, and (d) simulated XRD data for comparison. Since the iodide ion (I^-) is larger than the bromide (Br^-), the reflections occur at slightly smaller 2θ than in $CsPbBr_3$.

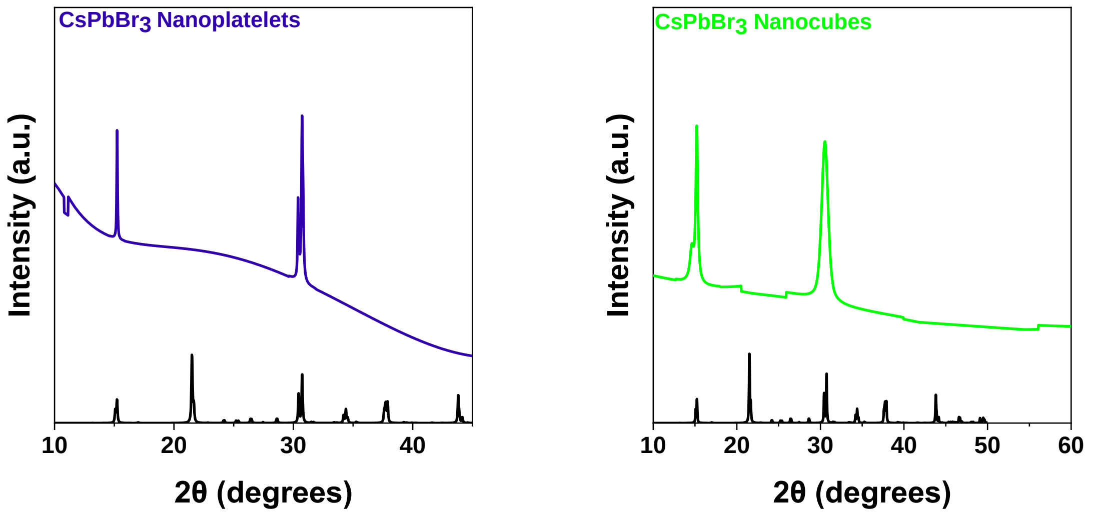

Figure S12. XRD diffraction patterns of blue-NPls and green-NCs used for the preparation of CPL-active films.

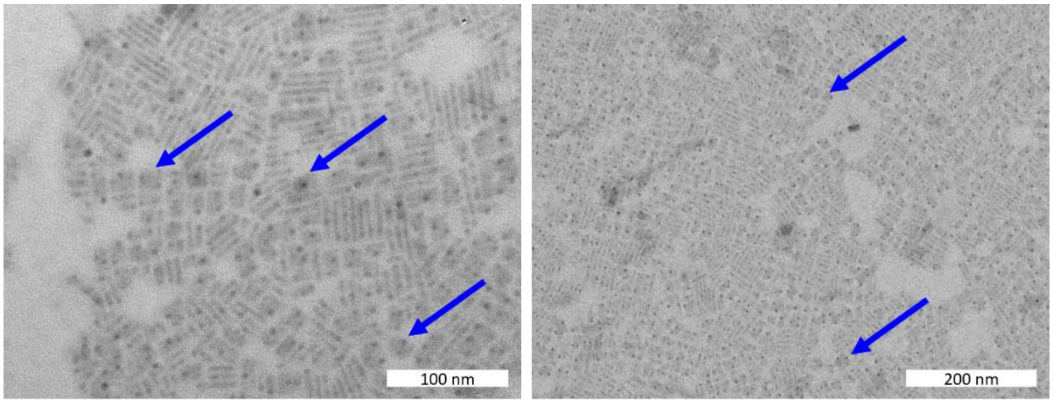

Figure S13. TEM images of NPLs drop-cast onto a TEM grid. The majority of the NPLs are oriented vertically. Arrows mark NPLs lying flat on the substrate.

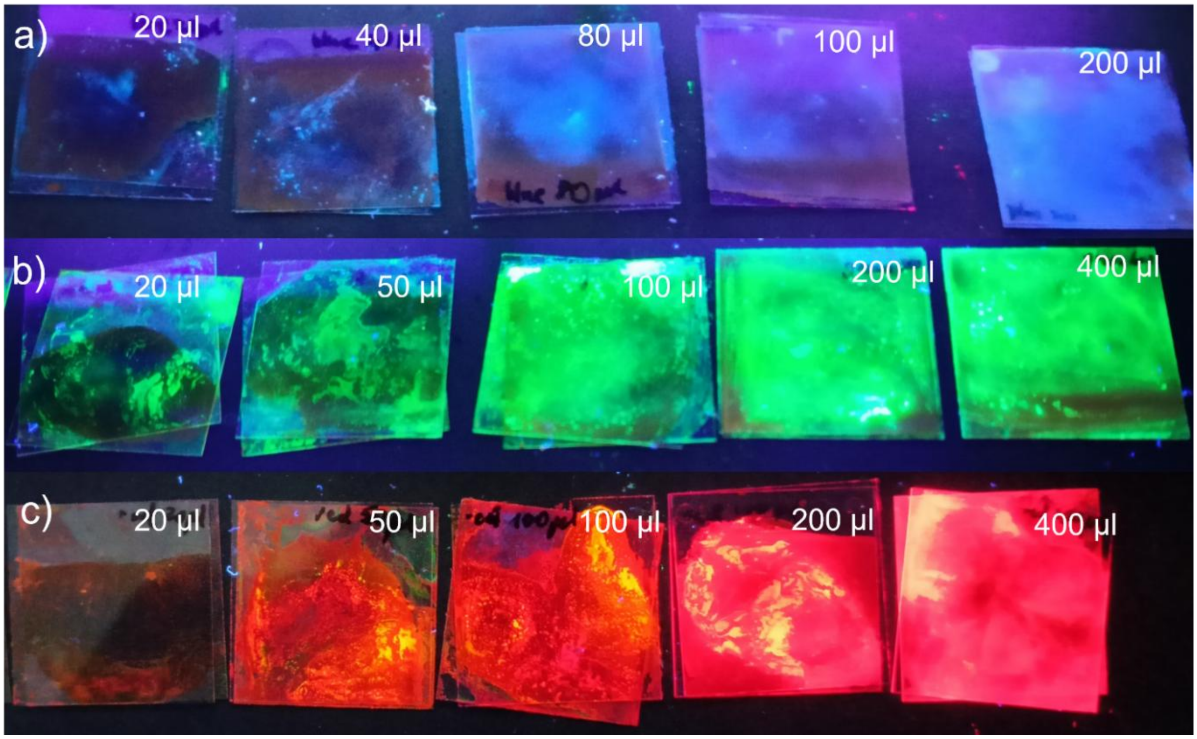

Figure S14. Optical photographs of AZO doped with a) blue-NPLs, b) green-CNs, and c) red-NCs, excited using a UV lamp with an intensity maximum at 365 nm. The length of the glass slide side is 18 mm.

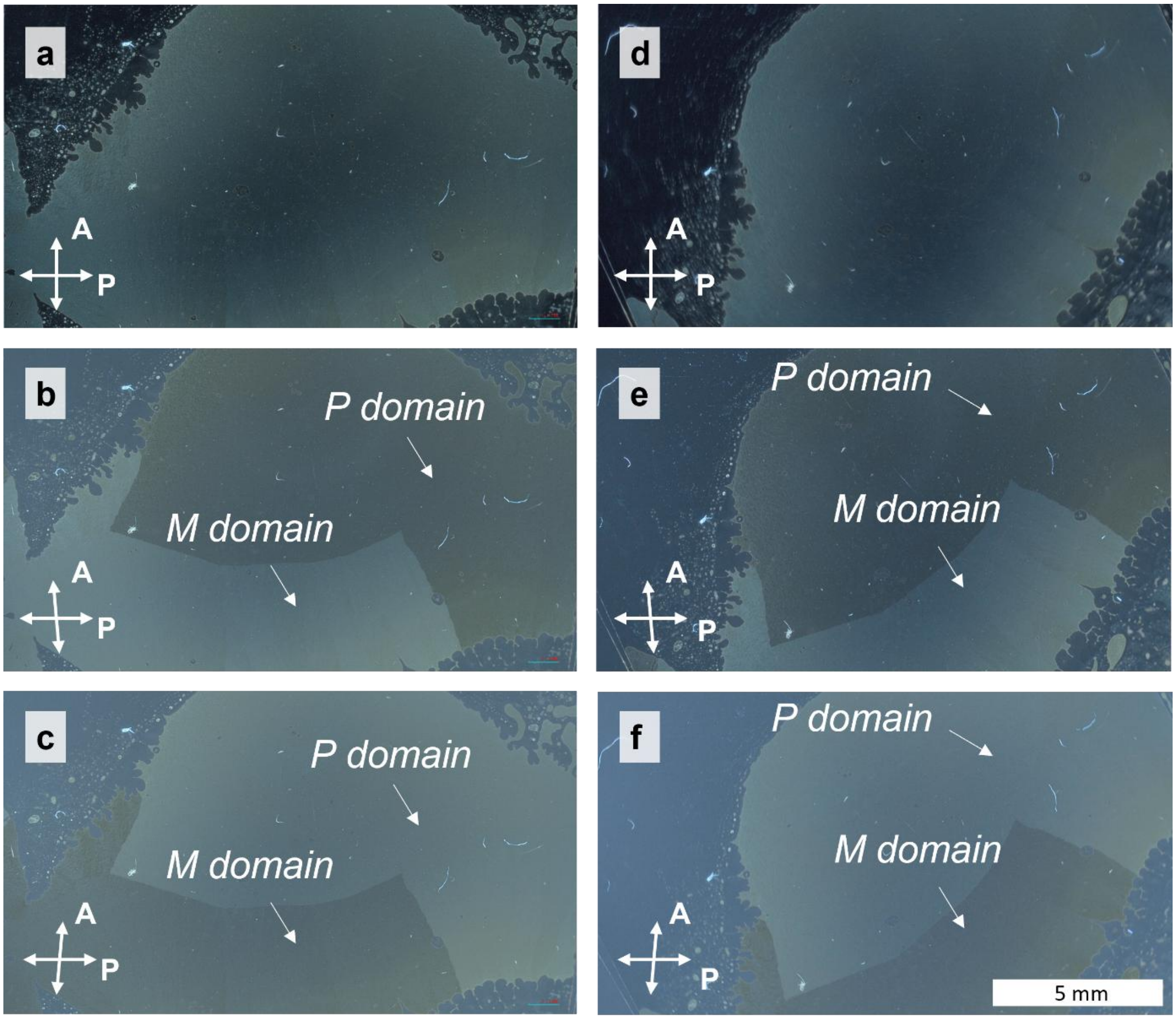

Figure S15. Visualization and discrimination of homochiral domains in AZO thin films doped with 100 µL of green-NCs, observed between two linear polarizers. The homochiral AZO domains exhibit optical activity, rotating linearly polarized light in opposite directions. This effect can be visualized by placing the sample between a polarizer (which creates linearly polarized light) and an analyzer, and then decrossing the analyzer (forming an angle of 85° or 95° instead of 90°). As a result, clockwise-rotating (P) domains appear brighter than counterclockwise-rotating (M) domains or vice versa, due to differential rotation of the polarization plane. This brightness contrast enables clear discrimination between P and M domains. (a, d) Images of the same sample rotated by 45°, when placed between crossed polarizers. (b, e) Images of the same sample, rotated by 45° when placed between decrossed polarizers, show no significant brightness changes, confirming the low optical anisotropy of the chiral domains; note that the arrows point to the same spot in both images. (c, f) POM images corresponding to those shown in panels b and e, but with polarizers decrossed in the opposite direction. Supplementary Movies S1–S3 present the full 360° rotation of the sample.

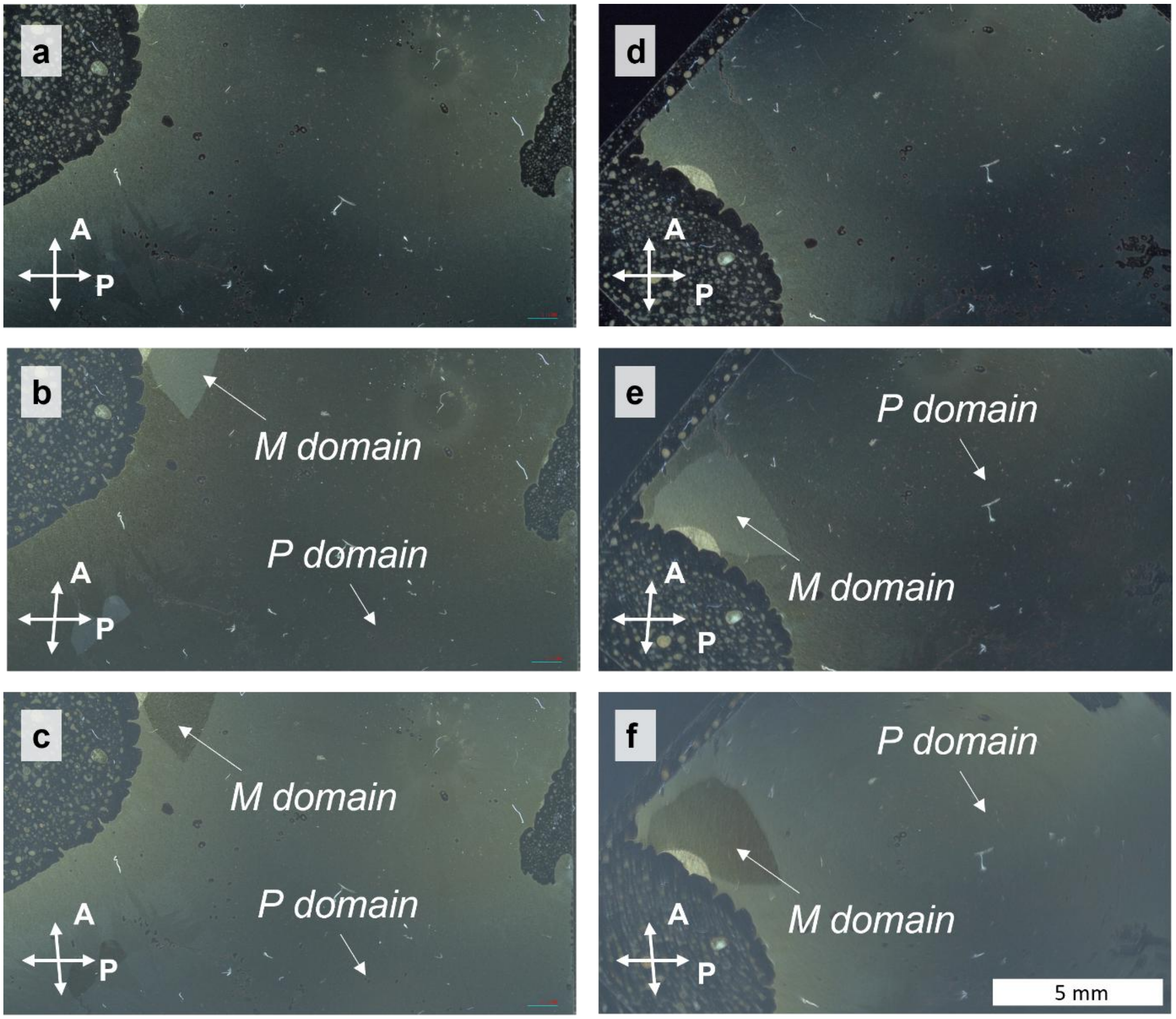

Figure S16. Visualization and discrimination homochiral domains in AZO thin films doped with 100 μL of red-NCs, observed between two linear polarizers. The homochiral AZO domains exhibit optical activity, rotating linearly polarized light in opposite directions. This effect can be visualized by placing the sample between a polarizer (which creates linearly polarized light) and an analyzer, and then decrossing the analyzer (forming an angle of 85° or 95° instead of 90°). As a result, clockwise-rotating (P) domains appear brighter than counterclockwise-rotating (M) domains or vice versa, due to differential rotation of the polarization plane. This brightness contrast enables clear discrimination between P and M domains. (a, d) Images of the same sample rotated by 45°, when placed between crossed polarizers. (b, e) Images of the same sample, rotated by 45° when placed between decrossed polarizers, show no significant brightness changes, confirming the low optical anisotropy of the chiral domains; note that the arrows point to the same spot in both images. (c, f) POM images corresponding to those shown in panels b and e, but with polarizers decrossed in the opposite direction. Supplementary Movies S4–S6 present the full 360° rotation of the sample.

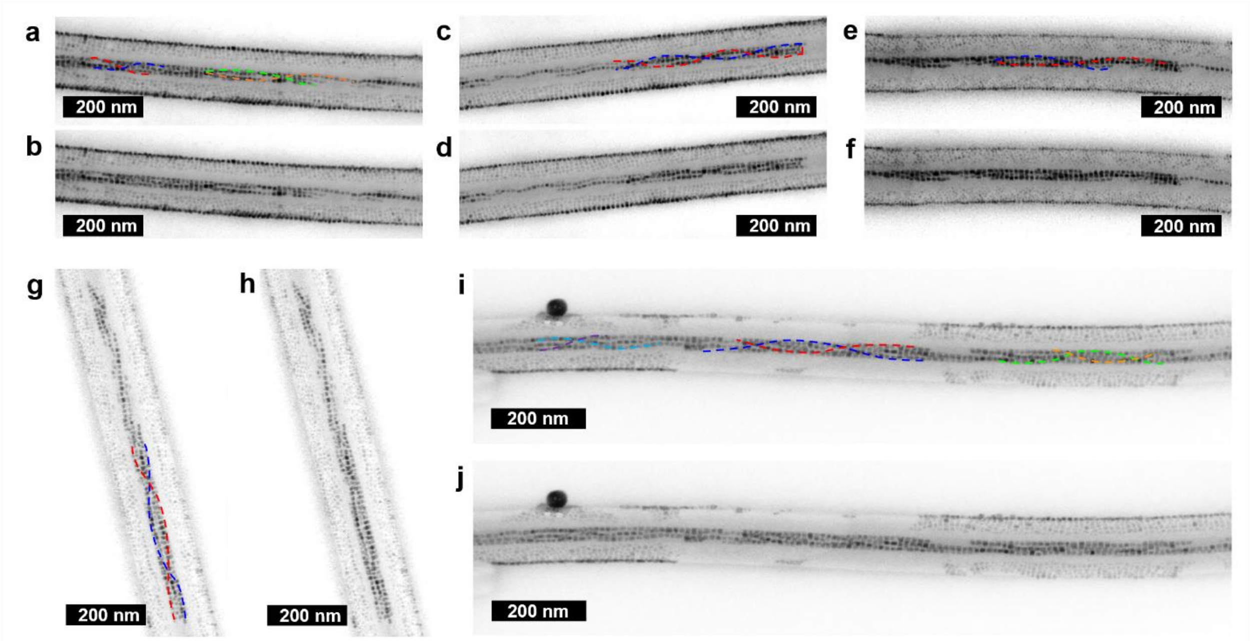

Figure S17. TEM images of helical assemblies of PNCs found in composites of green-NCs doped to AZO matrix, achieved using method **3**

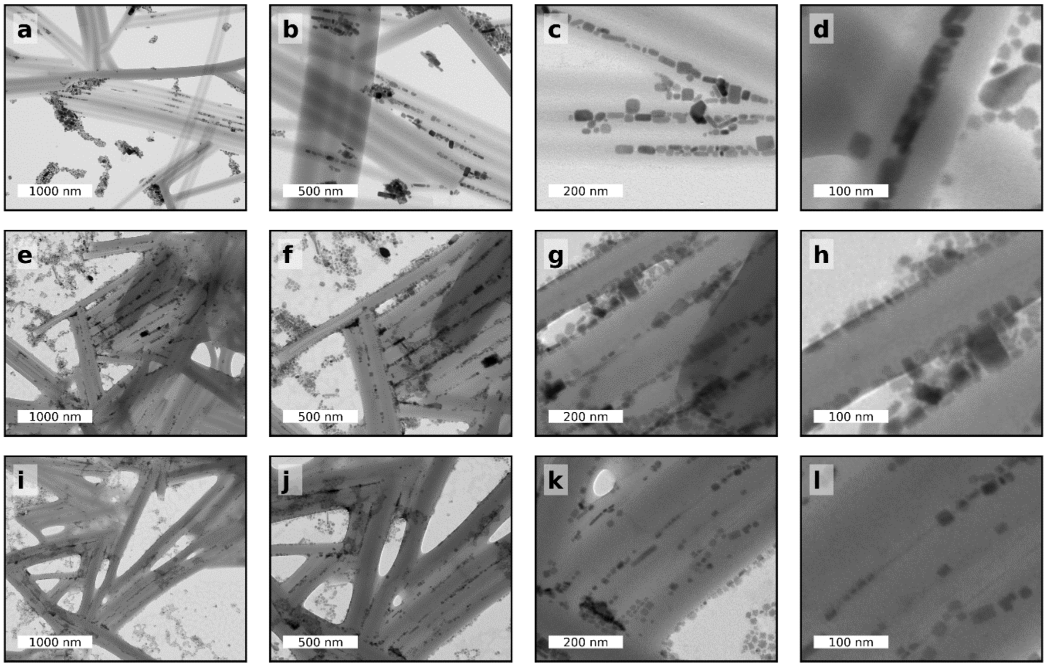

Figure S18. TEM images of composites: blue-NPLs doped to AZO matrix, achieved using method 5.

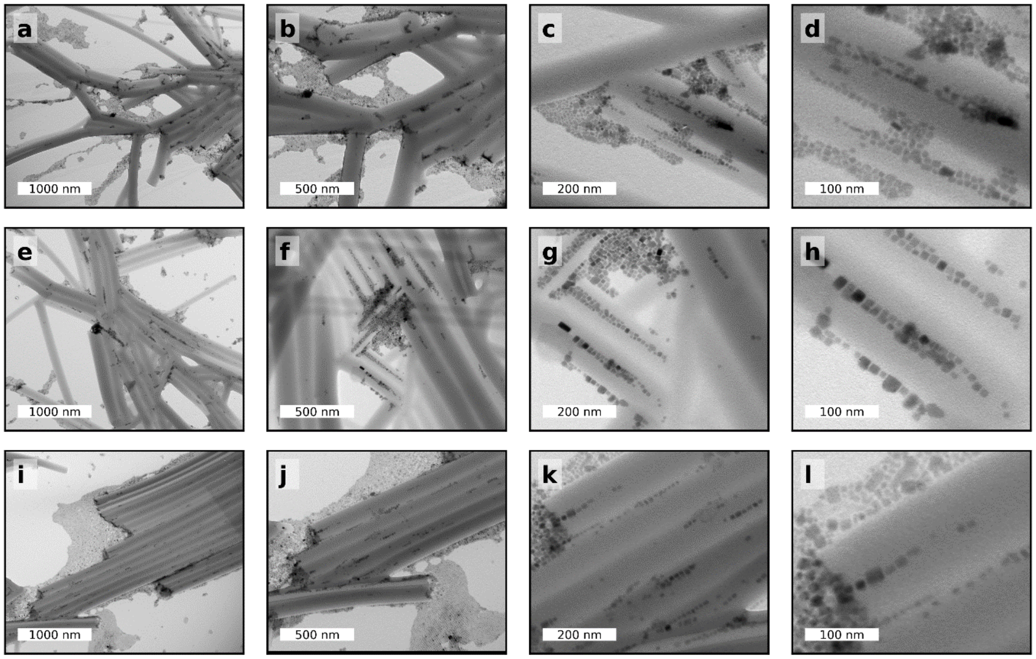

Figure S19. TEM images of various assemblies found on samples of green-NCs doped in AZO matrix, achieved using method 5.

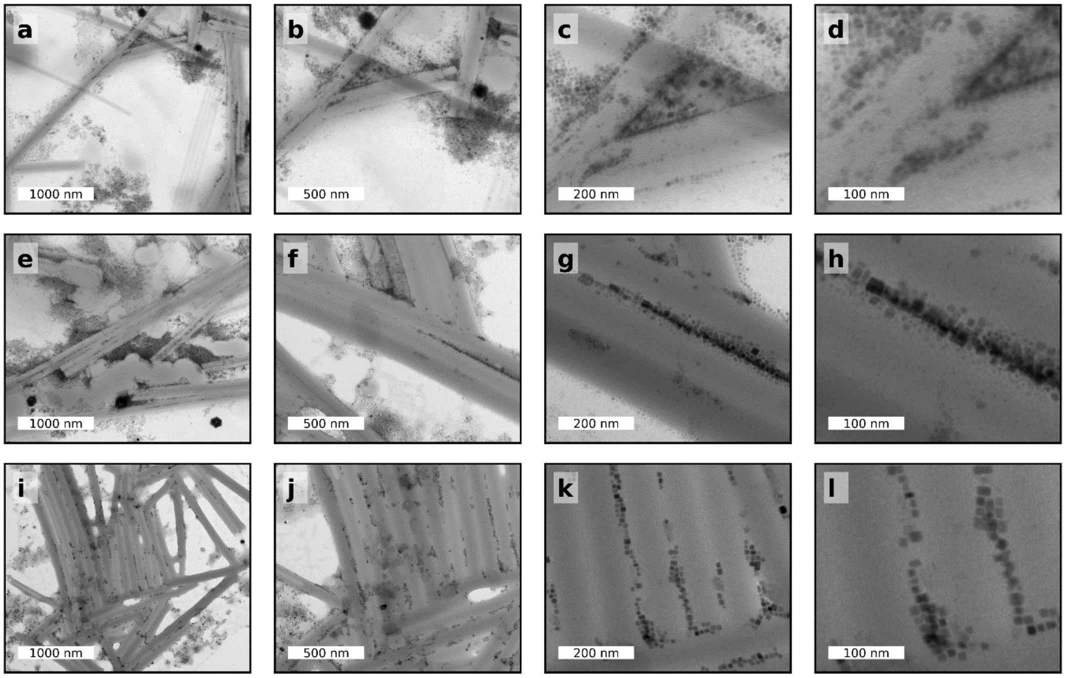

Figure S20. TEM images of various assemblies found on samples of green-NCs doped in AZO matrix, achieved using method 5.

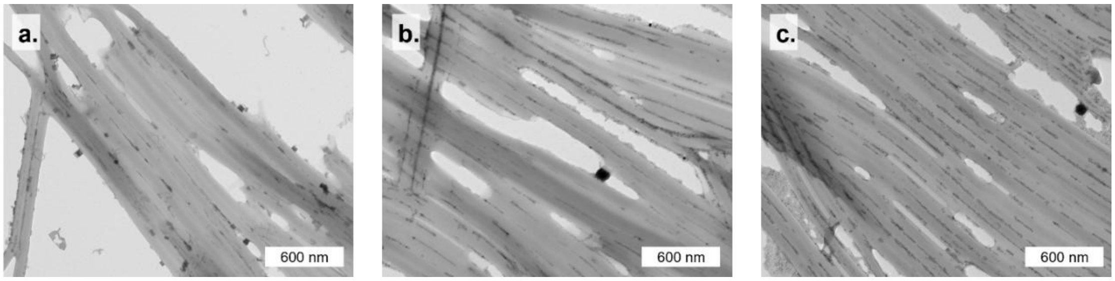

Figure S21. TEM images of assemblies of a) blue-NCs, b) green-NCs, c) red-NCs obtained from samples prepared by drop-casting a mixture of PNCs and AZO solutions, achieved using method 4.

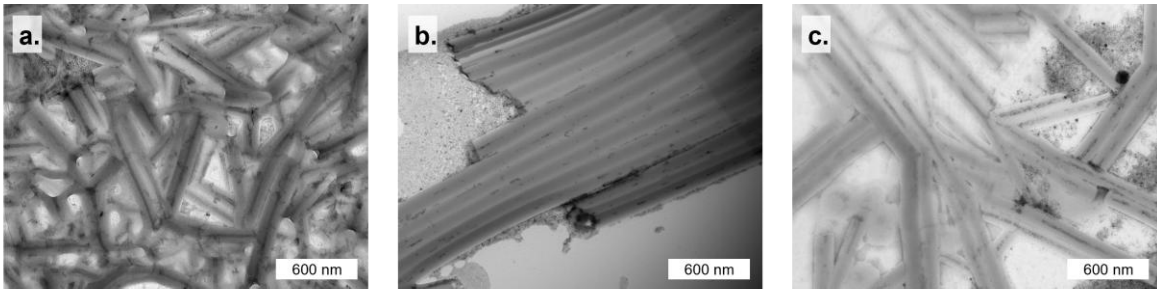

Figure S22. TEM images of assemblies of a) blue-NCs, b) green-NCs, c) red-NCs obtained from samples achieved using method 5.

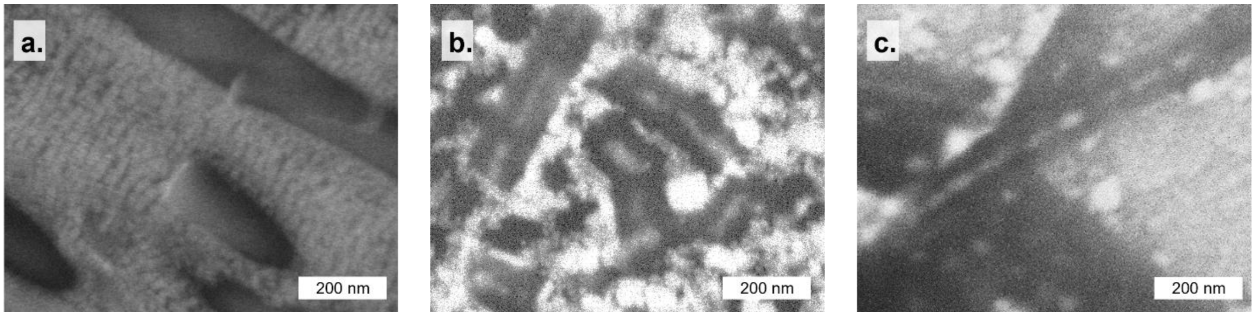

Figure S23. SEM images of assemblies of a) blue-NCs, b) green-NCs, c) red-NCs obtained from samples achieved using method 1.

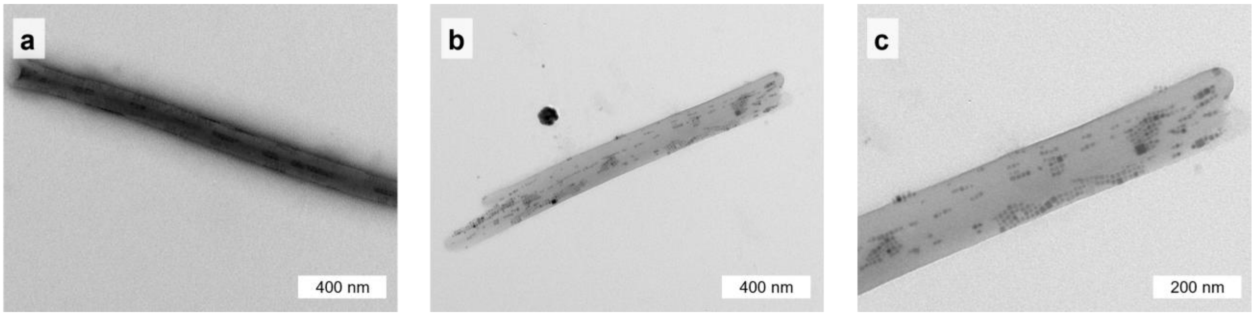

Figure S24. TEM images of assemblies of a) blue-NCs, b and c) green-NCs obtained from samples achieved using method 2**.**

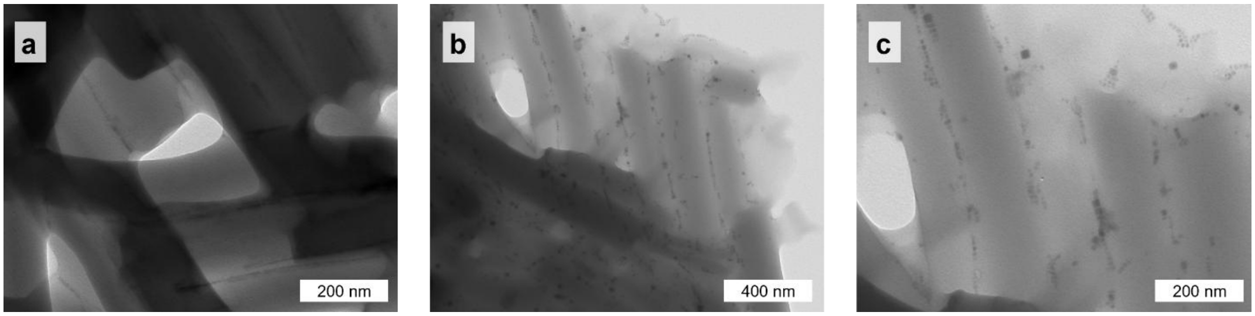

Figure S25. TEM images of assemblies of a) blue-NCs, b and c) green-NCs obtained from samples, achieved using method 3.

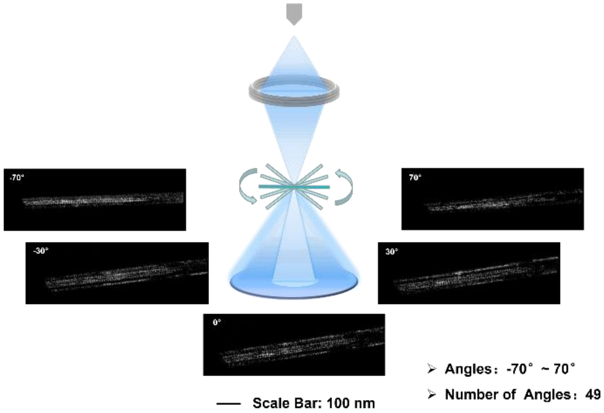

Figure S26. Schematic of the electron tomography experiment.

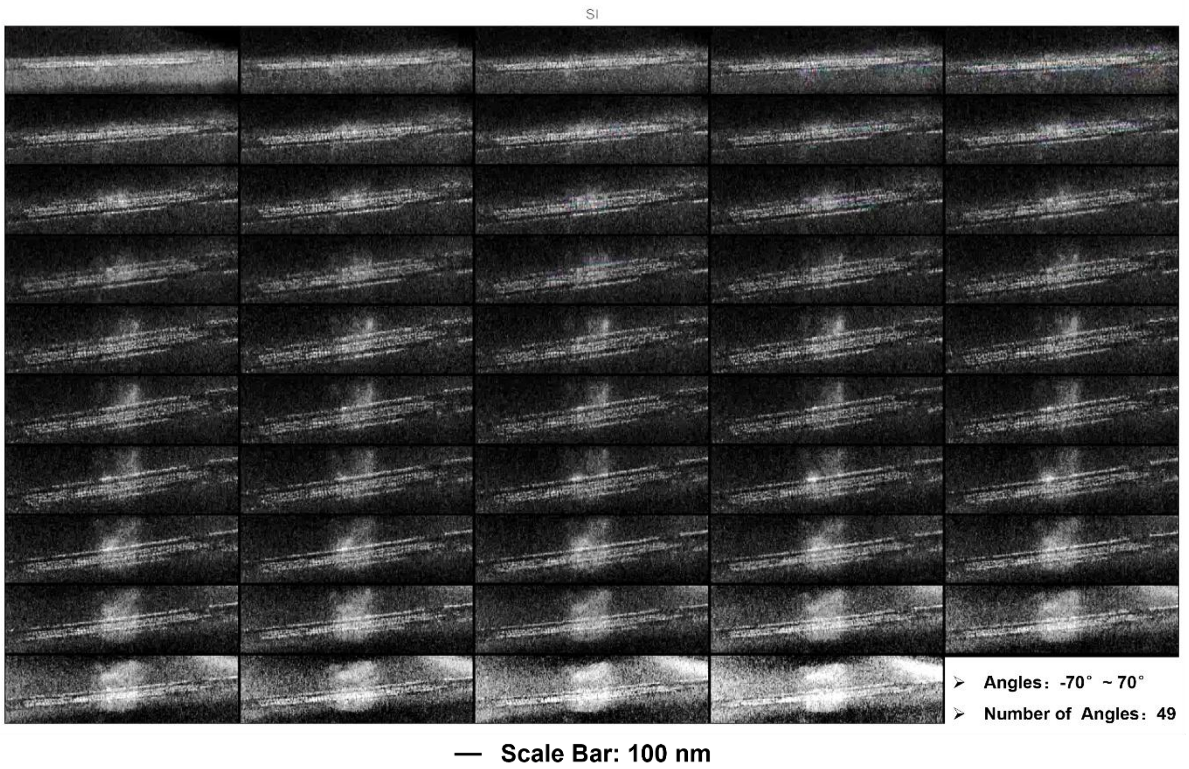

Figure S27. A representative tomographic tilt series after image processing. The 49 projection images, with a tilt range of –70° to 70°, were measured using ADF-STEM.

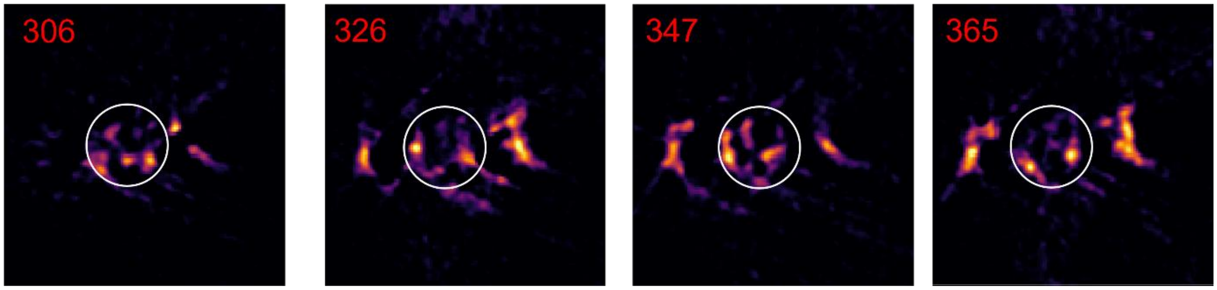

Figure S28. Tomographic slices taken along the axis of the helical structure of PNCs in a chiral nanotube. The white line circumference has a diameter of 38 nm. Two sets of nanocrystal assemblies are found: (i) ‘outer nanocrystals’ - deposited on the outer size of the nanotube - the diameter of a cylinder onto which the outer particles are deposited conforms to ≈76 nm, matching the dimensions of the external wall of the nanotube observed in the STEM-HAADF image and (ii) helically arranged ‘inner nanocrystals’ - deposited inside the nanotube - the diameter of the circumference onto which the inner nanoparticles are deposited is ≈36 nm. Notably, the tomographic reconstruction revealed a distinct helical character of these ‘inner’ nanocrystal assemblies (see Figure 5 in the main text). Particularly, slices through the tomography reconstruction taken along the y direction show the rotation of paired particles from slice to slice, indicating that the particles inside the cylinder wind around the axis and advance helically. Tomography slices, as well as tomograms viewed along the x-direction, directly visualize the helical path inside the nanotube, confirming the helical arrangement of perovskite nanocrystals in this confined geometry.

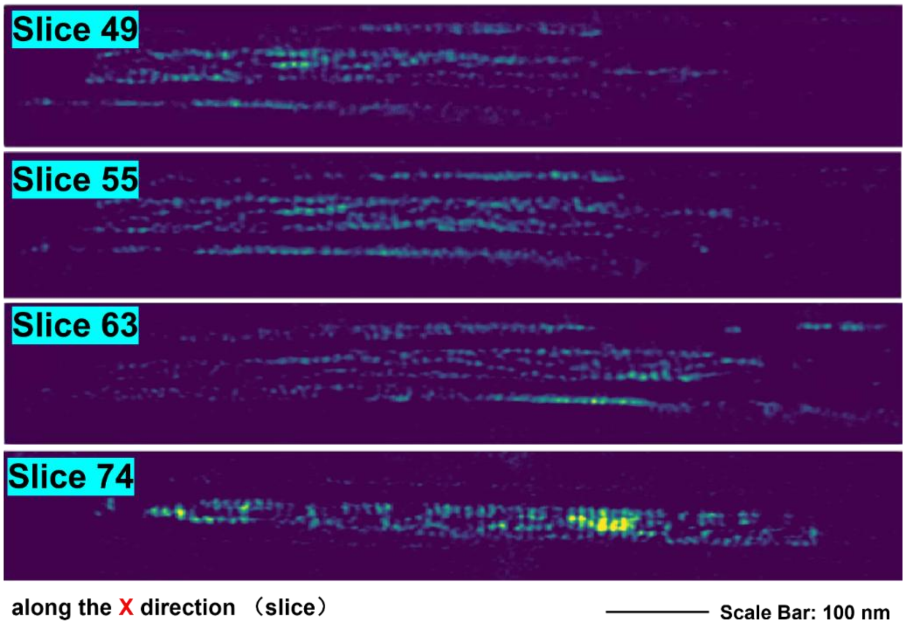

Figure S29. Tomographic slices taken perpendicular to the axis of the helical structure of PNCs in AZO nanotube.

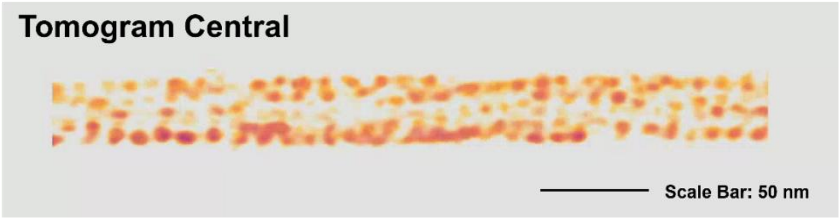

Figure S30. Tomogram of helical PNCs assembly inside AZO nanotube (inner nanocrystals, see caption of Figure S28 for details).

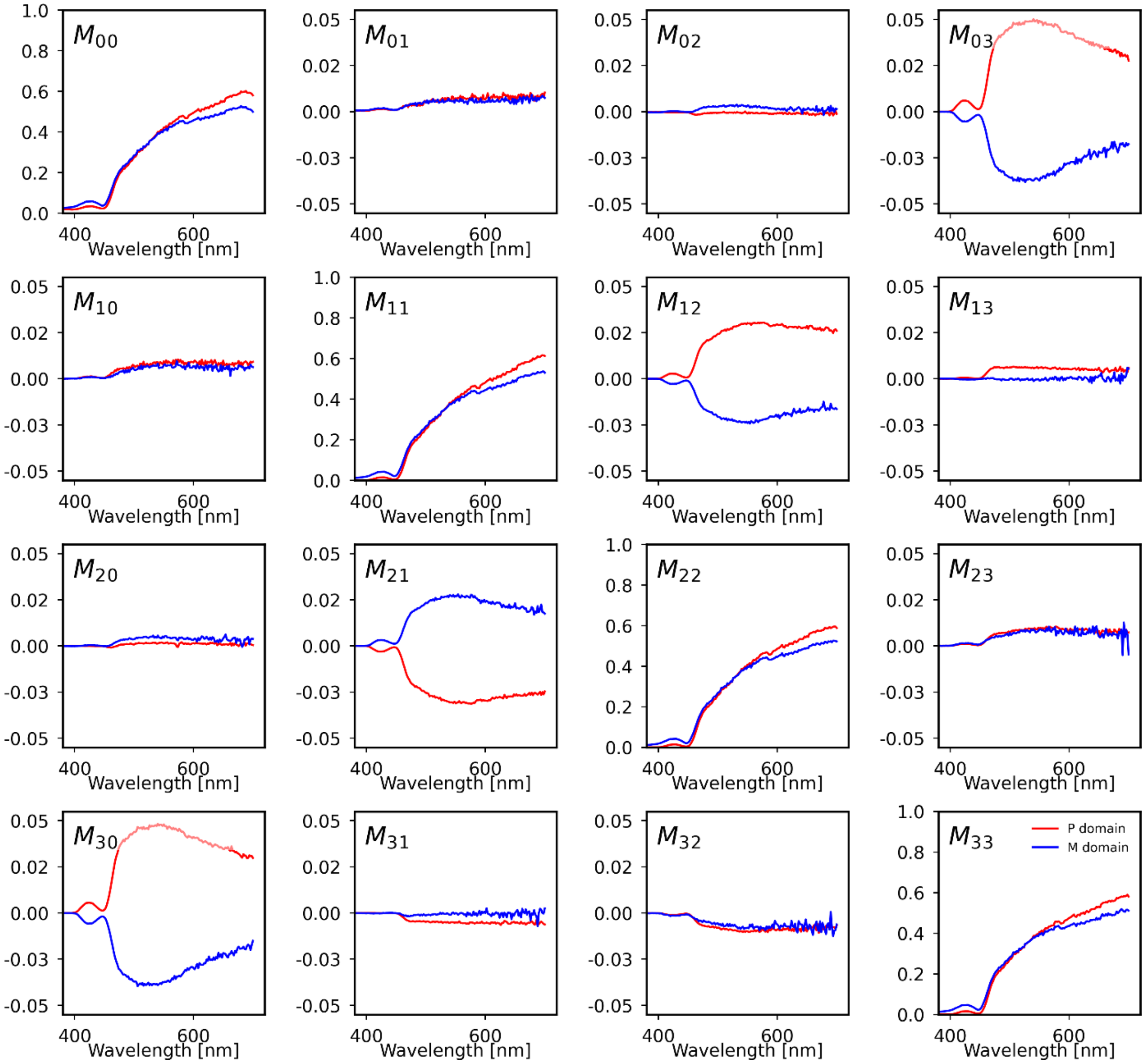

Figure S31. Raw Mueller Matrices measured for a thin film of AZO doped with 80 µl of blue-NCs.

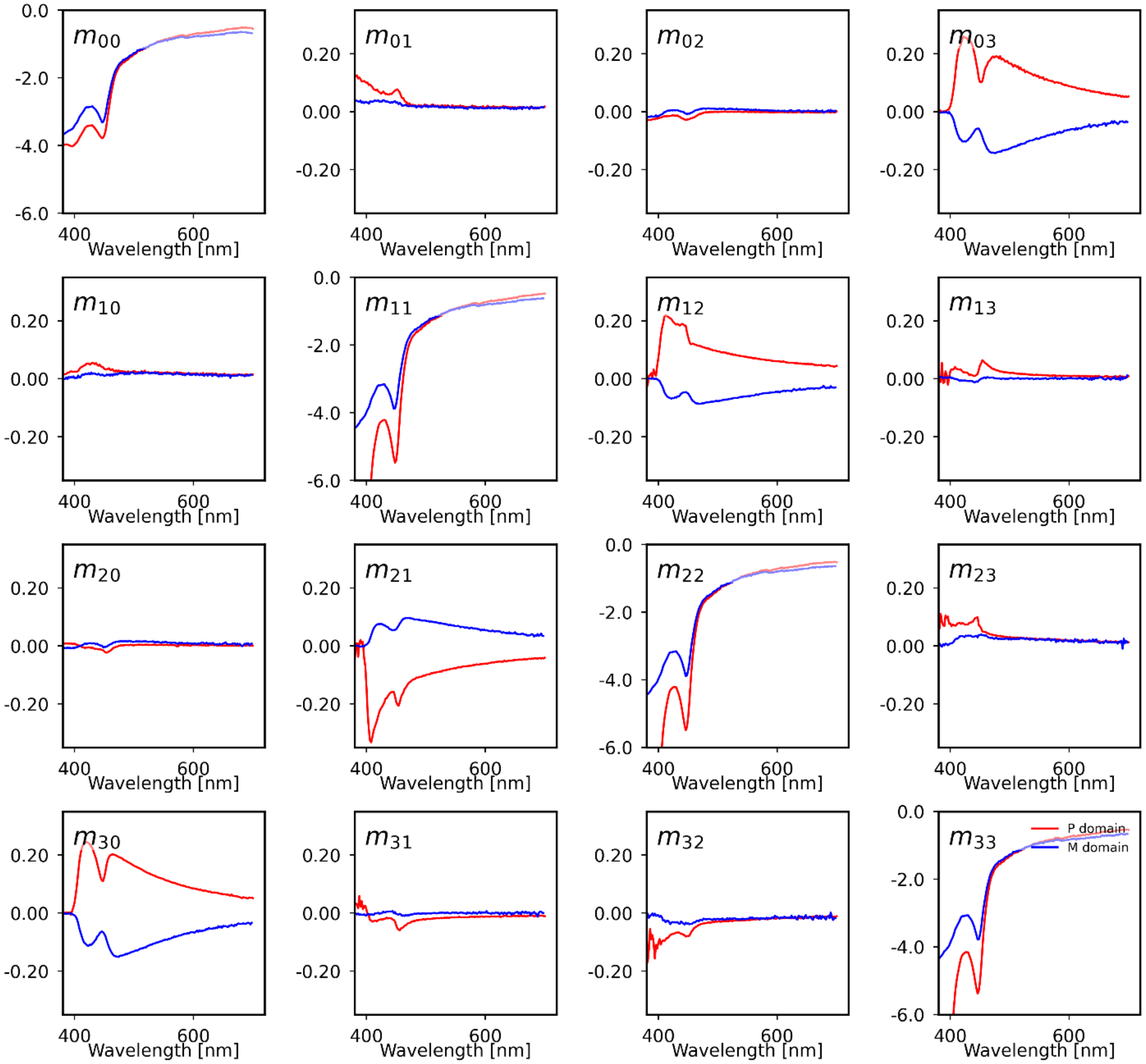

Figure S32. Differential Mueller Matrices calculated from data measured for a thin film of AZO doped with 80 µl of blue-NCs. The CD is given by the m_{03} and m_{30} elements of the differential Mueller matrix. Note that the matrix is almost perfectly G-antisymmetric that is $m_{03} = m_{30}$, $m_{12} = -m_{21}$, $m_{01} = m_{10}$, $m_{02} = m_{20}$, $m_{31} = -m_{13}$, $m_{23} = -m_{32}$, which confirms the reliable decomposition of the optical phenomena. Only at shorter wavelengths (below 450 nm), due to the sample's high absorption, some elements do not behave properly (i.e., $m_{21} \neq m_{12}$), but even there, the CD remains reliable as $m_{03} \approx m_{30}$.

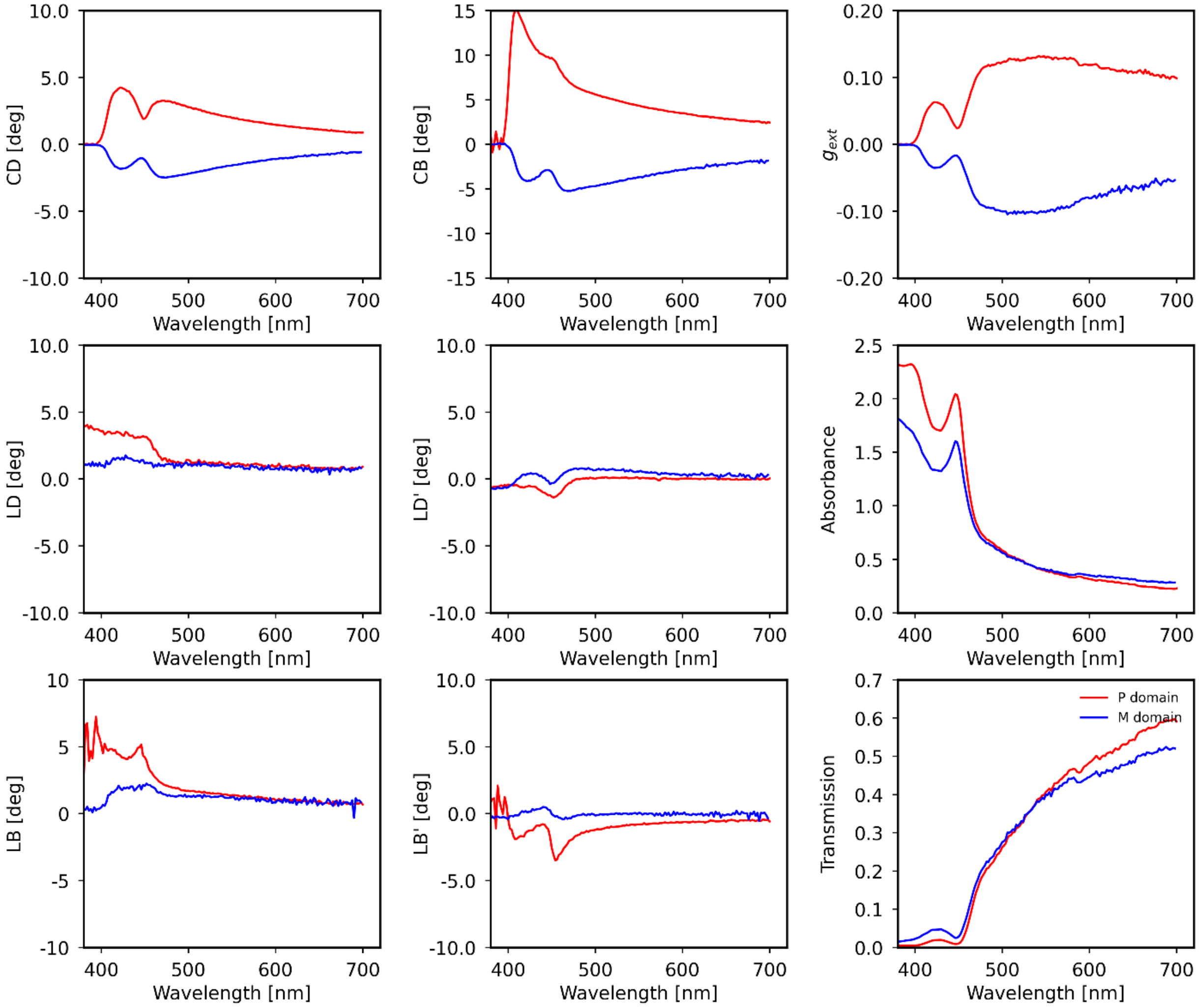

Figure S33. Elementary optical properties calculated from differential Mueller matrices obtained for thin film of AZO doped with 80 µl of blue-NCs (CD – circular dichroism, CB – circular birefringence, LD and LB – linear dichroism and linear birefringence measured between x and y axes of laboratory frame in the plane of sample, LD' and LB' – linear dichroism and linear birefringence measured between axes rotated by 45° around the optical axis relative to the laboratory frame in the plane of sample).

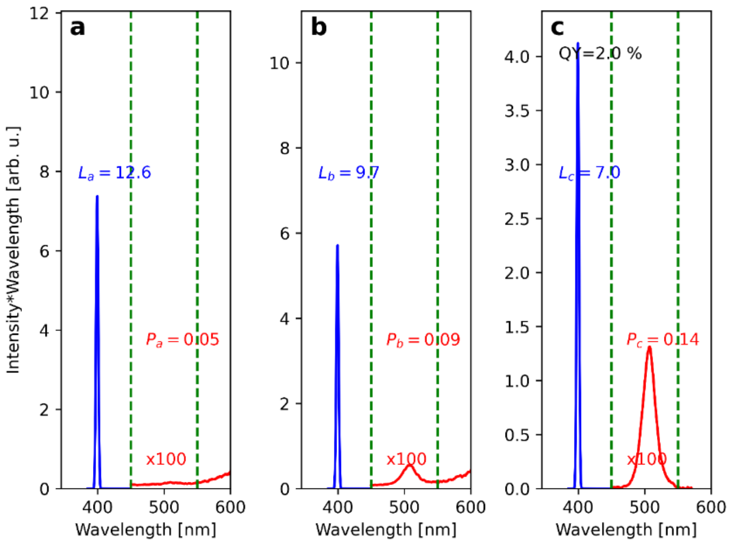

Figure S34. Spectra recorded in the integrating sphere for a 100 µl green-NCs sample in three conditions: a) no sample, b) with the sample but not in the path of the exciting beam, c) with the exciting beam directly illuminating the sample.

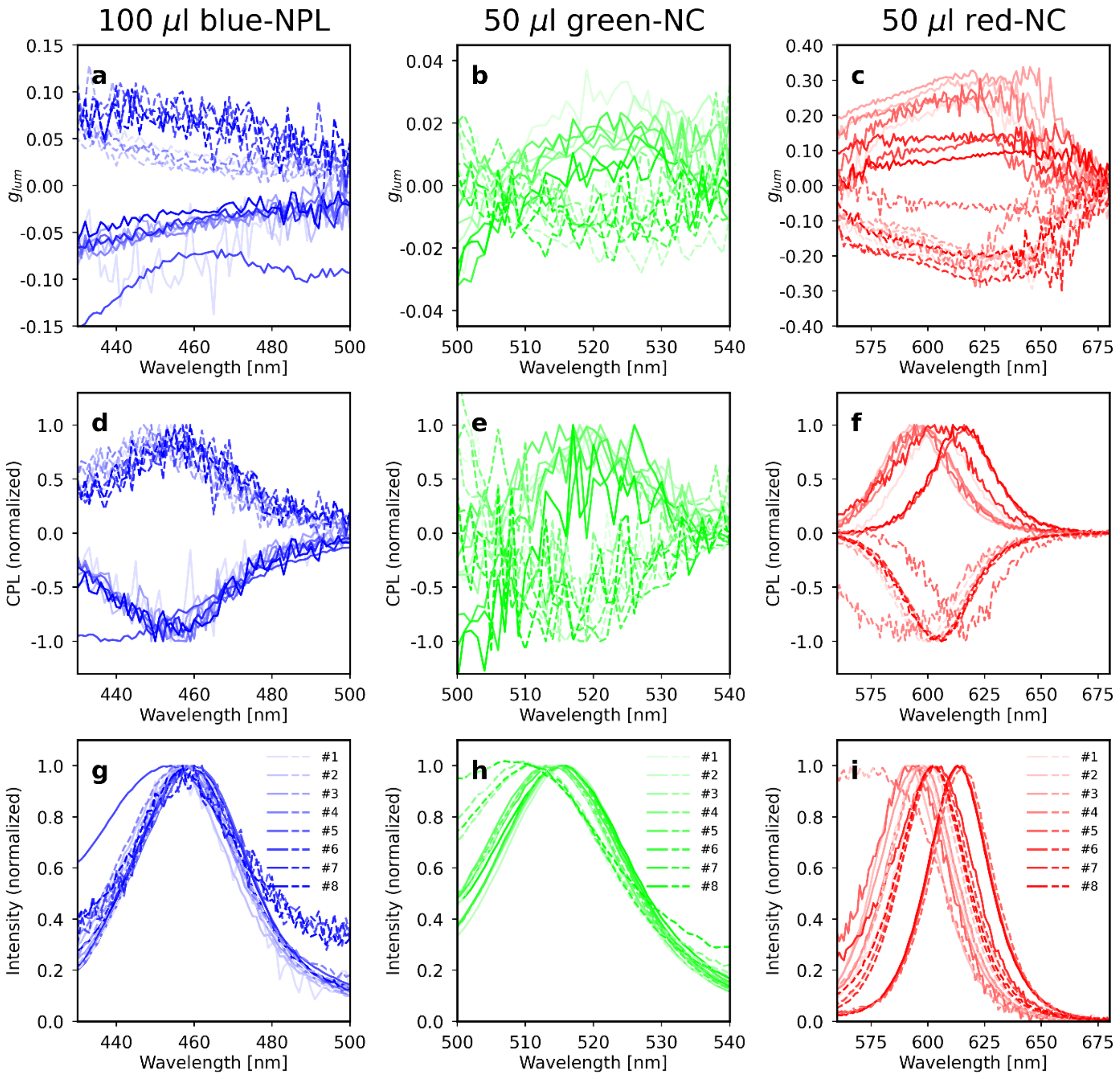

Figure S35. Emission spectra measured from 8 spots of AZO/PNCs films: a-c) dissymmetry factors, d-f) normalized CPL, g-i) normalized fluorescence spectra. Solid lines represent spots from P domain while dashed lines represent spots from M domain.

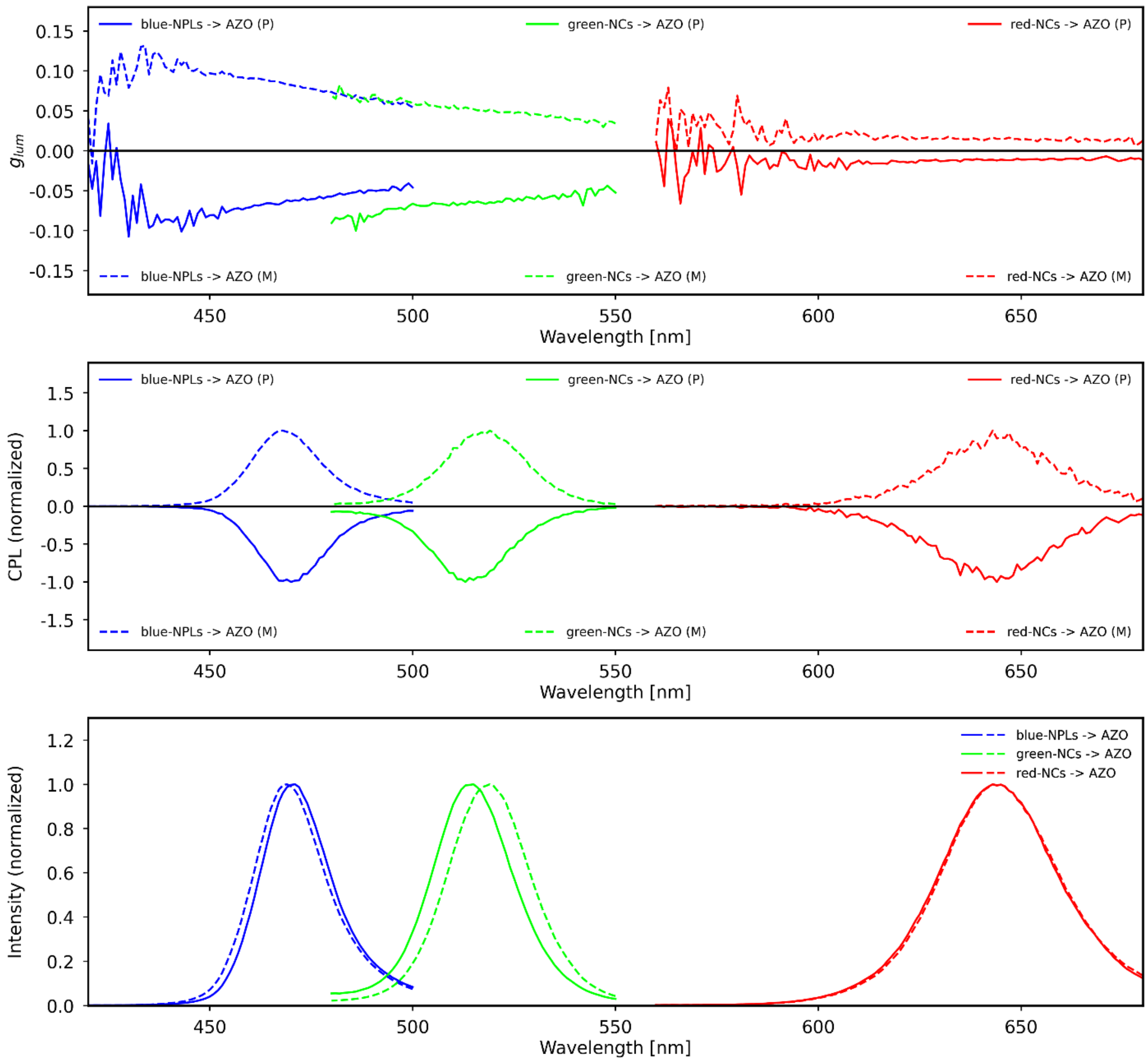

Figure S36. Emission spectra measured for pure PNCs thin films transmitted through separate pure AZO film, from top to bottom: dissymmetry factors, normalized CPL, normalized fluorescence spectra. Solid lines represent data emission transmitted by the P domain, and dashed lines represent emission transmitted by the M domain of AZO film.

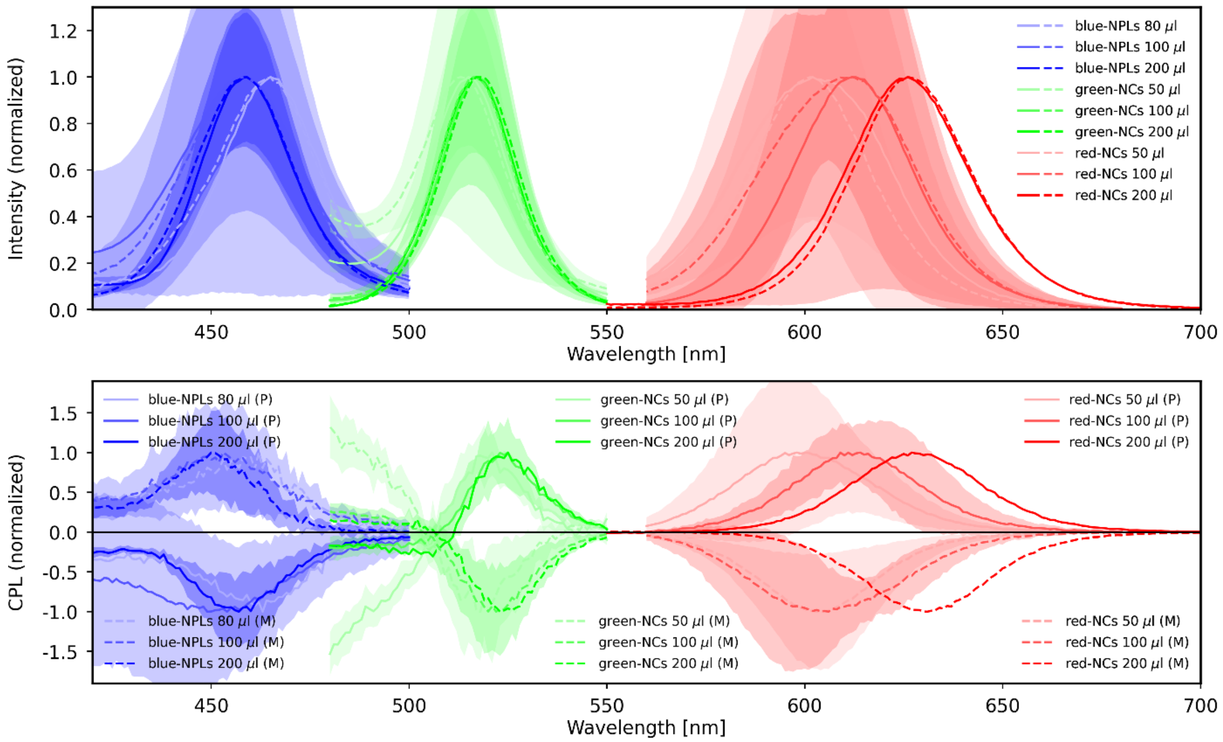

Figure S37. Averaged over 8 spots CPL spectra of AZO films doped with various amounts of PNCs. (a) emission spectra, (b) normalized CPL spectra. Note that the results for blue-, green- and red-NCs are combined into a single graph although coming from independent samples; the aim is to highlight the visible spectra range coverage and to ease comparing the sign of the CPL in a given type of domain handedness across the spectral range. Solid lines represent data measured from P domains, and dashed lines represent data from M domains. Line brightness increases with an increasing amount of PNCs doped in the sample. The shaded area in the g_{lum} graph represents the standard deviation of the measured values over 8 measurement spots.

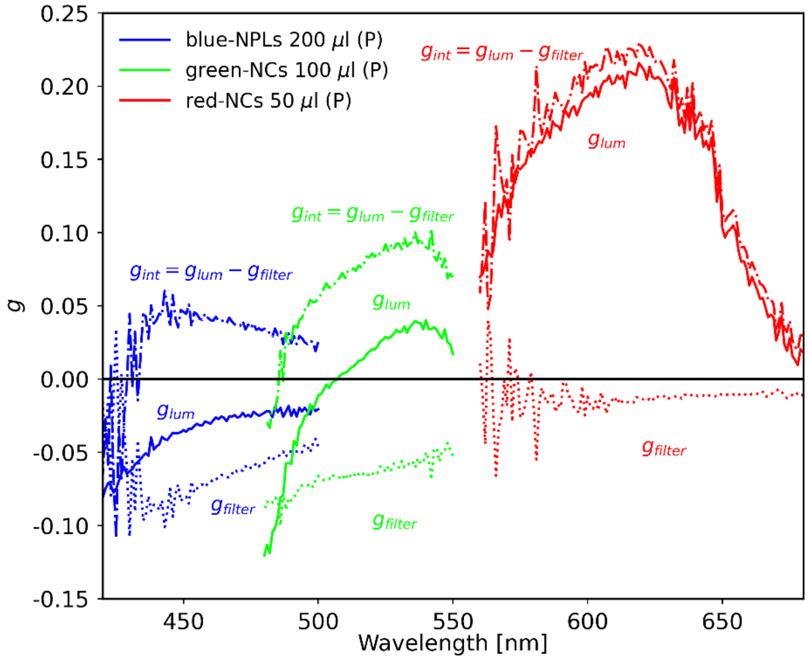

Figure S38. Disentangling intrinsic and reabsorption contributions in CPL from P domains of AZO films doped with PNCs: measured dissymmetry factors of ordinary samples (g_{lum}) and reabsorption components (g_{filter}), and reabsorption-free g_{int} spectra calculated using formula (1) from main text for films showing the highest dissymmetry for each type of PNCs.

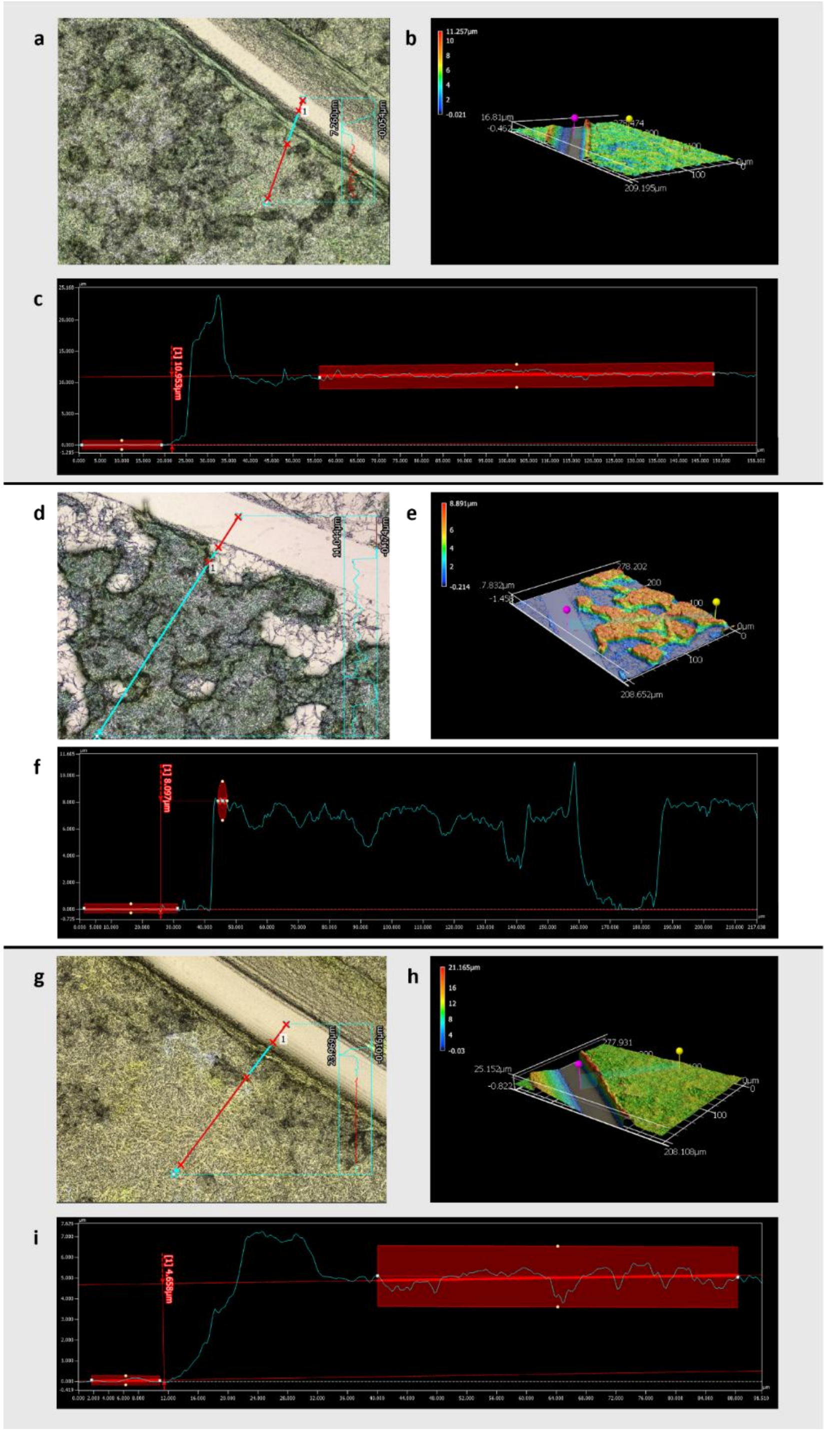

Figure S39. Representative profilometry measurements of AZO films after removal of the top glass substrate, used to determine the sample thickness in CPL experiments. Topography scans and height profiles confirm three thickness regimes: ≈4.5 µm (no spacer), ≈8 µm (silica sphere spacers), and ≈11 µm (aluminum foil spacers). The red arrows indicate the measurement paths across the film step edge.

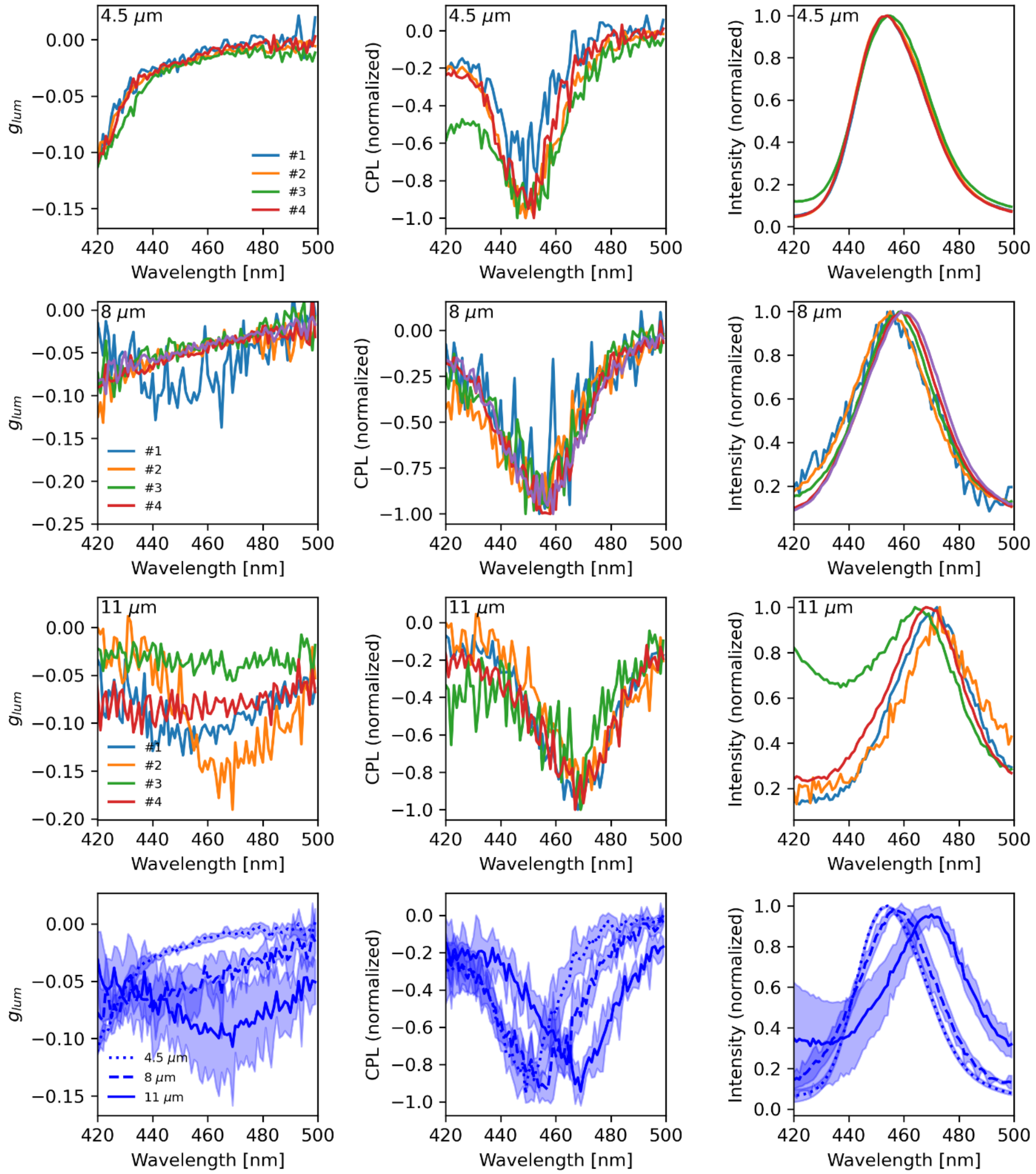

Figure S40. Thickness dependence of CPL of AZO matrix with 100 μL blue-NPL. The top three rows show results from four different spots on each sample. The bottom row presents the mean values (solid lines) with standard deviations (shaded area). Columns correspond to: g_{lum}, normalized CPL signal, and normalized emission intensity

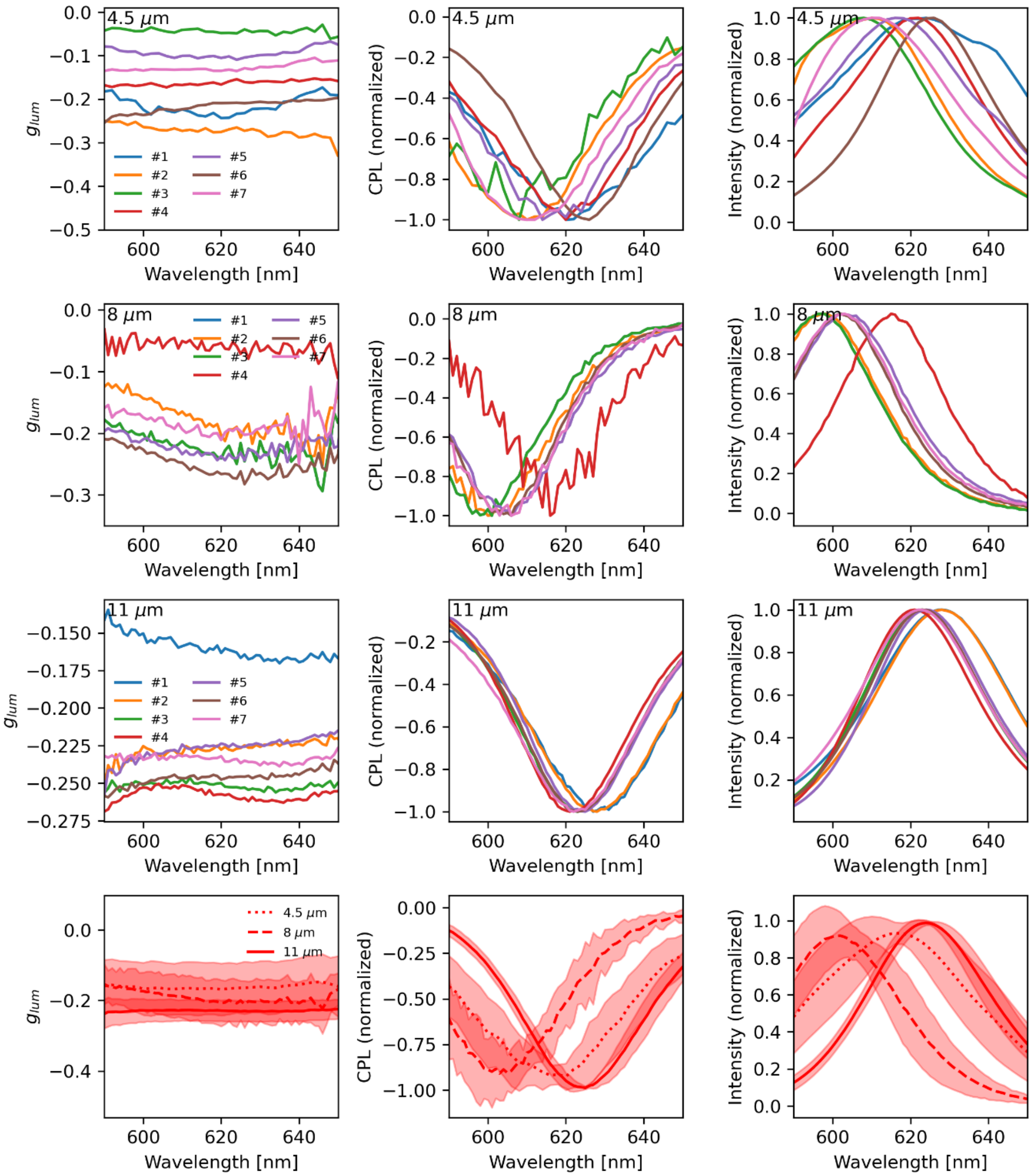

Figure S41. Thickness dependence of CPL of AZO matrix with 50 µL red-NC. The top three rows show results from four different spots on each sample. The bottom row presents the mean values (solid lines) with standard deviations (shaded area). Columns correspond to: g_{lum}, normalized CPL signal, and normalized emission intensity.

Bibliography

[50] T. Niori, S. Adachi, J. Watanabe, "Smectic mesophase properties of dimeric compounds. 1. Dimeric compounds based on the mesogenic azobenzene unit" *Liq Cryst* **1995**, *19*, 139–148.

[51] W. R. Kitzmann, J. Freudenthal, A. M. Reponen, Z. A. VanOrman, S. Feldmann, "Fundamentals, Advances, and Artifacts in Circularly Polarized Luminescence (CPL) Spectroscopy" *Advanced Materials* **2023**, *35*, 2302279.

[52] Y.-H. Kim, Y. Zhai, E. A. Gaulding, S. N. Habisreutinger, T. Moot, B. A. Rosales, H. Lu, A. Hazarika, R. Brunecky, L. M. Wheeler, J. J. Berry, M. C. Beard, J. M. Luther, "Strategies to Achieve High Circularly Polarized Luminescence from Colloidal Organic–Inorganic Hybrid Perovskite Nanocrystals" *ACS Nano* **2020**, *14*, 8816–8825.

[53] Y. Shi, P. Duan, S. Huo, Y. Li, M. Liu, "Endowing Perovskite Nanocrystals with Circularly Polarized Luminescence" *Advanced Materials* **2018**, *30*, 201705011.

[54] M. Kim, J. Kim, J. Bang, Y. J. Jang, J. Park, D. H. Kim, "Simultaneously achieving room-temperature circularly polarized luminescence and high stability in chiral perovskite nanocrystals *via* block copolymer micellar nanoreactors" *J Mater Chem A Mater* **2023**, *11*, 12876–12884.

[55] X. Zhang, L. Li, Y. Chen, C. Valenzuela, Y. Liu, Y. Yang, Y. Feng, L. Wang, W. Feng, "Mechanically Tunable Circularly Polarized Luminescence of Liquid Crystal-Templated Chiral Perovskite Quantum Dots" *Angewandte Chemie International Edition* **2024**, *63*, e202404202.

[56] Y. Wang, M.-S. Song, J. Zhao, Z. Li, T. Wang, H. Wang, H.-Y. Wang, Y. Wang, "Chiral Perovskite Heterostructure Films of $CsPbBr_3$ Quantum Dots and 2D Chiral Perovskite with Circularly Polarized Luminescence Performance and Energy Transfer" *ACS Nano* **2024**, *18*, 22334–22343.

[57] J. Wang, C. Fang, J. Ma, S. Wang, L. Jin, W. Li, D. Li, "Aqueous Synthesis of Low-Dimensional Lead Halide Perovskites for Room-Temperature Circularly Polarized Light Emission and Detection" *ACS Nano* **2019**, *13*, 9473–9481.

[58] B. Zhao, X. Gao, K. Pan, J. Deng, "Chiral Helical Polymer/Perovskite Hybrid Nanofibers with Intense Circularly Polarized Luminescence" *ACS Nano* **2021**, *15*, 7463–7471.

[59] C. Zhao, H. Zhao, Z. Chen, R. Pei, P. Wang, "Circularly Polarized Luminescence from Chiral Photonic Crystals of Caesium Lead Halide Perovskites" *Adv Mater Interfaces* **2023**, *10*, DOI 10.1002/admi.202300576.

[60] J. Mendoza-Carreño, P. Molet, C. Otero-Martínez, M. I. Alonso, L. Polavarapu, A. Mihi, "Nanoimprinted 2D-Chiral Perovskite Nanocrystal Metasurfaces for Circularly Polarized Photoluminescence" *Advanced Materials* **2023**, 2210477.

[61] C. Wang, K. Chen, P. Xu, F. Yeung, H. Kwok, G. Li, "Fully Chiral Light Emission from $CsPbX_3$ Perovskite Nanocrystals Enabled by Cholesteric Superstructure Stacks" *Adv Funct Mater* **2019**, *29*, 201903155.

[62] R. Momper, H. Zhang, S. Chen, H. Halim, E. Johannes, S. Yordanov, D. Braga, B. Blülle, D. Doblas, T. Kraus, M. Bonn, H. I. Wang, A. Riedinger, "Kinetic Control over Self-Assembly of Semiconductor Nanoplatelets" *Nano Lett* **2020**, *20*, 4102–4110.

[63] S. Liu, X. Liu, Y. Wu, D. Zhang, Y. Wu, H. Tian, Z. Zheng, W.-H. Zhu, "Circularly polarized perovskite luminescence with dissymmetry factor up to 1.9 by soft helix bilayer device" *Matter* **2022**, *5*, 2319–2333.

[64] R. M. A. Azzam, “Propagation of partially polarized light through anisotropic media with or without depolarization: A differential 4 × 4 matrix calculus” *J Opt Soc Am* **1978**, *68*, 1756.
